# Supplementary figures and images for: Benchmarking principal component analysis for large-scale single-cell RNA-sequencing
Source: Genome Biol. 2020 Jan 20;21:9. doi: 10.1186/s13059-019-1900-3 (PMC6970290; doi:10.1186/s13059-019-1900-3)

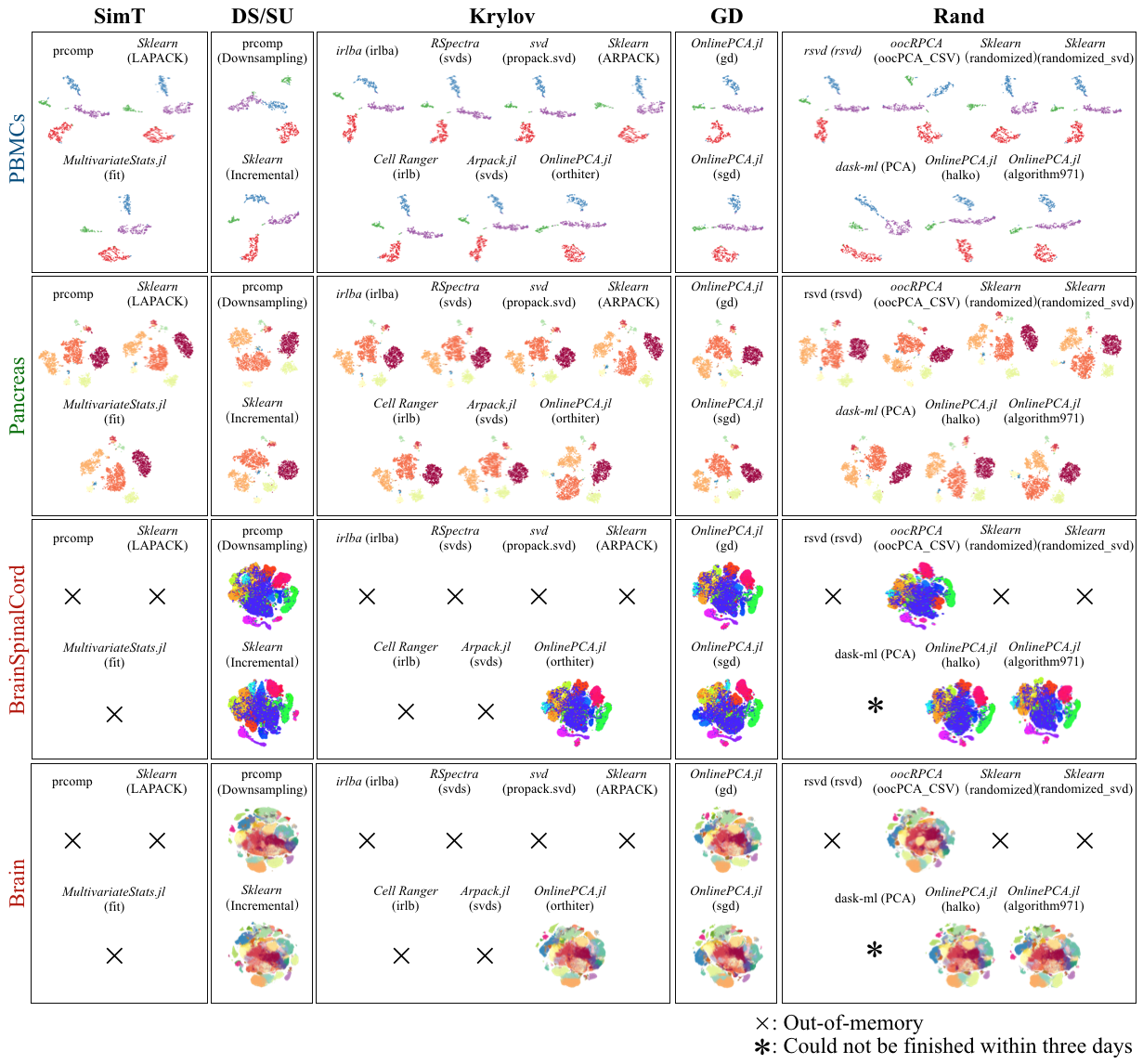

Supplement: Supplementary file 4 — Additional file 4 Results of t-SNE of all the pCA implementations. [file 13059_2019_1900_MOESM4_ESM.png]

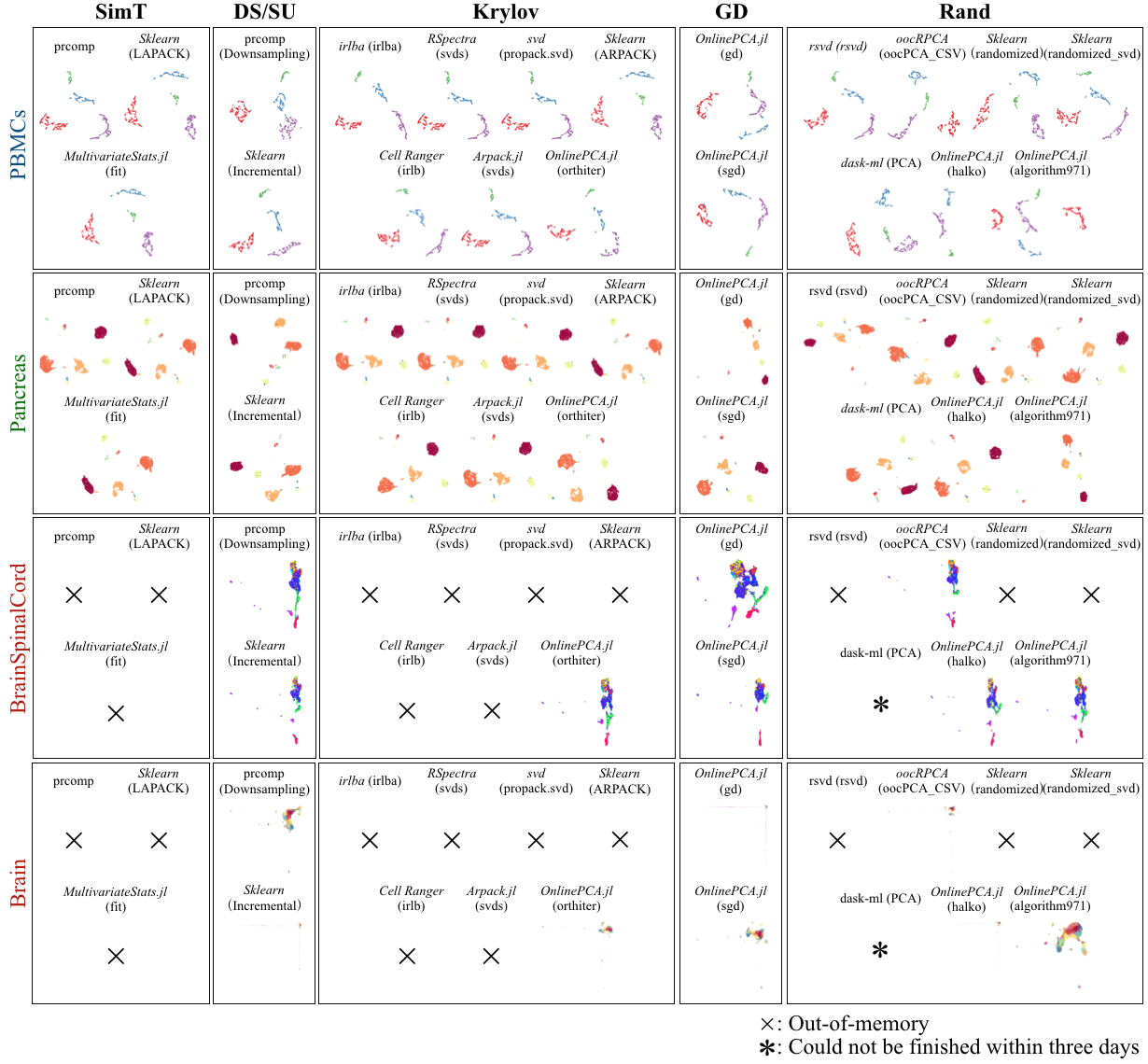

Supplement: Supplementary file 5 — Additional file 5 Results of uMAP of all the pCA implementations. [file 13059_2019_1900_MOESM5_ESM.png]

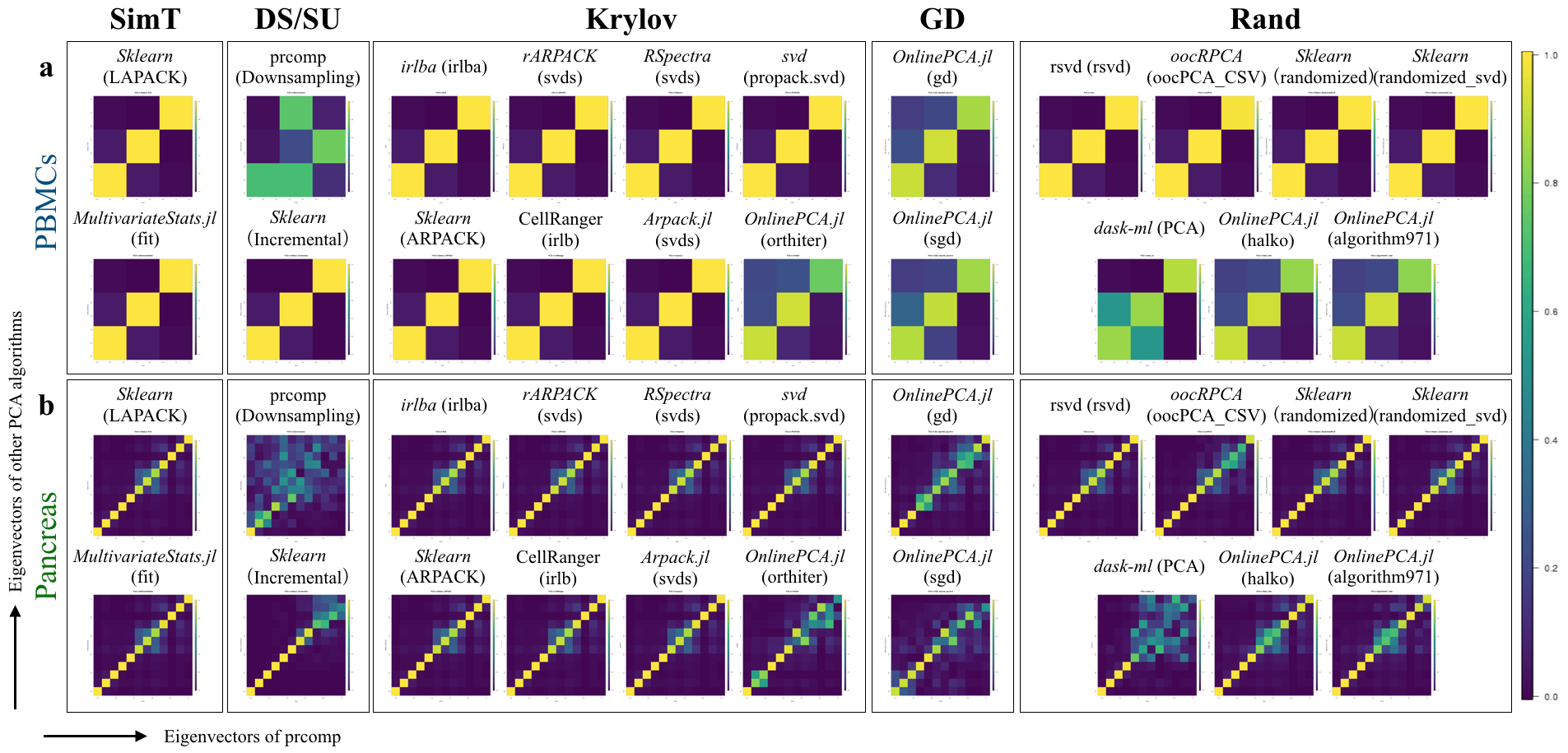

Supplement: Supplementary file 7 — Additional file 7 Eigenvectors of all the pCA implementations (PBMCs and pancreas). [file 13059_2019_1900_MOESM7_ESM.png]

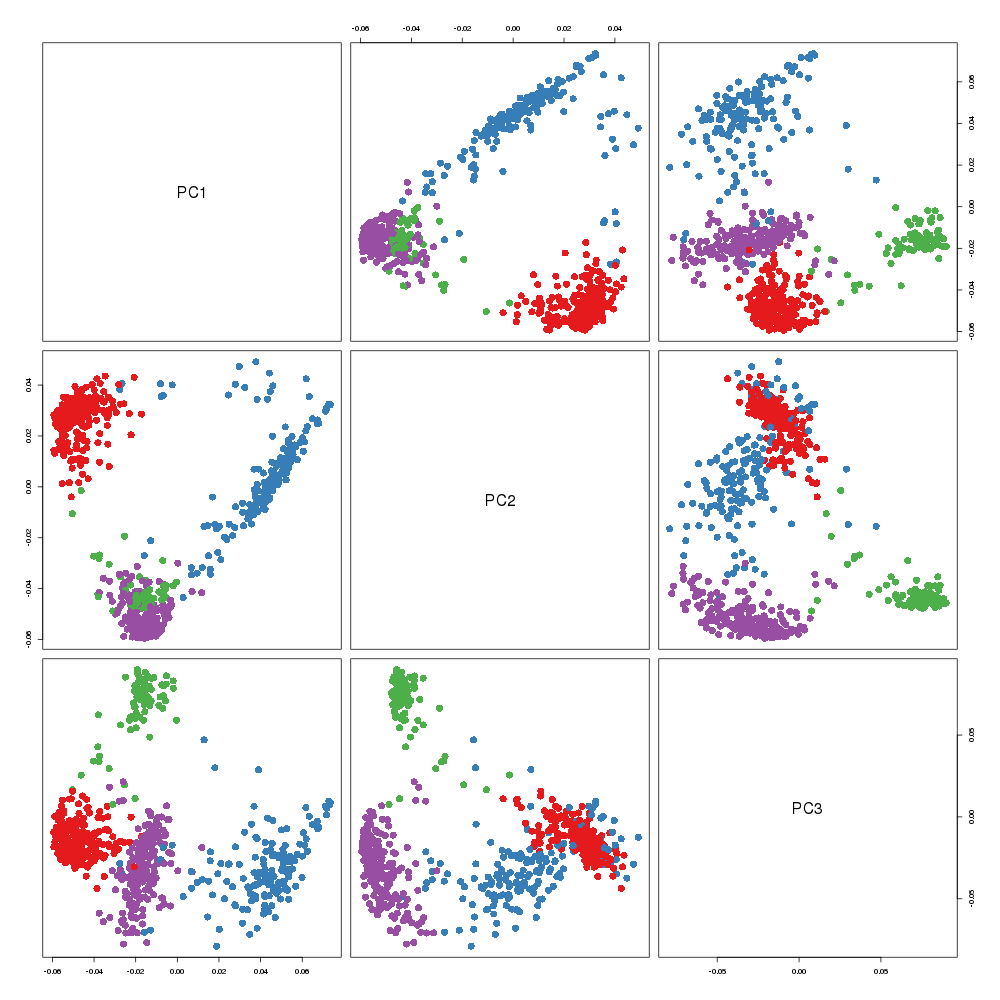

Supplement: Supplementary file 8 — Additional file 8 Pair plots of all the pCA (PBMCs) implementations. [file 13059_2019_1900_MOESM8_ESM.gz › AdditionalFile8/GD_step1000_epoch10.png]

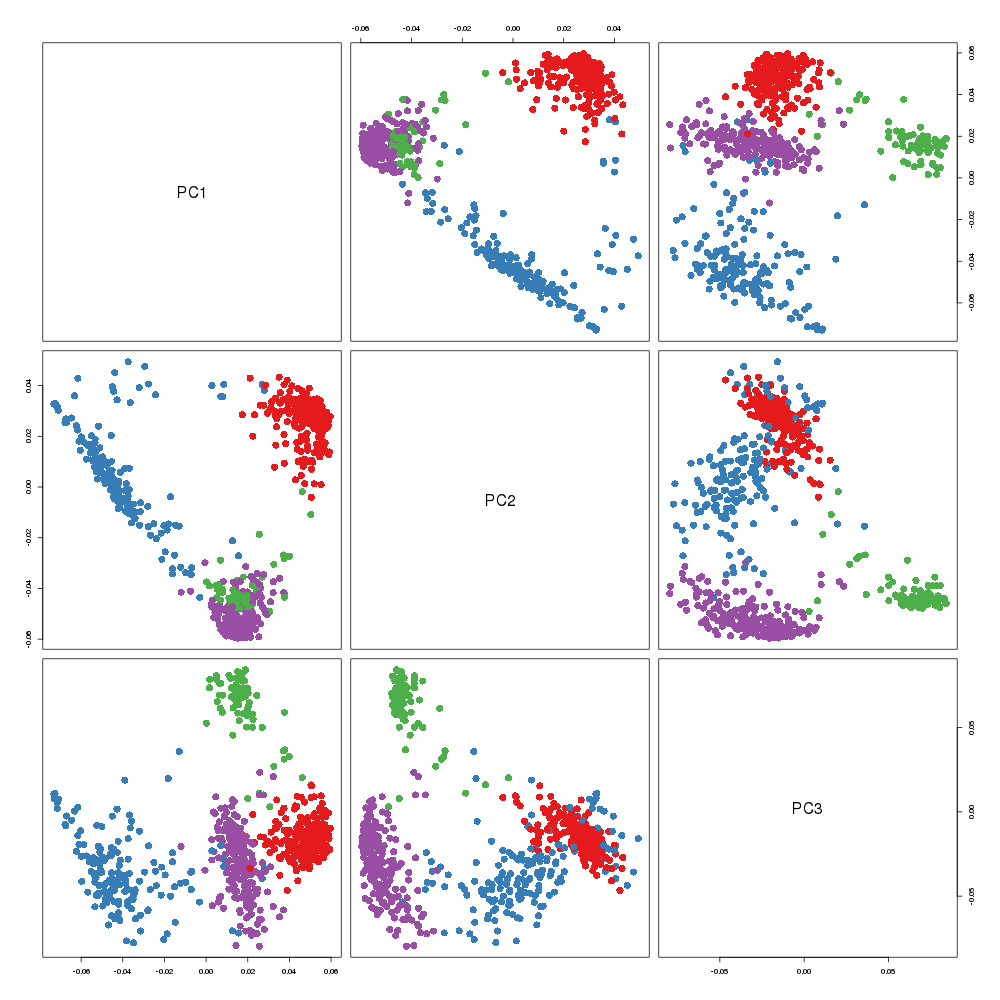

Supplement: Supplementary file 8 — Additional file 8 Pair plots of all the pCA (PBMCs) implementations. [file 13059_2019_1900_MOESM8_ESM.gz › AdditionalFile8/Algorithm971_3iter.png]

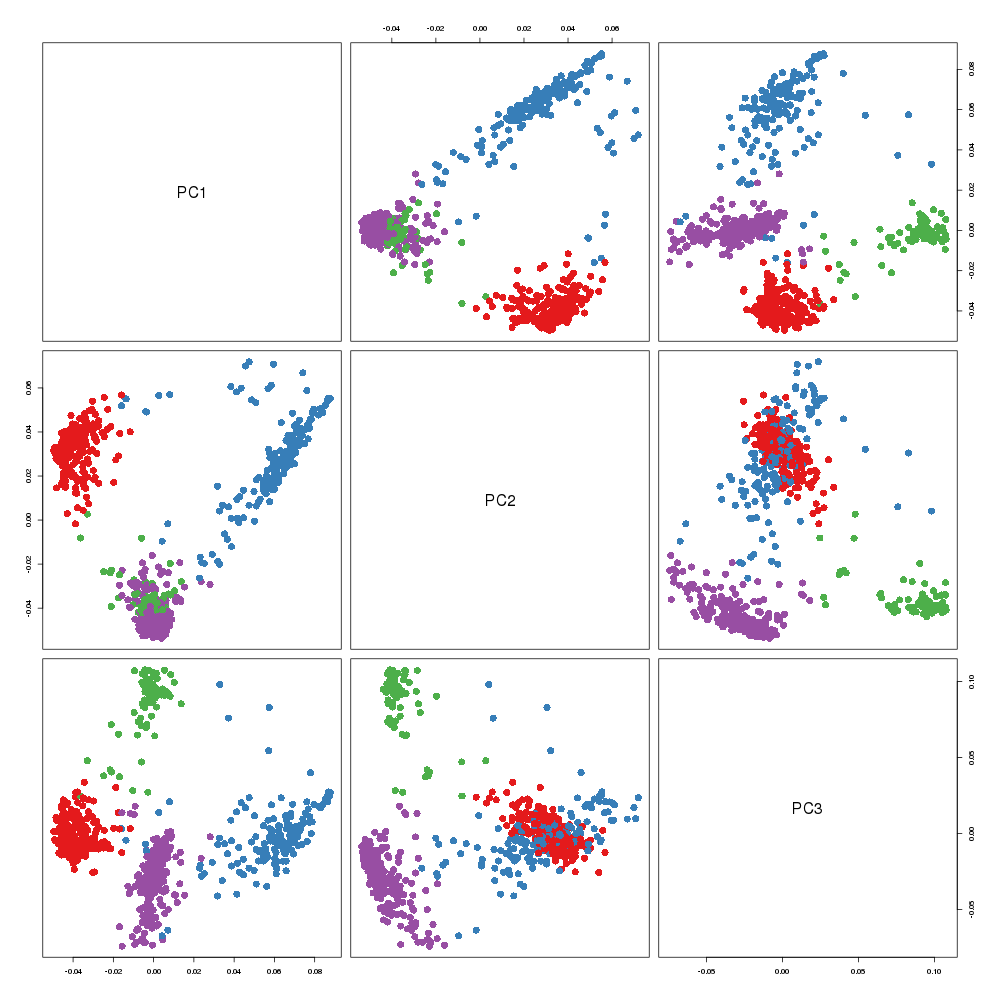

Supplement: Supplementary file 8 — Additional file 8 Pair plots of all the pCA (PBMCs) implementations. [file 13059_2019_1900_MOESM8_ESM.gz › AdditionalFile8/CellRanger.png]

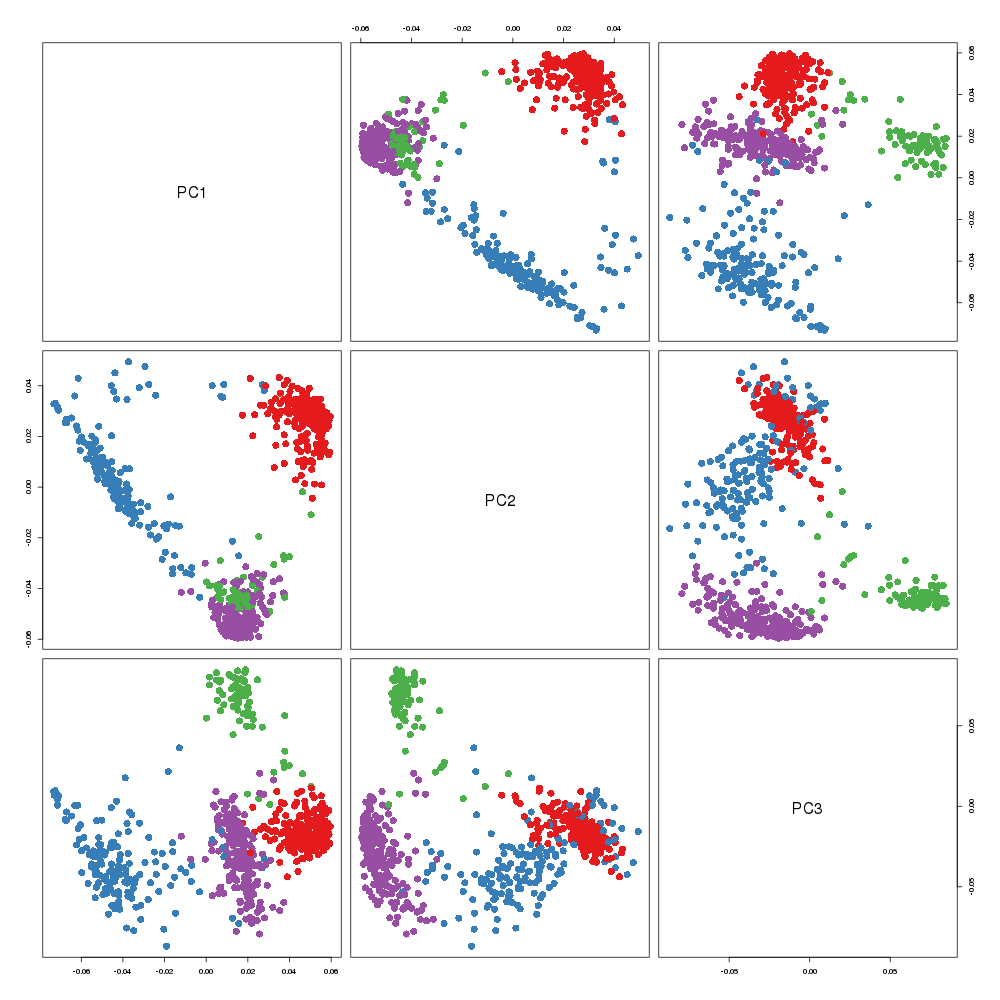

Supplement: Supplementary file 8 — Additional file 8 Pair plots of all the pCA (PBMCs) implementations. [file 13059_2019_1900_MOESM8_ESM.gz › AdditionalFile8/Halko_2iter.png]

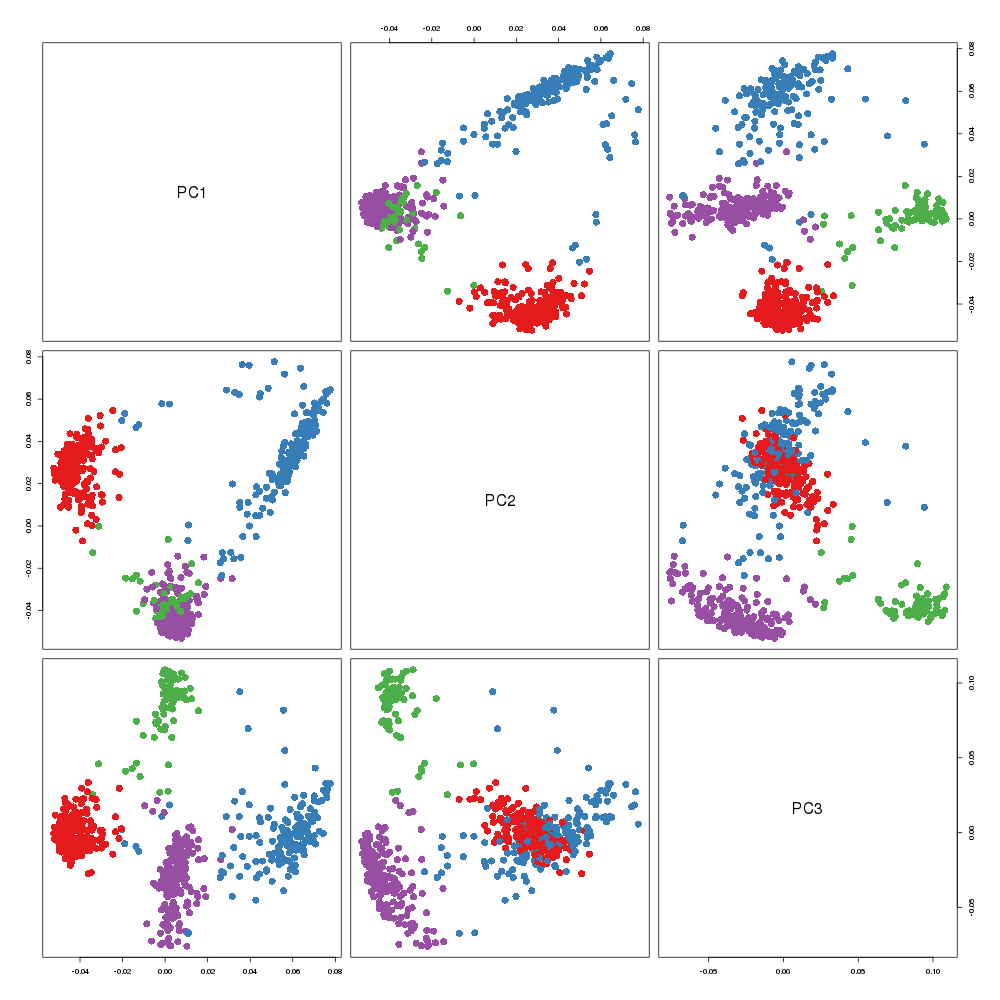

Supplement: Supplementary file 8 — Additional file 8 Pair plots of all the pCA (PBMCs) implementations. [file 13059_2019_1900_MOESM8_ESM.gz › AdditionalFile8/Sklearn_Incremental.png]

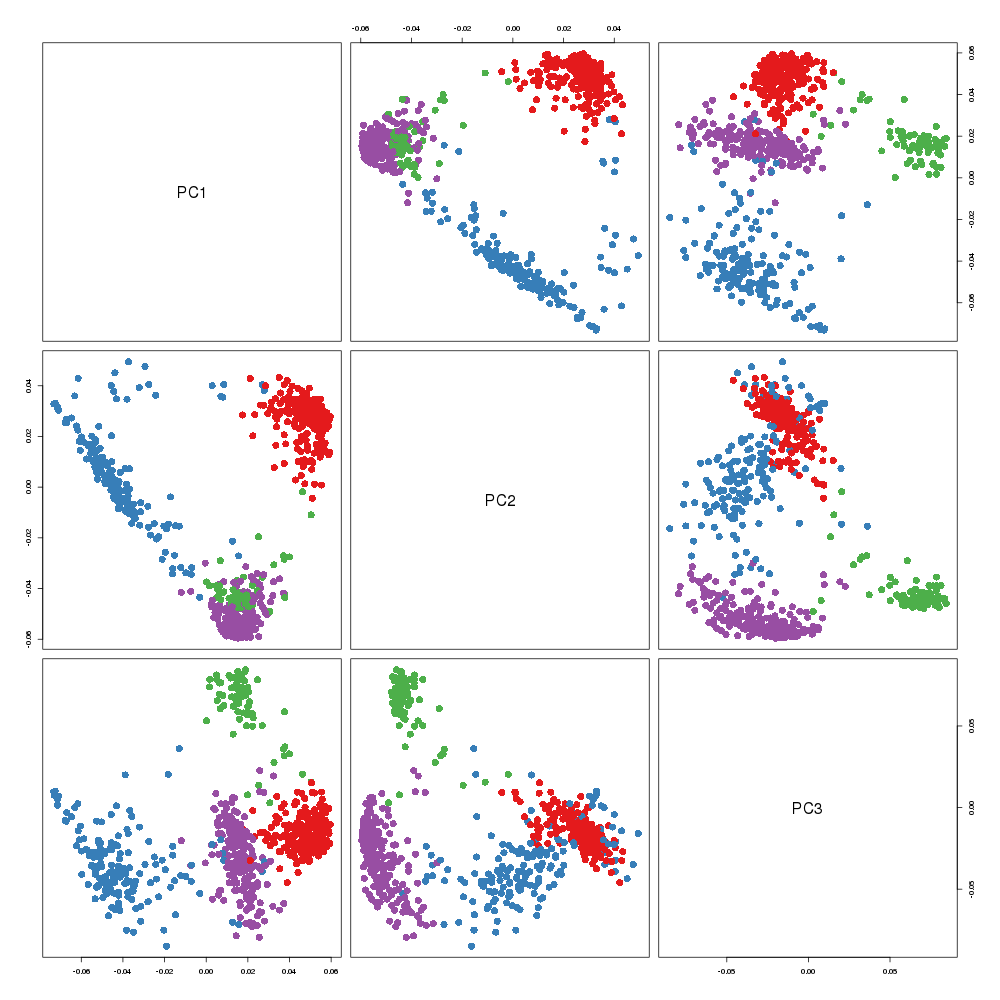

Supplement: Supplementary file 8 — Additional file 8 Pair plots of all the pCA (PBMCs) implementations. [file 13059_2019_1900_MOESM8_ESM.gz › AdditionalFile8/Halko_3iter.png]

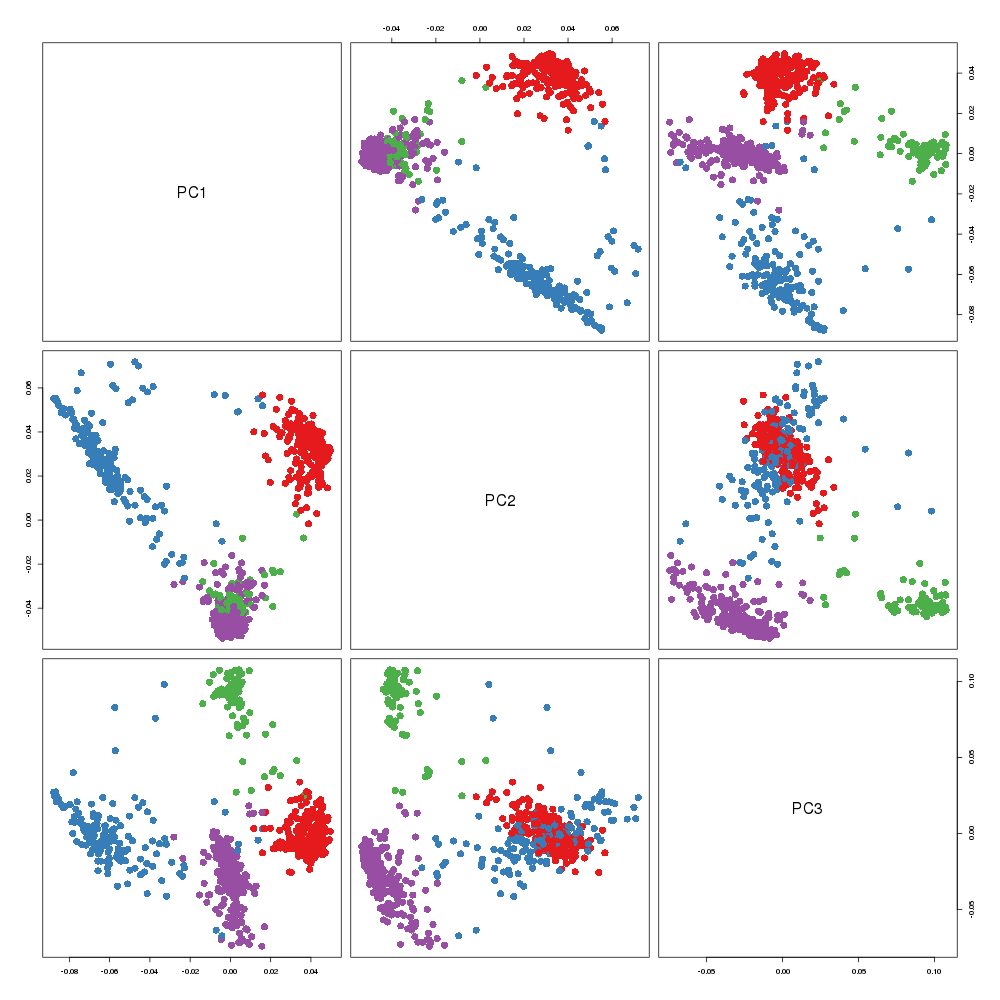

Supplement: Supplementary file 8 — Additional file 8 Pair plots of all the pCA (PBMCs) implementations. [file 13059_2019_1900_MOESM8_ESM.gz › AdditionalFile8/Sklearn_randomized_svd.png]

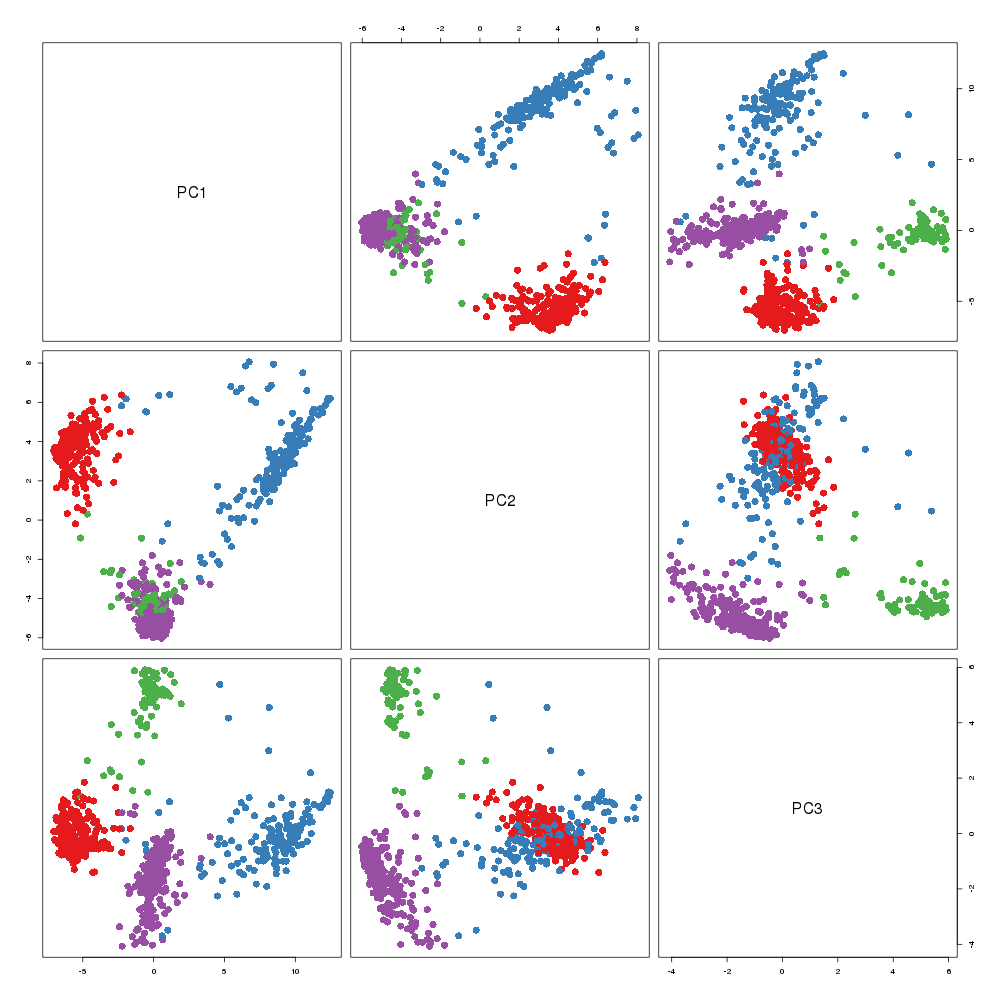

Supplement: Supplementary file 8 — Additional file 8 Pair plots of all the pCA (PBMCs) implementations. [file 13059_2019_1900_MOESM8_ESM.gz › AdditionalFile8/Sklearn_ARPACK.png]

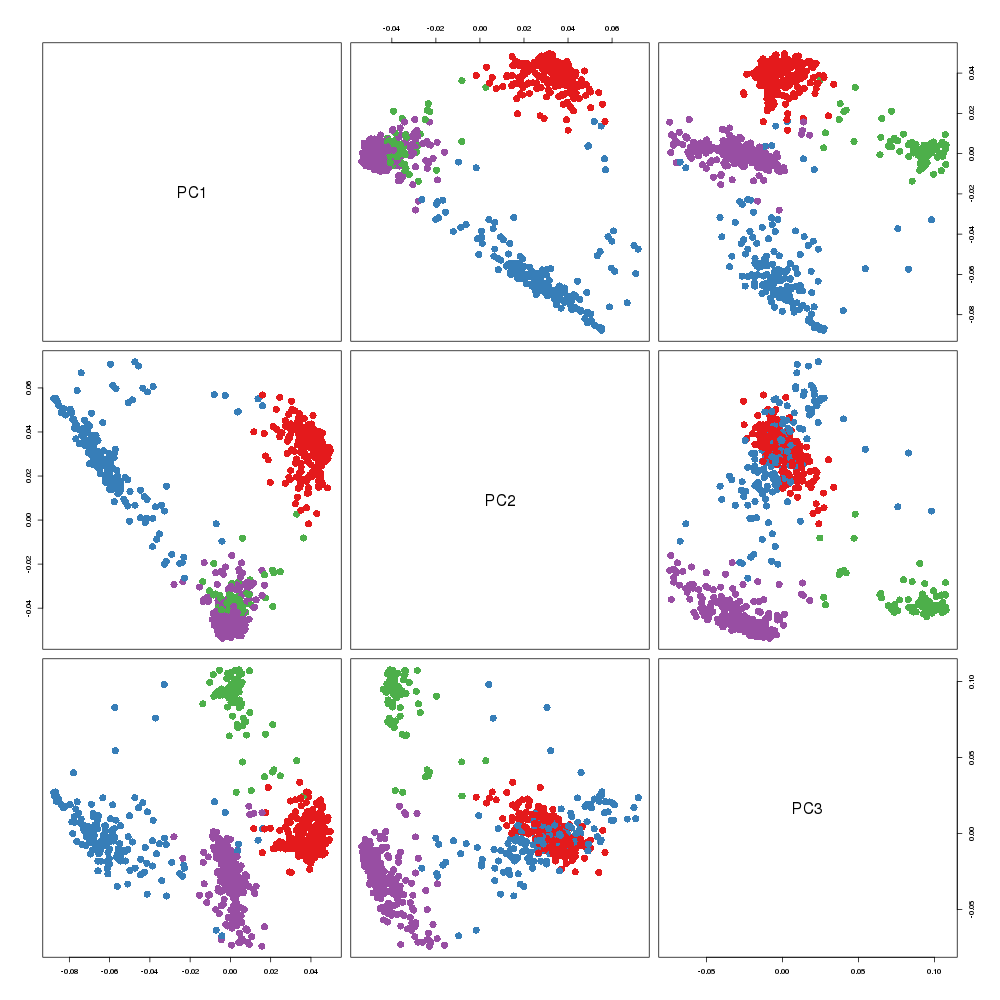

Supplement: Supplementary file 8 — Additional file 8 Pair plots of all the pCA (PBMCs) implementations. [file 13059_2019_1900_MOESM8_ESM.gz › AdditionalFile8/PROPACK.png]

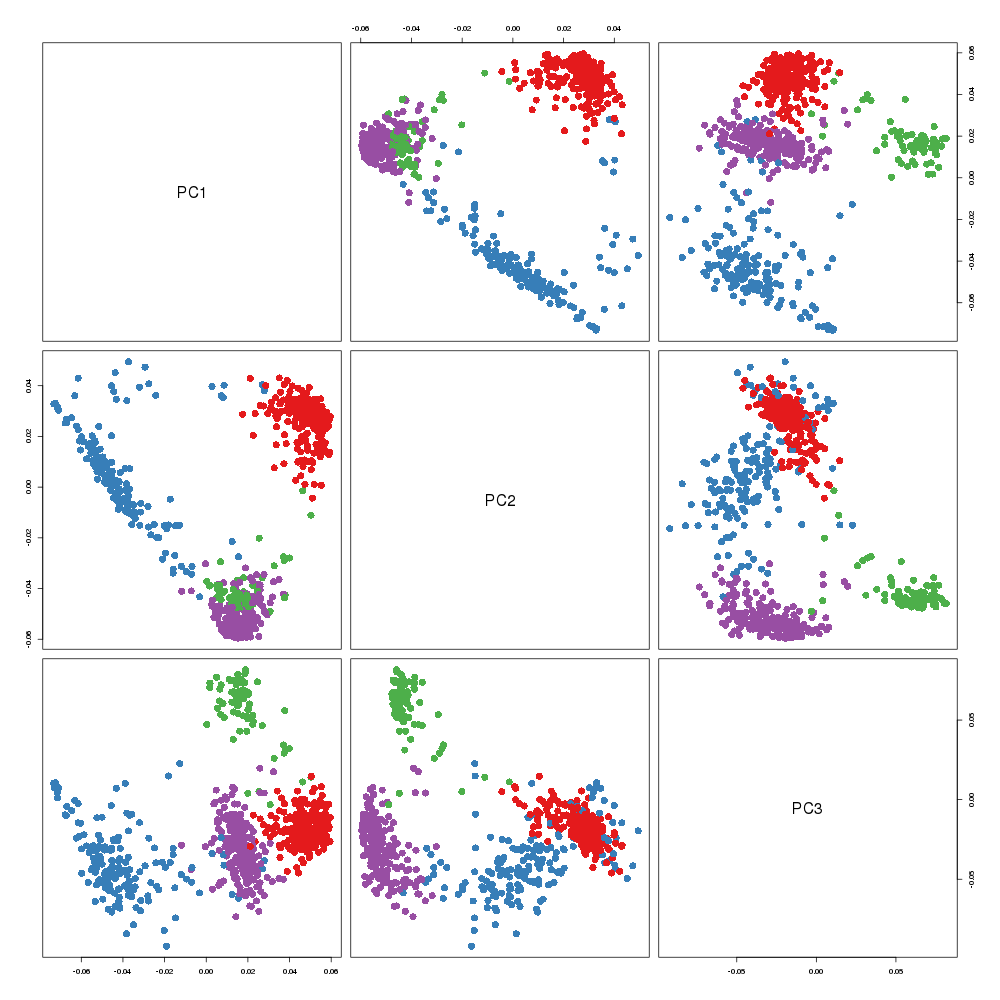

Supplement: Supplementary file 8 — Additional file 8 Pair plots of all the pCA (PBMCs) implementations. [file 13059_2019_1900_MOESM8_ESM.gz › AdditionalFile8/Halko_1iter.png]

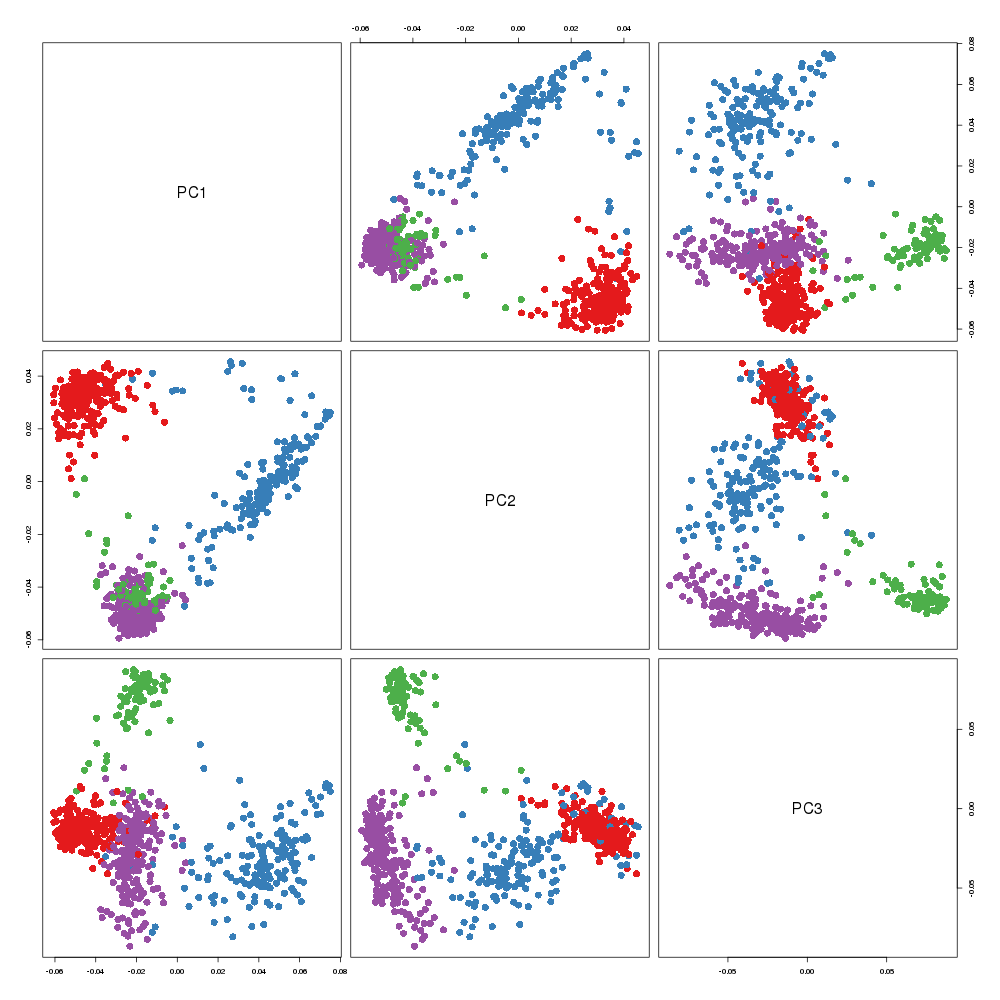

Supplement: Supplementary file 8 — Additional file 8 Pair plots of all the pCA (PBMCs) implementations. [file 13059_2019_1900_MOESM8_ESM.gz › AdditionalFile8/SGD_step100_epoch10.png]

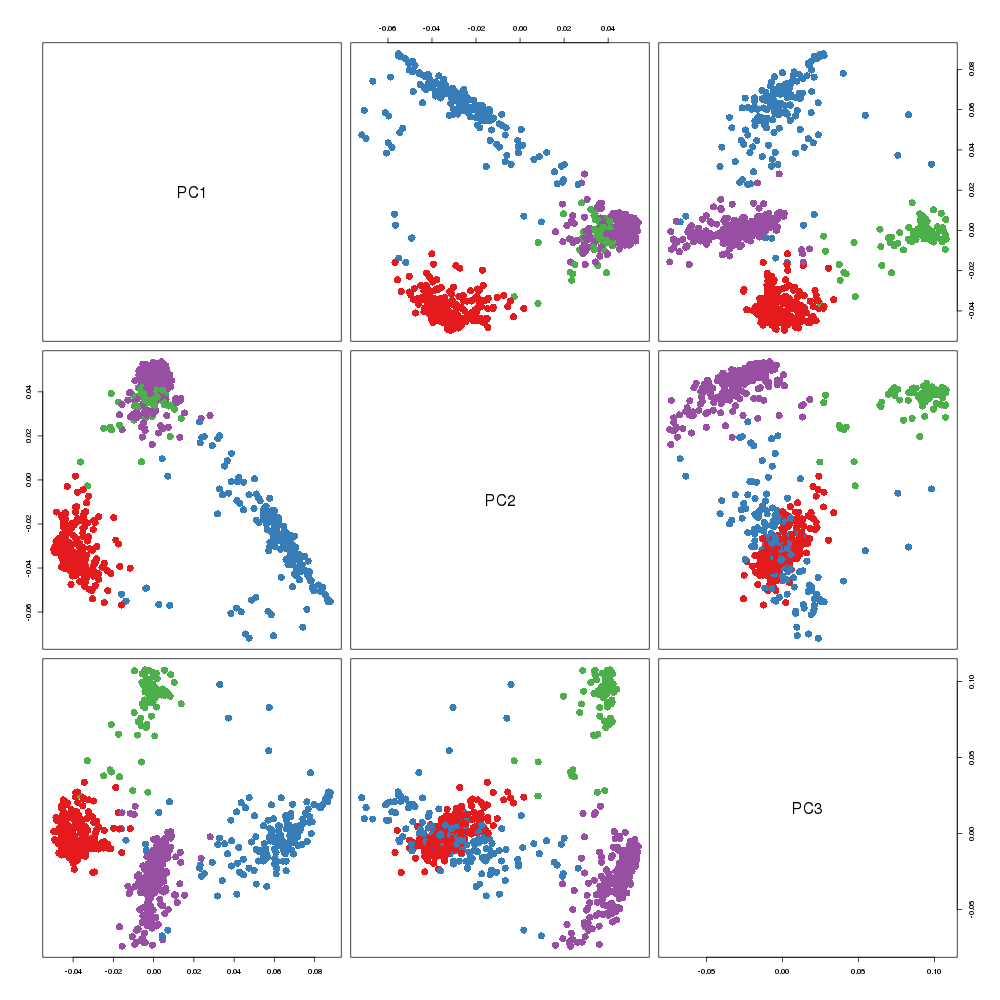

Supplement: Supplementary file 8 — Additional file 8 Pair plots of all the pCA (PBMCs) implementations. [file 13059_2019_1900_MOESM8_ESM.gz › AdditionalFile8/RSpectra.png]

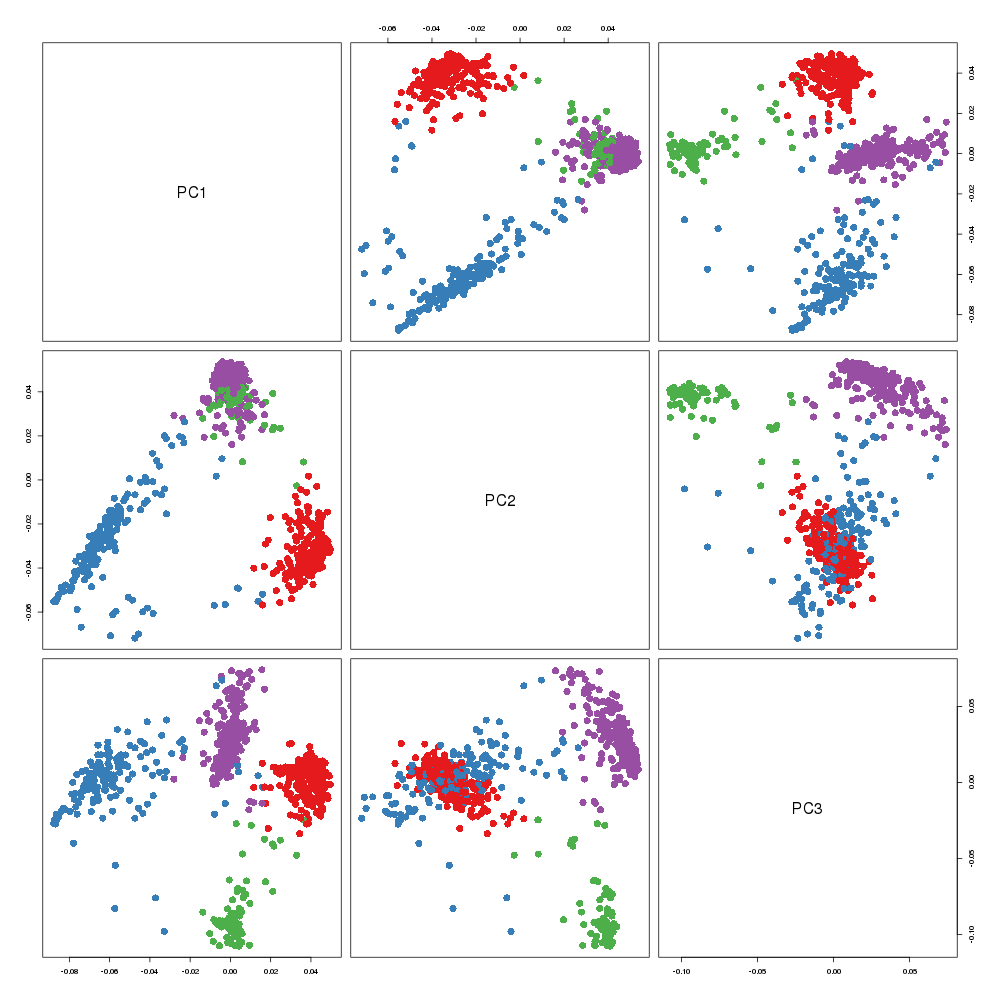

Supplement: Supplementary file 8 — Additional file 8 Pair plots of all the pCA (PBMCs) implementations. [file 13059_2019_1900_MOESM8_ESM.gz › AdditionalFile8/Arpack.jl.png]

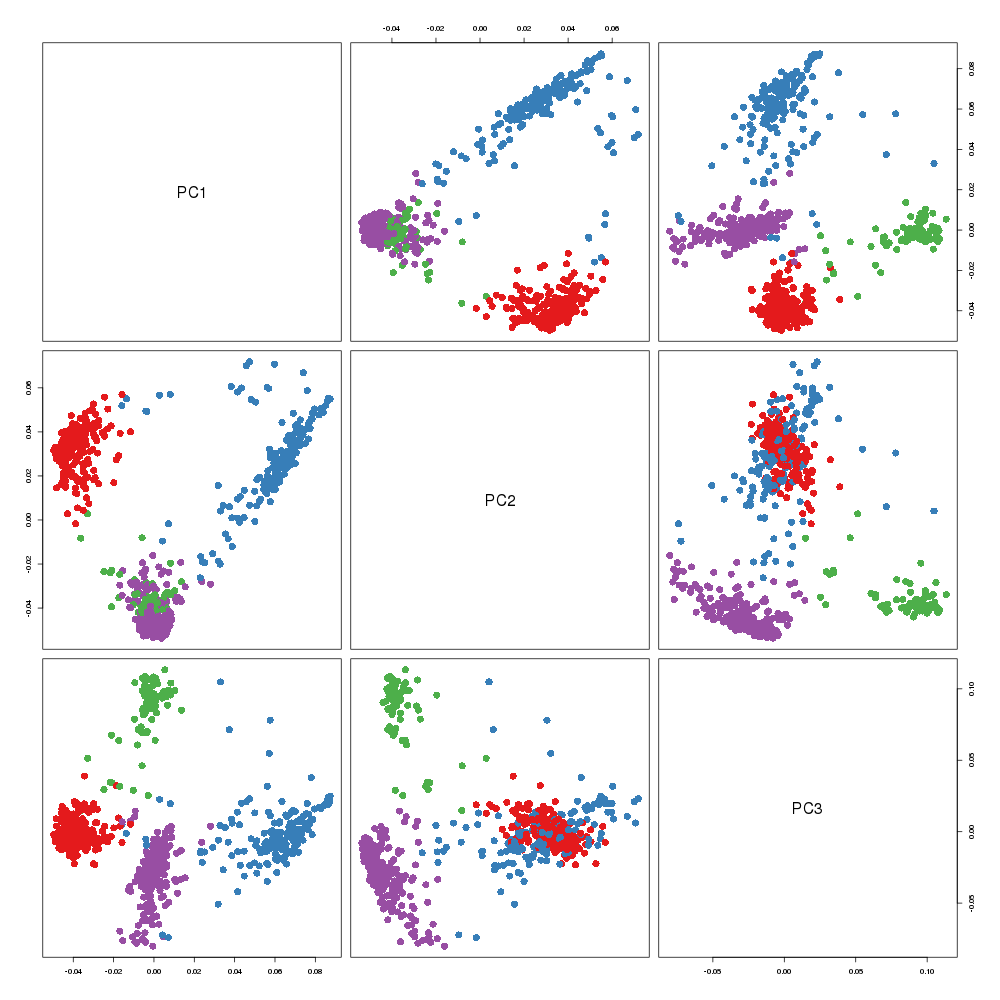

Supplement: Supplementary file 8 — Additional file 8 Pair plots of all the pCA (PBMCs) implementations. [file 13059_2019_1900_MOESM8_ESM.gz › AdditionalFile8/oocRPCA.png]

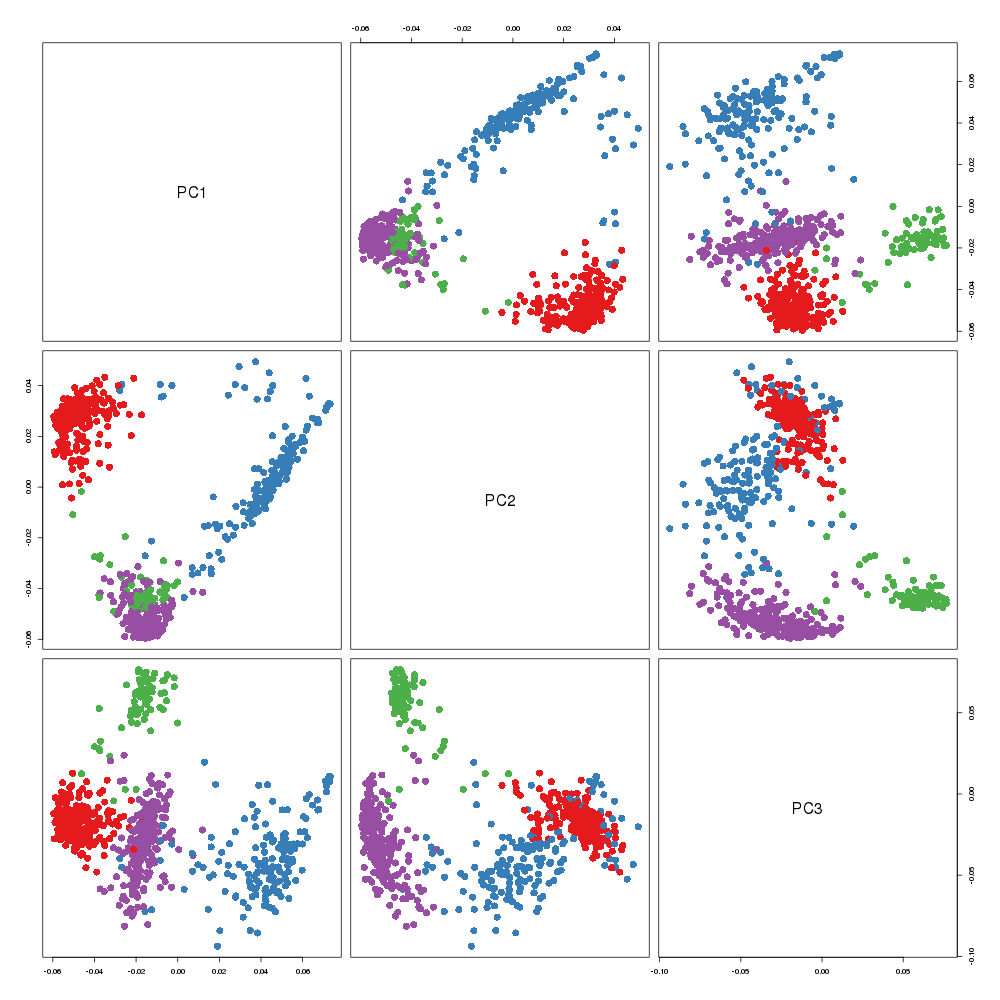

Supplement: Supplementary file 8 — Additional file 8 Pair plots of all the pCA (PBMCs) implementations. [file 13059_2019_1900_MOESM8_ESM.gz › AdditionalFile8/OrthIter.png]

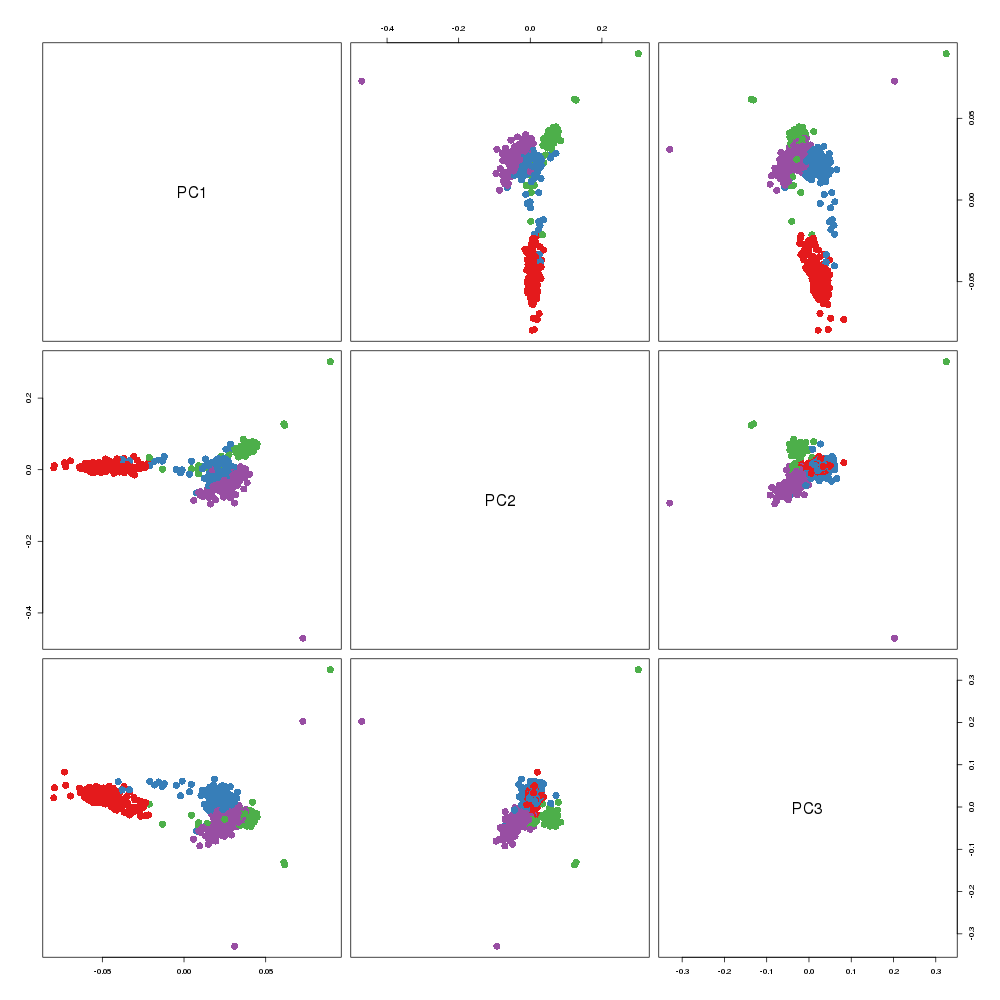

Supplement: Supplementary file 8 — Additional file 8 Pair plots of all the pCA (PBMCs) implementations. [file 13059_2019_1900_MOESM8_ESM.gz › AdditionalFile8/Downsampling.png]

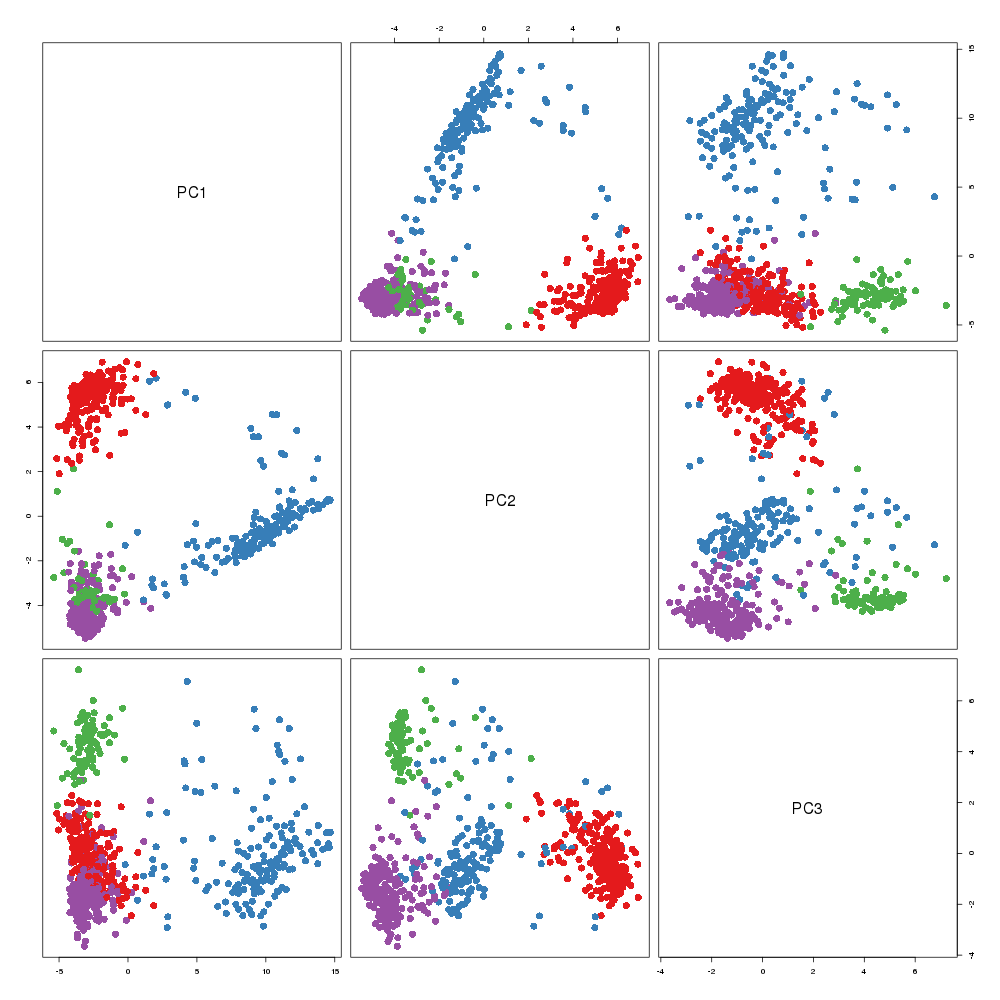

Supplement: Supplementary file 8 — Additional file 8 Pair plots of all the pCA (PBMCs) implementations. [file 13059_2019_1900_MOESM8_ESM.gz › AdditionalFile8/dask_ml.png]

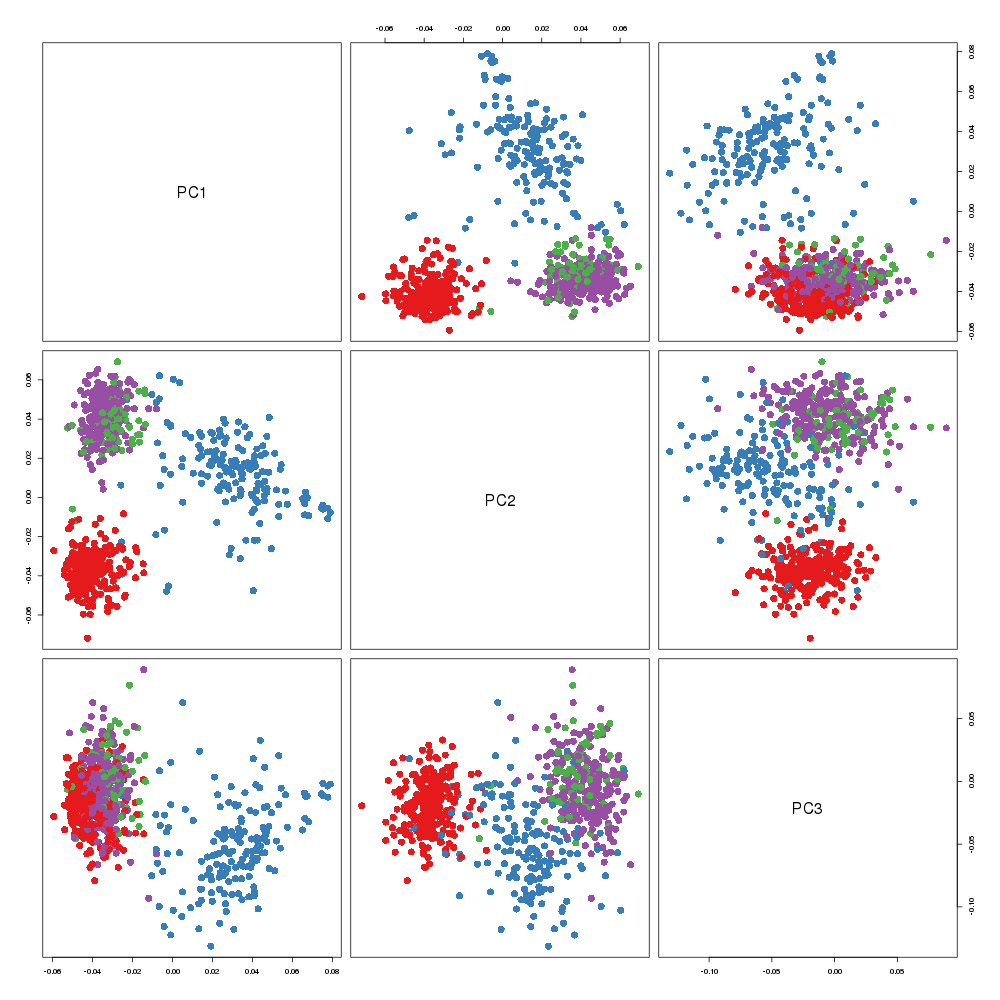

Supplement: Supplementary file 8 — Additional file 8 Pair plots of all the pCA (PBMCs) implementations. [file 13059_2019_1900_MOESM8_ESM.gz › AdditionalFile8/Halko_0iter.png]

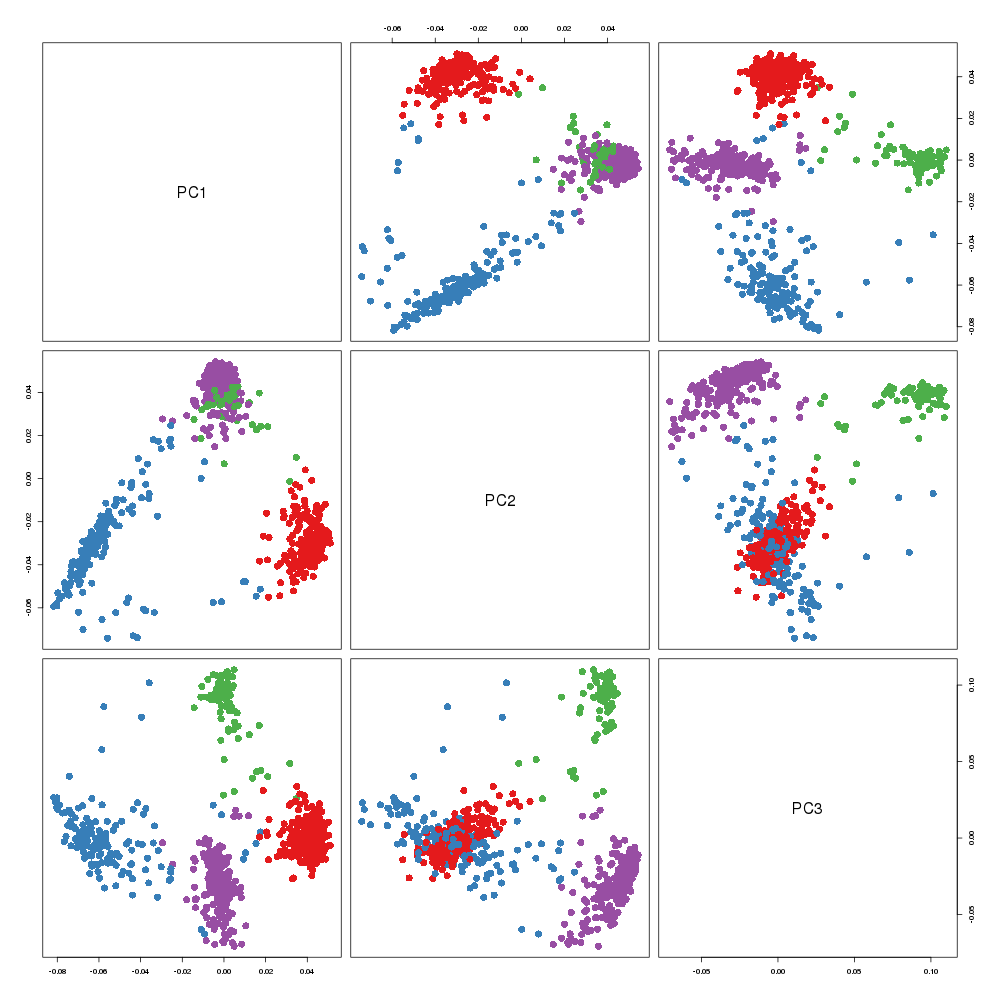

Supplement: Supplementary file 8 — Additional file 8 Pair plots of all the pCA (PBMCs) implementations. [file 13059_2019_1900_MOESM8_ESM.gz › AdditionalFile8/PCA.png]

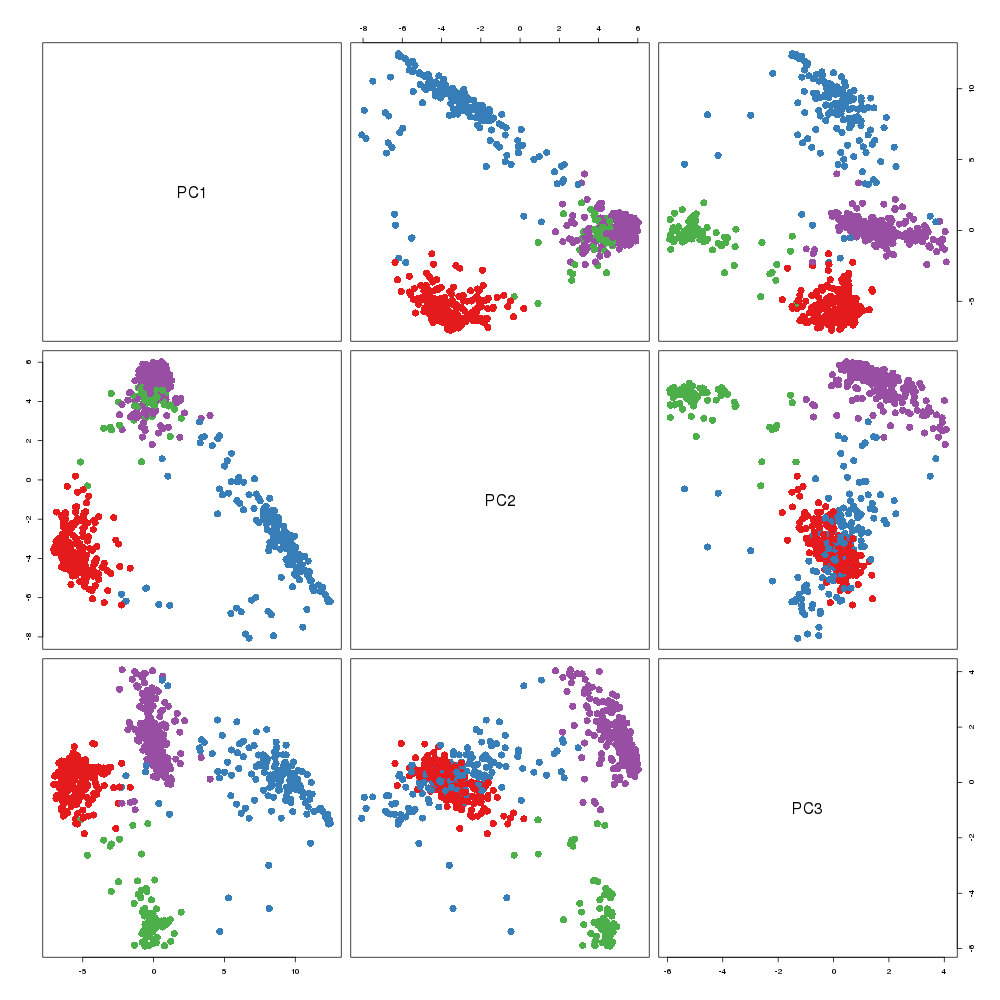

Supplement: Supplementary file 8 — Additional file 8 Pair plots of all the pCA (PBMCs) implementations. [file 13059_2019_1900_MOESM8_ESM.gz › AdditionalFile8/MultivariateStats.png]

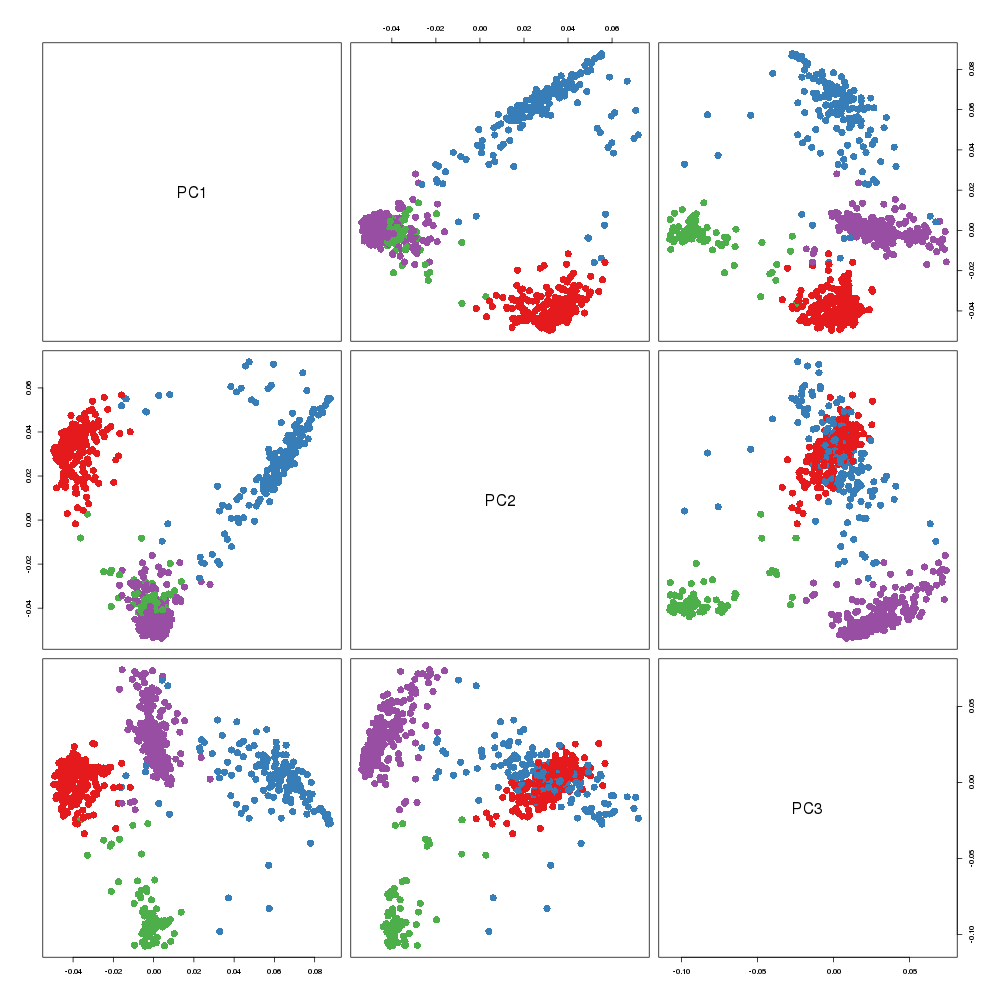

Supplement: Supplementary file 8 — Additional file 8 Pair plots of all the pCA (PBMCs) implementations. [file 13059_2019_1900_MOESM8_ESM.gz › AdditionalFile8/IRLB.png]

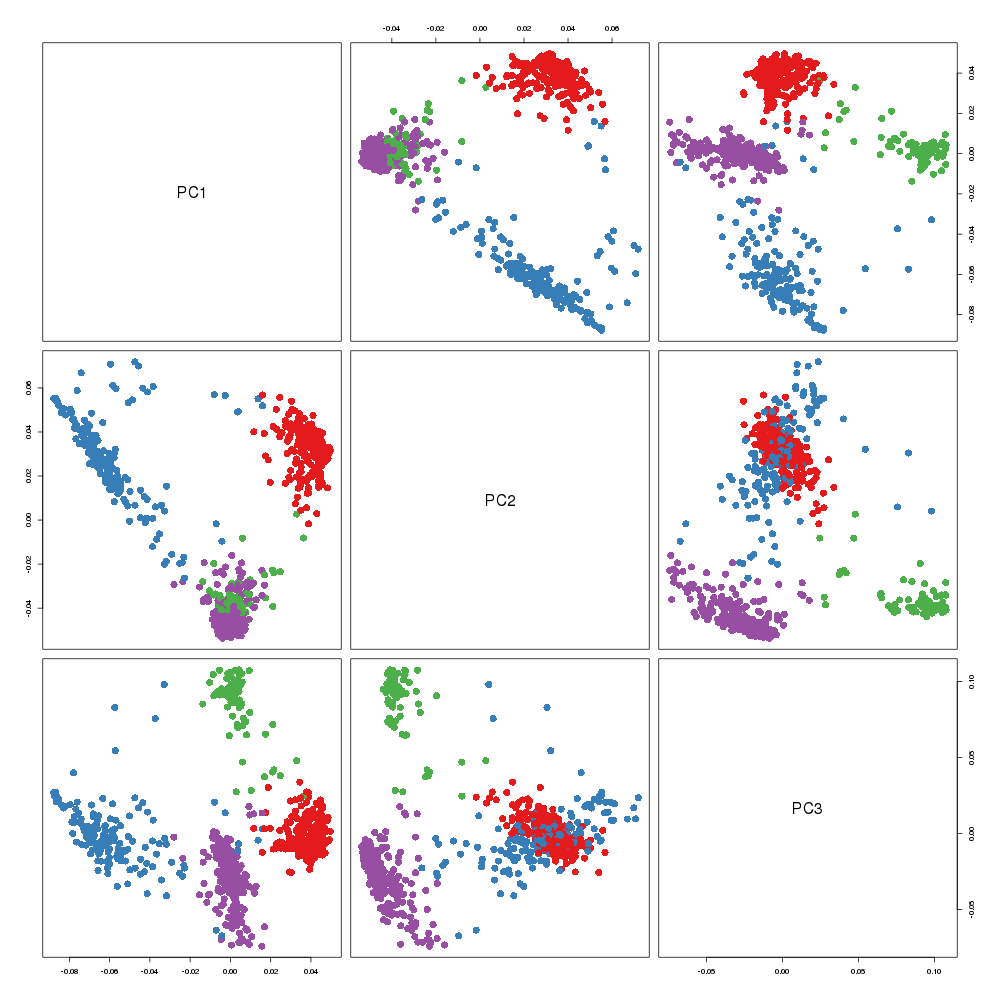

Supplement: Supplementary file 8 — Additional file 8 Pair plots of all the pCA (PBMCs) implementations. [file 13059_2019_1900_MOESM8_ESM.gz › AdditionalFile8/rsvd.png]

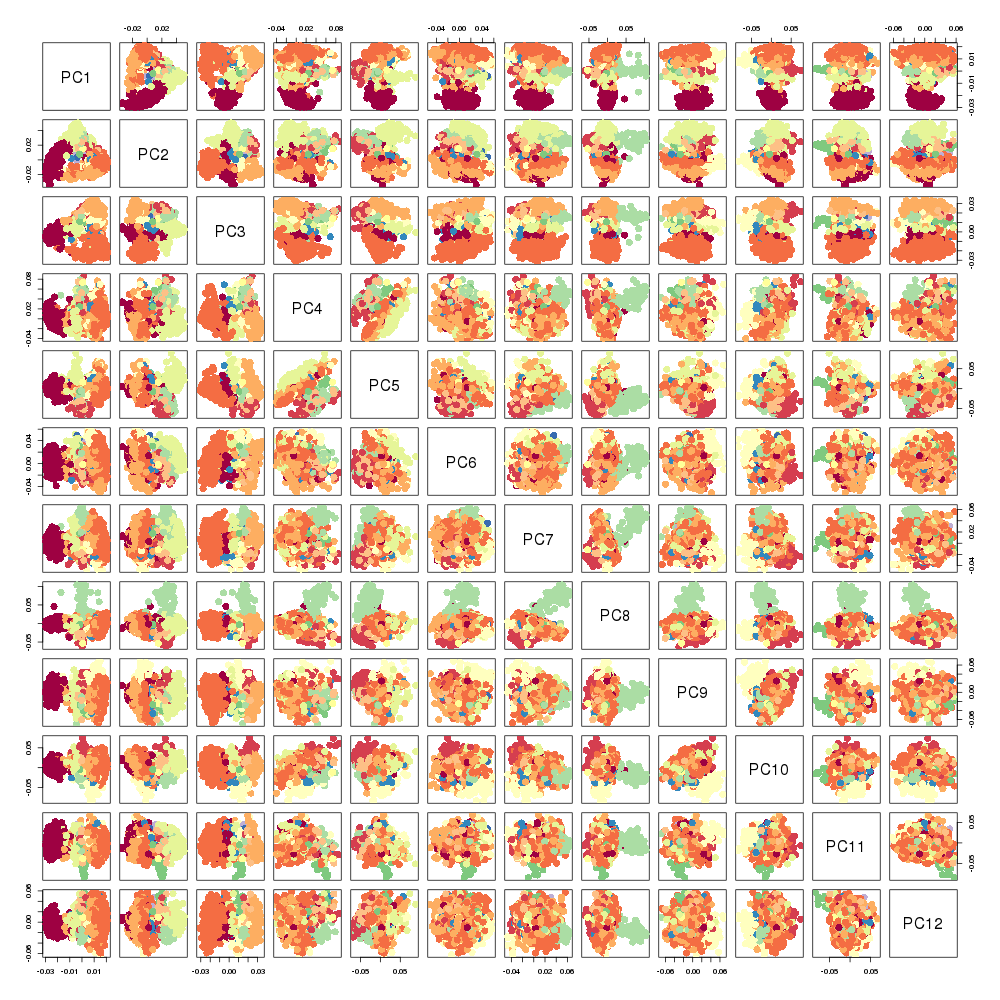

Supplement: Supplementary file 9 — Additional file 9 Pair plots of all the pCA (Pancreas) implementations. [file 13059_2019_1900_MOESM9_ESM.gz › AdditionalFile9/GD_step1000_epoch10.png]

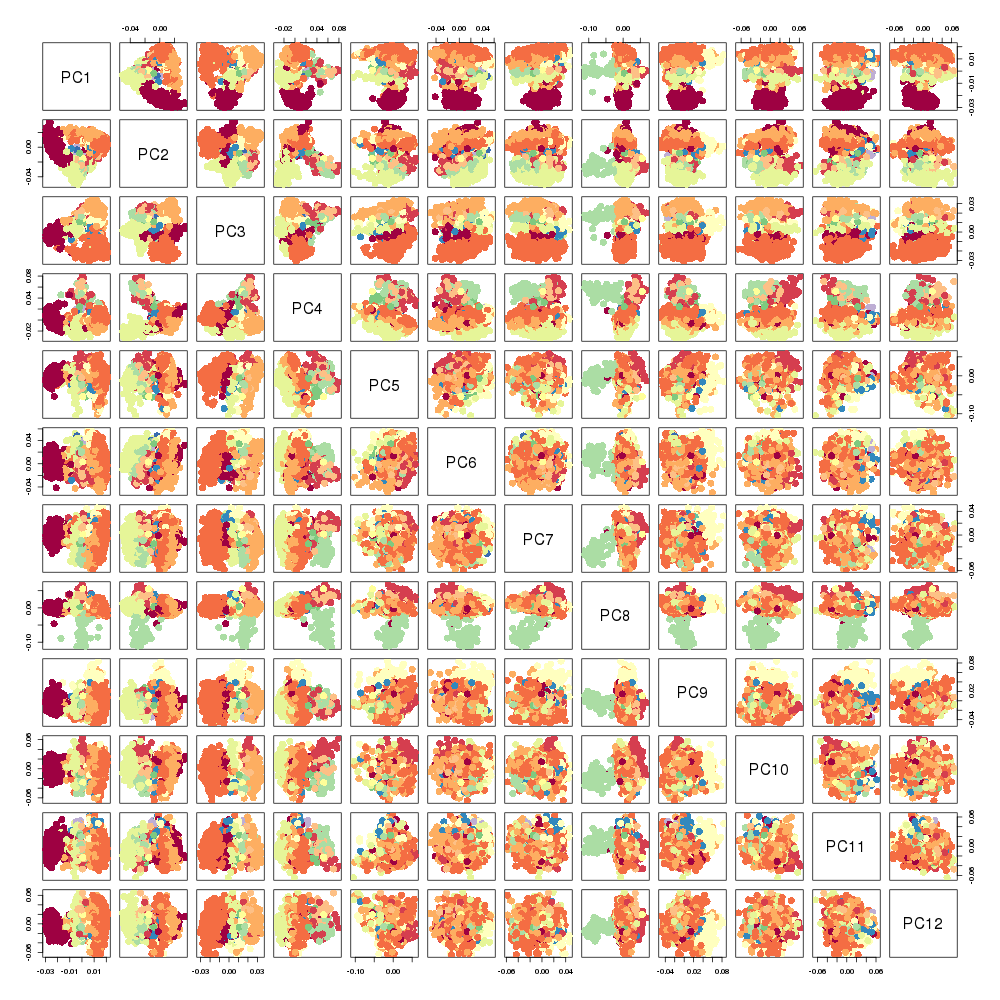

Supplement: Supplementary file 9 — Additional file 9 Pair plots of all the pCA (Pancreas) implementations. [file 13059_2019_1900_MOESM9_ESM.gz › AdditionalFile9/Algorithm971_3iter.png]

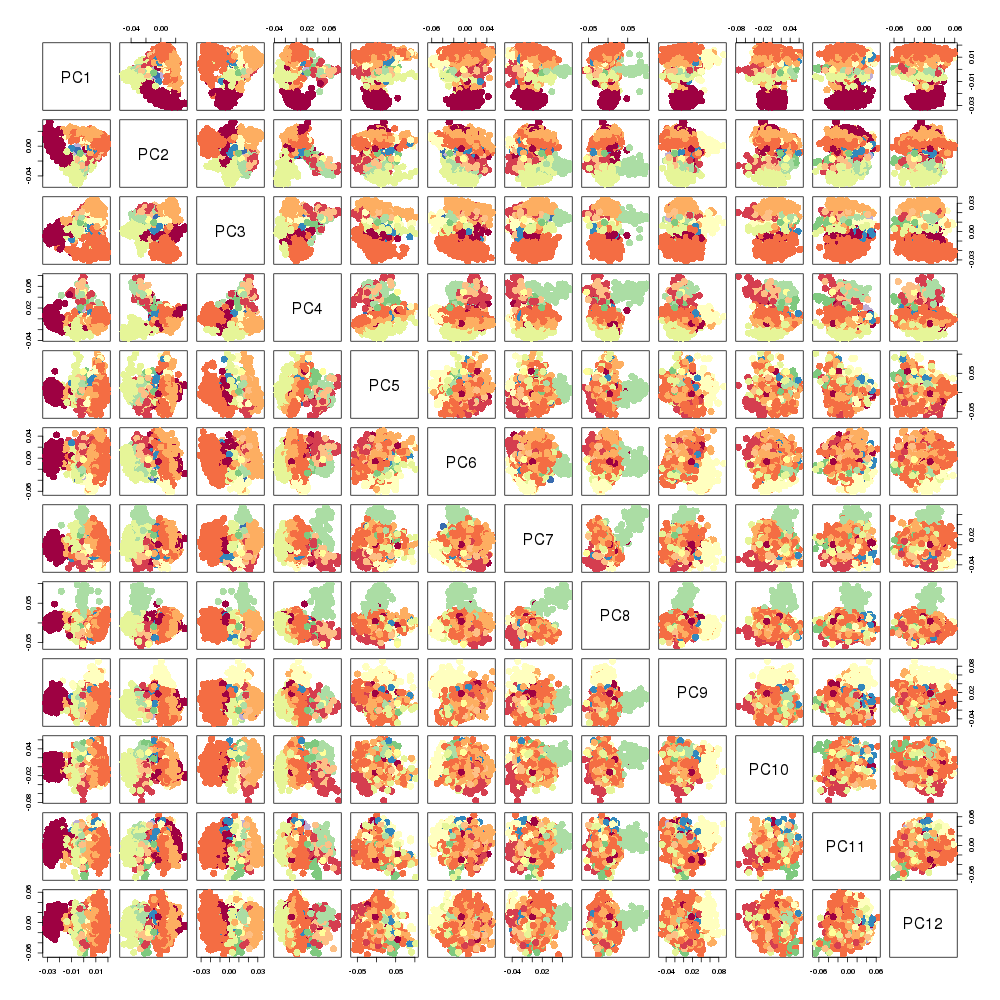

Supplement: Supplementary file 9 — Additional file 9 Pair plots of all the pCA (Pancreas) implementations. [file 13059_2019_1900_MOESM9_ESM.gz › AdditionalFile9/CellRanger.png]

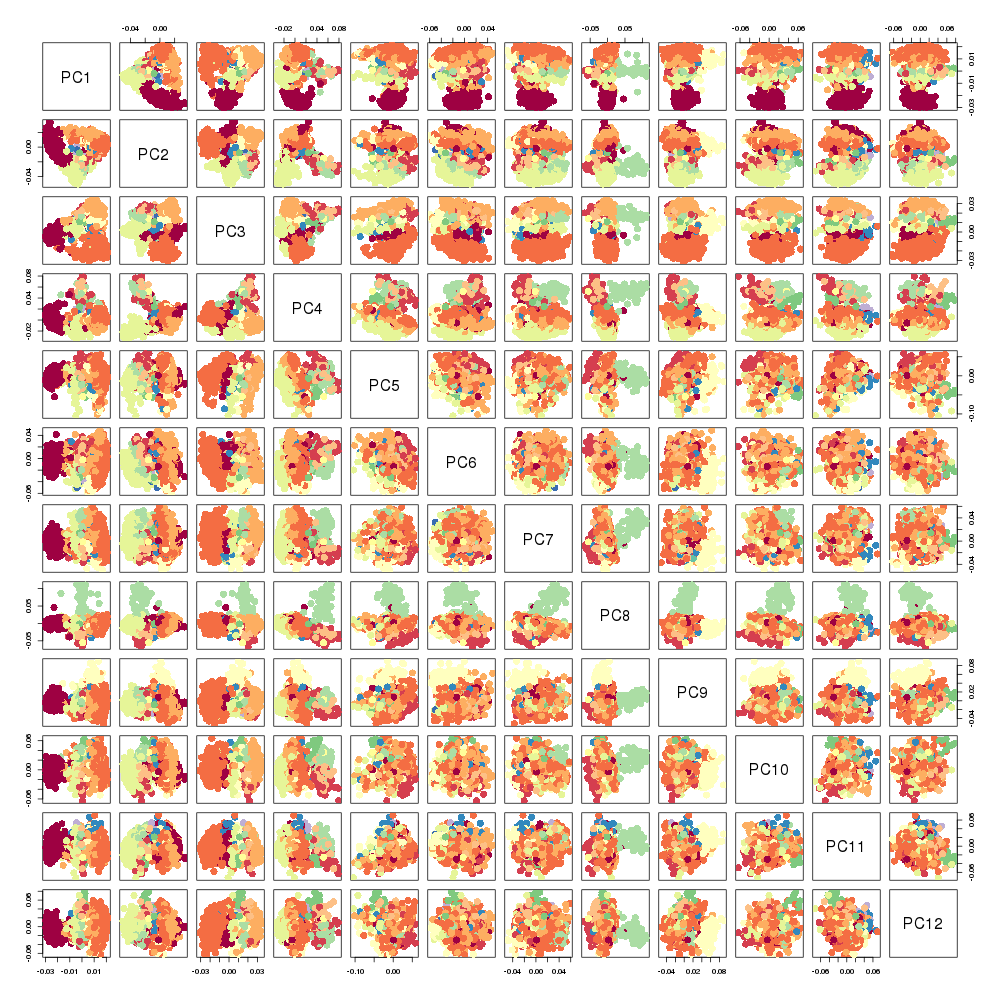

Supplement: Supplementary file 9 — Additional file 9 Pair plots of all the pCA (Pancreas) implementations. [file 13059_2019_1900_MOESM9_ESM.gz › AdditionalFile9/Halko_2iter.png]

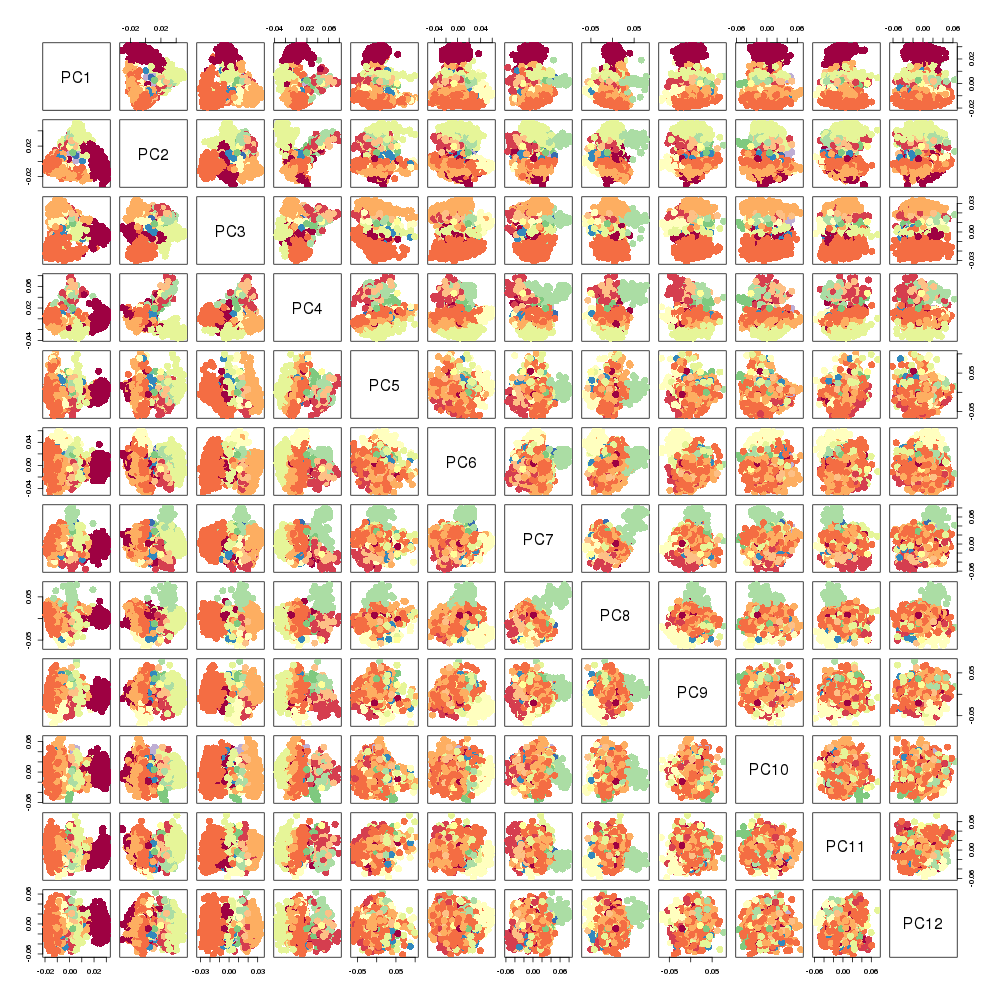

Supplement: Supplementary file 9 — Additional file 9 Pair plots of all the pCA (Pancreas) implementations. [file 13059_2019_1900_MOESM9_ESM.gz › AdditionalFile9/Sklearn_Incremental.png]

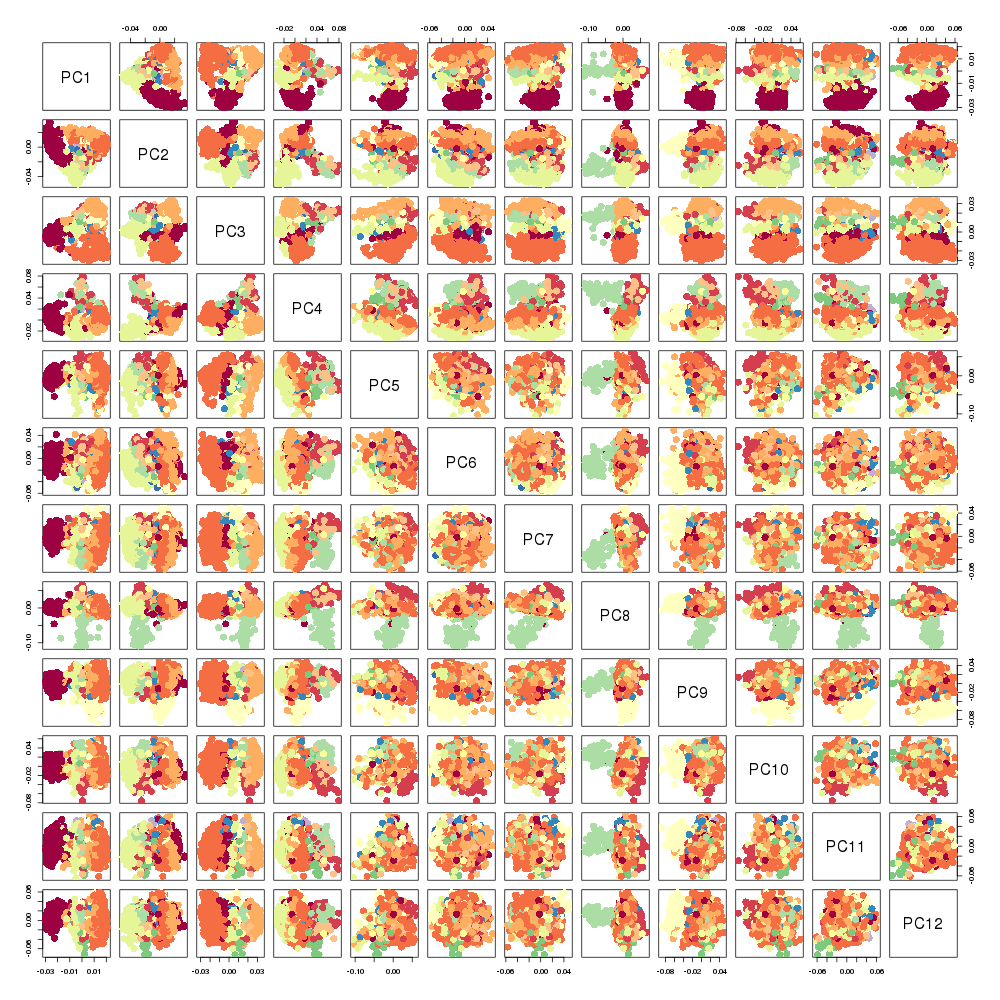

Supplement: Supplementary file 9 — Additional file 9 Pair plots of all the pCA (Pancreas) implementations. [file 13059_2019_1900_MOESM9_ESM.gz › AdditionalFile9/Halko_3iter.png]

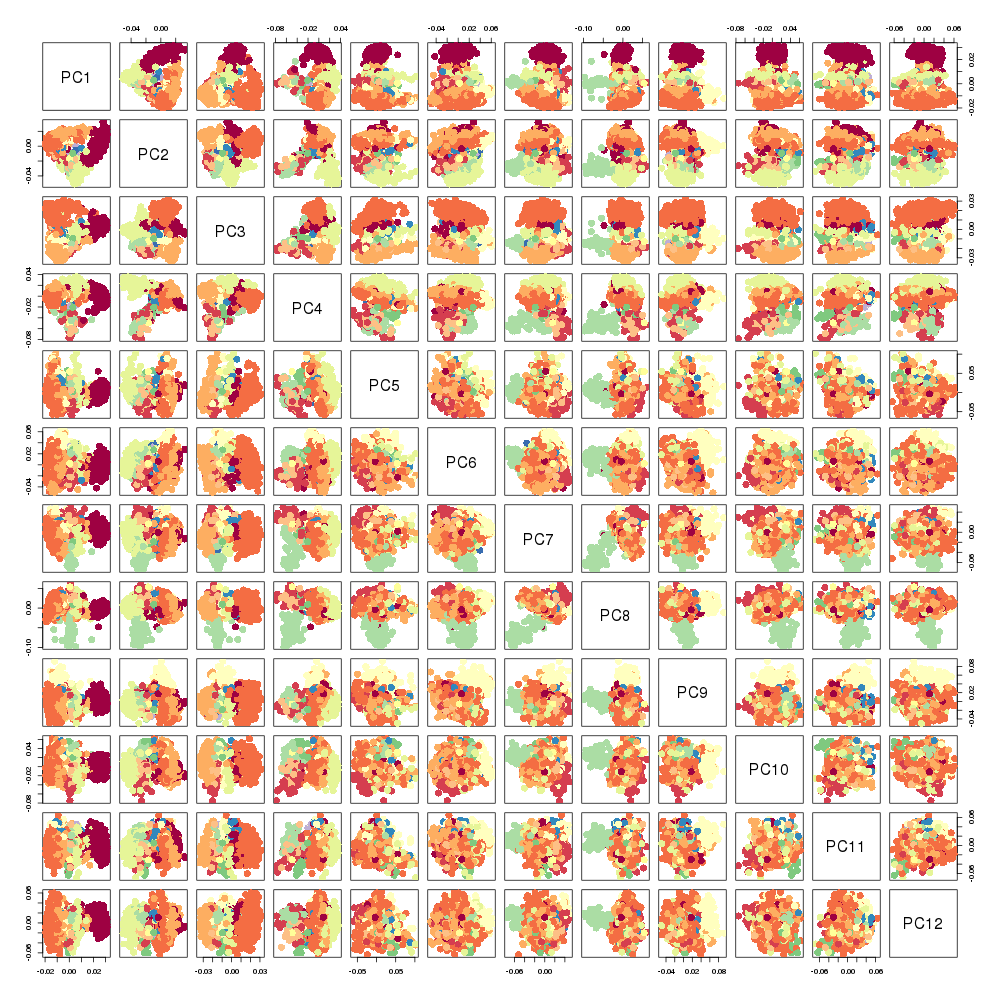

Supplement: Supplementary file 9 — Additional file 9 Pair plots of all the pCA (Pancreas) implementations. [file 13059_2019_1900_MOESM9_ESM.gz › AdditionalFile9/Sklearn_randomized_svd.png]

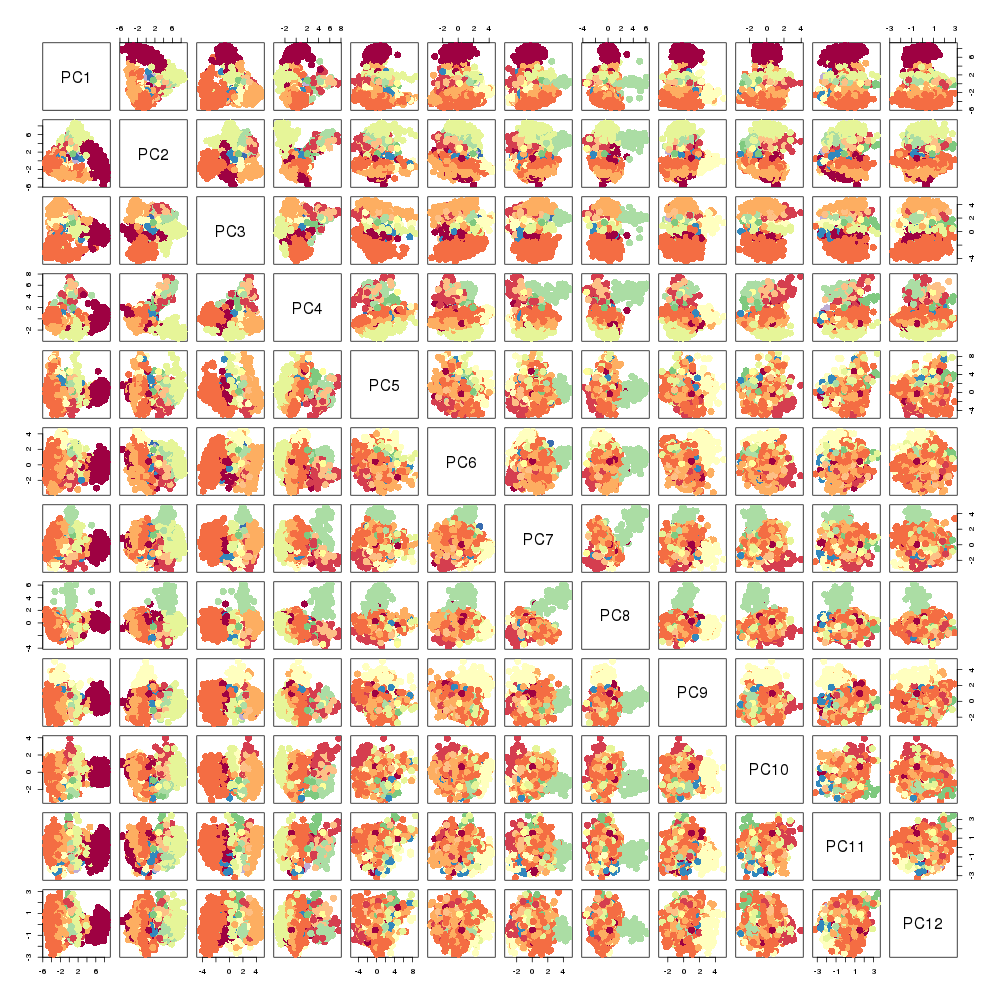

Supplement: Supplementary file 9 — Additional file 9 Pair plots of all the pCA (Pancreas) implementations. [file 13059_2019_1900_MOESM9_ESM.gz › AdditionalFile9/Sklearn_ARPACK.png]

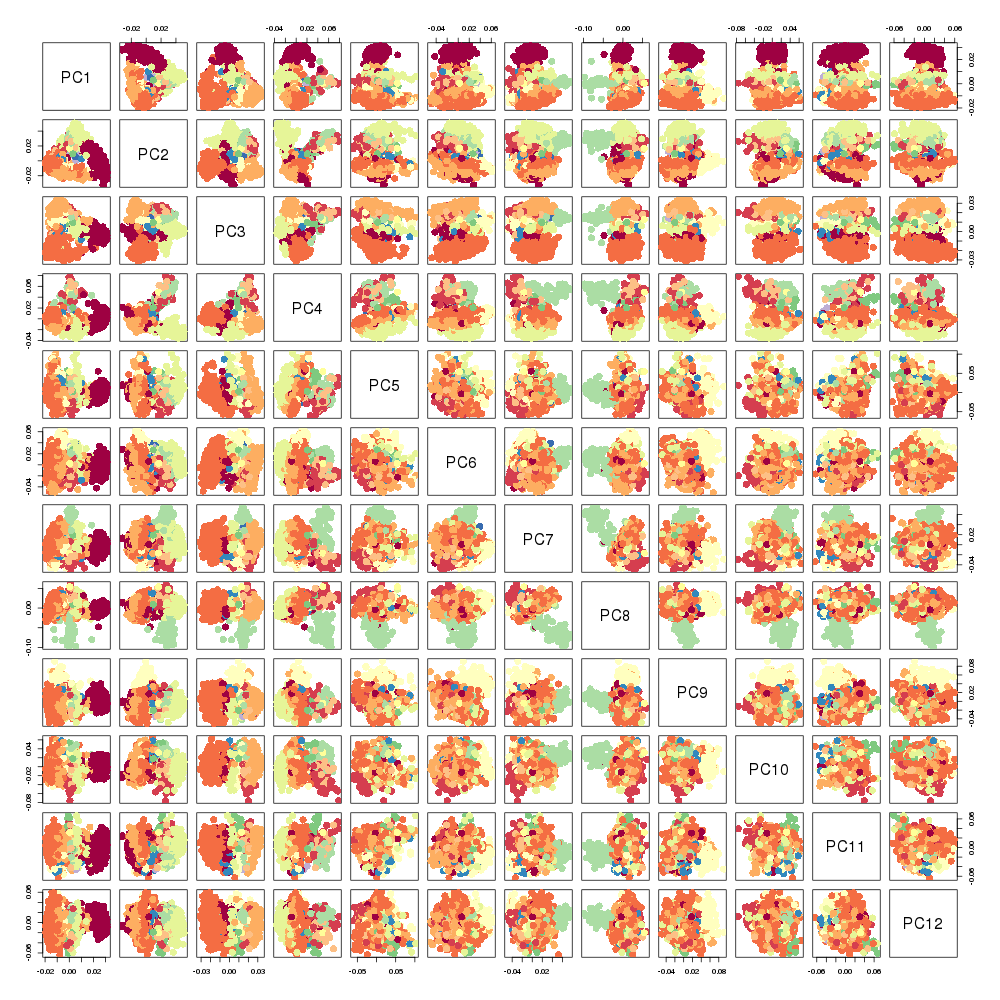

Supplement: Supplementary file 9 — Additional file 9 Pair plots of all the pCA (Pancreas) implementations. [file 13059_2019_1900_MOESM9_ESM.gz › AdditionalFile9/PROPACK.png]

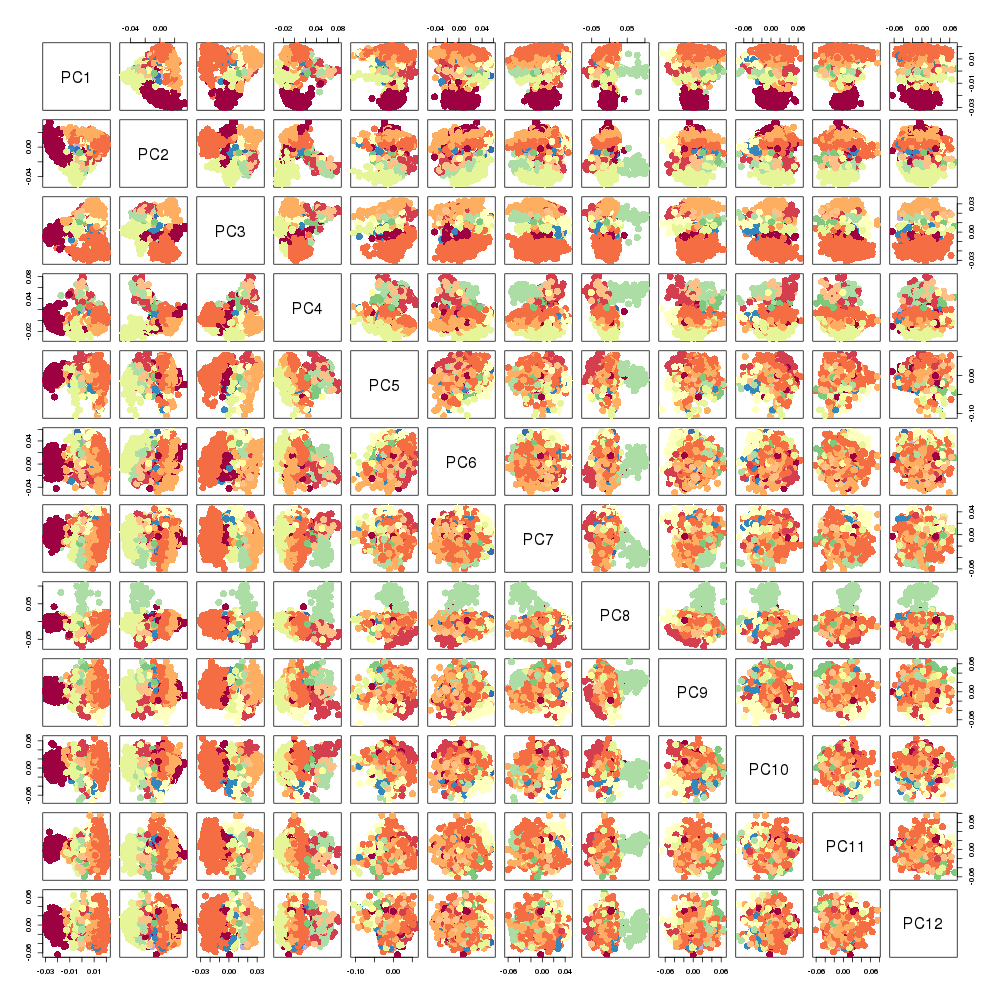

Supplement: Supplementary file 9 — Additional file 9 Pair plots of all the pCA (Pancreas) implementations. [file 13059_2019_1900_MOESM9_ESM.gz › AdditionalFile9/Halko_1iter.png]

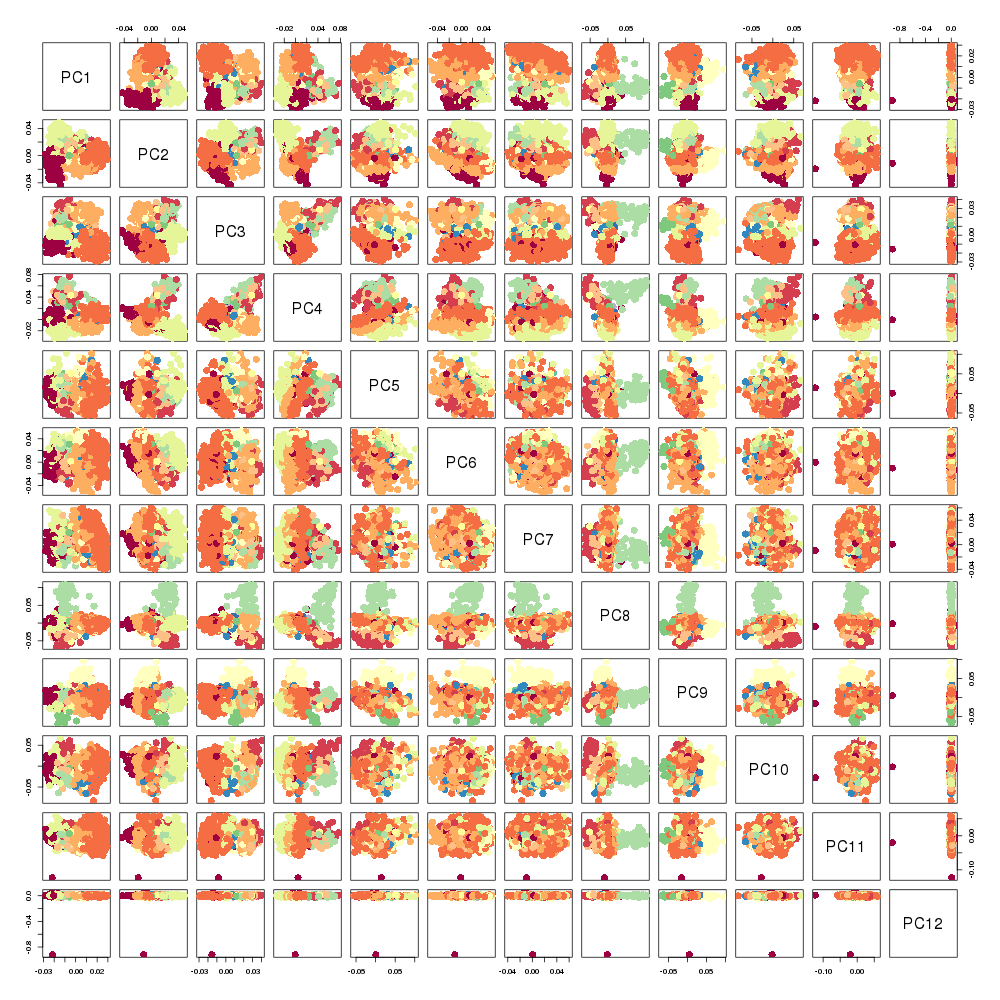

Supplement: Supplementary file 9 — Additional file 9 Pair plots of all the pCA (Pancreas) implementations. [file 13059_2019_1900_MOESM9_ESM.gz › AdditionalFile9/SGD_step100_epoch10.png]

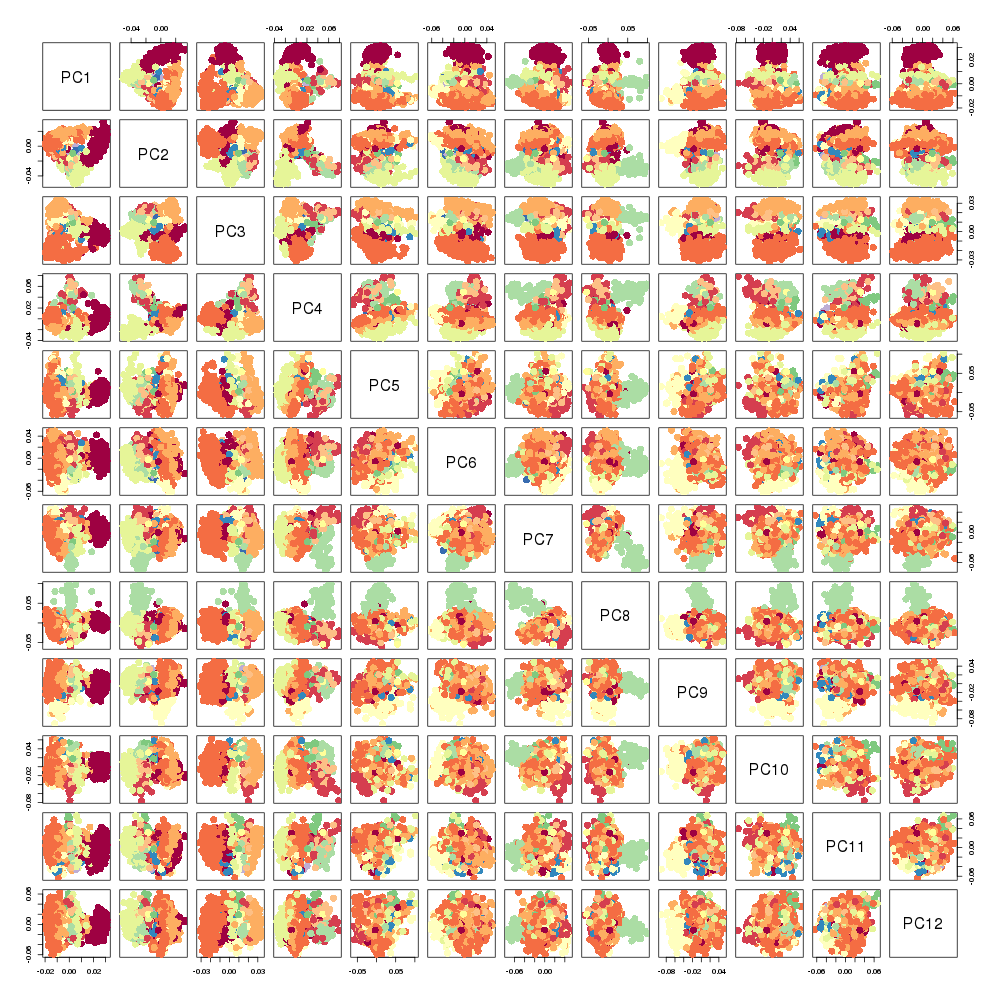

Supplement: Supplementary file 9 — Additional file 9 Pair plots of all the pCA (Pancreas) implementations. [file 13059_2019_1900_MOESM9_ESM.gz › AdditionalFile9/RSpectra.png]

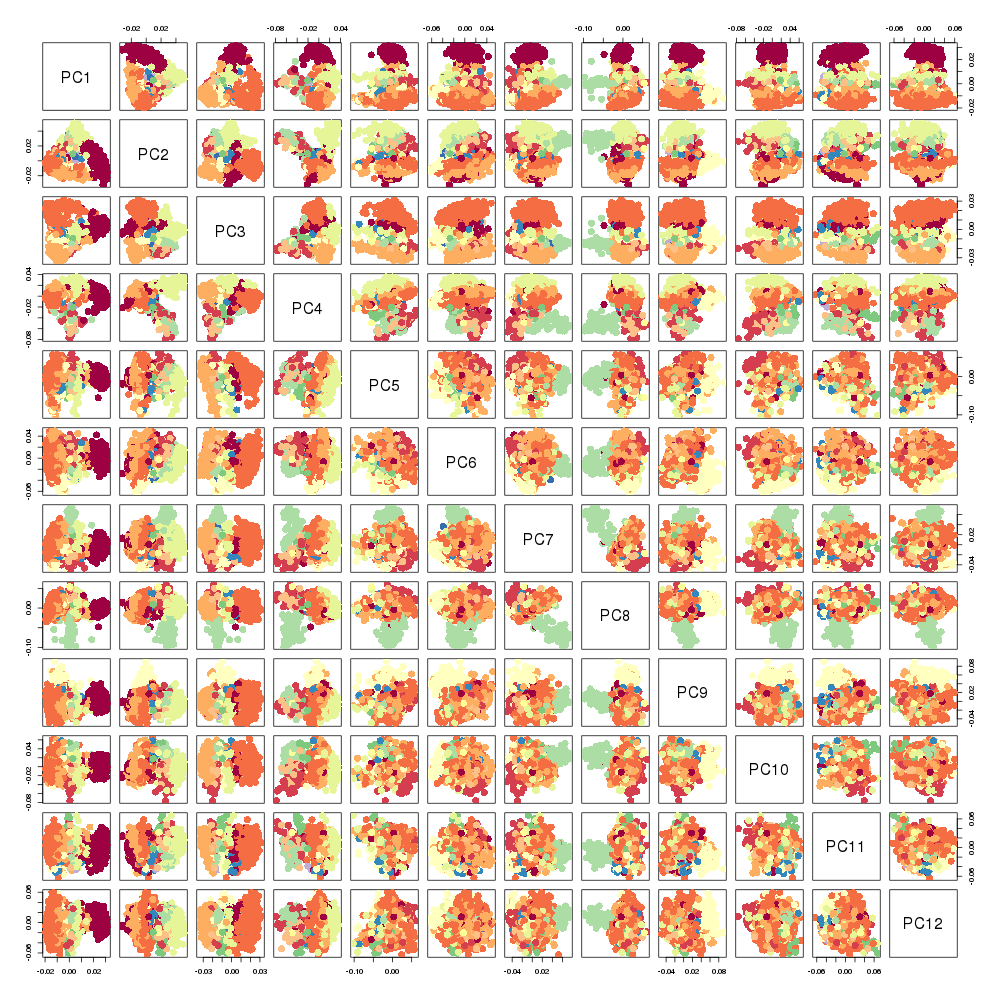

Supplement: Supplementary file 9 — Additional file 9 Pair plots of all the pCA (Pancreas) implementations. [file 13059_2019_1900_MOESM9_ESM.gz › AdditionalFile9/Arpack.jl.png]

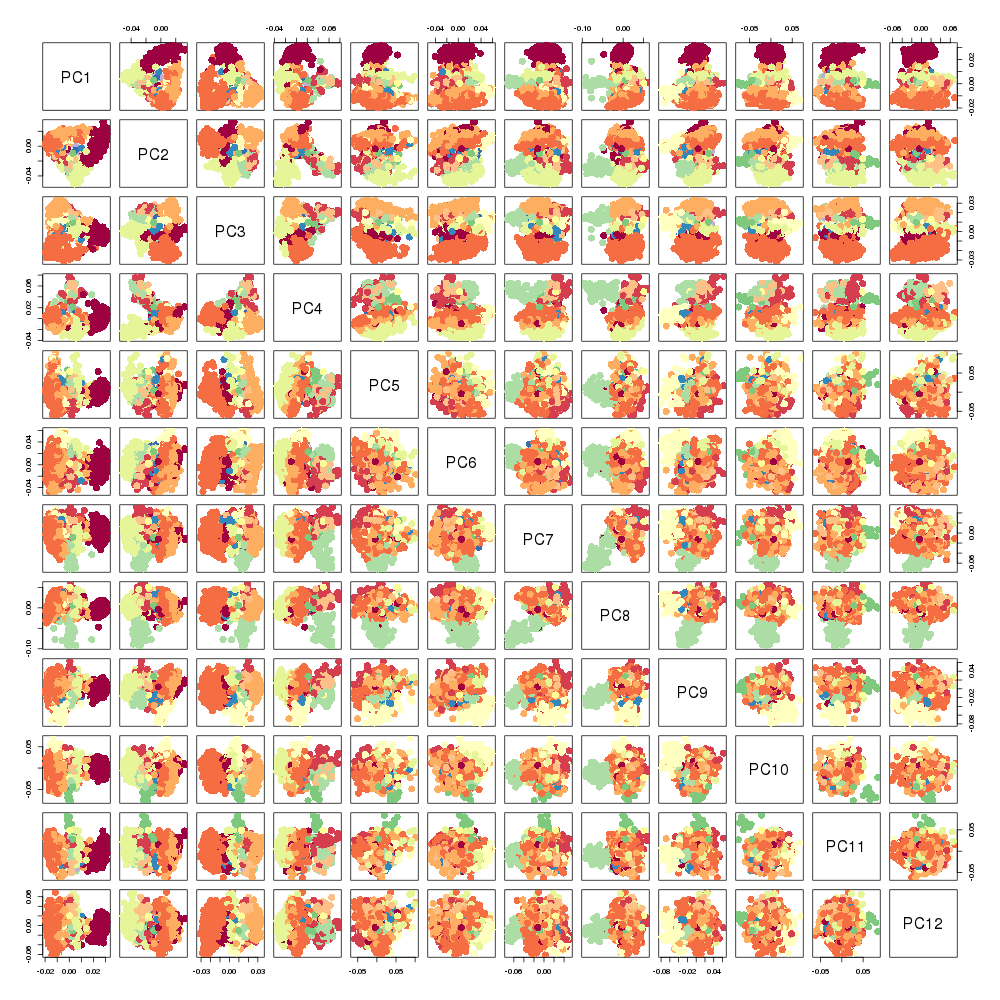

Supplement: Supplementary file 9 — Additional file 9 Pair plots of all the pCA (Pancreas) implementations. [file 13059_2019_1900_MOESM9_ESM.gz › AdditionalFile9/oocRPCA.png]

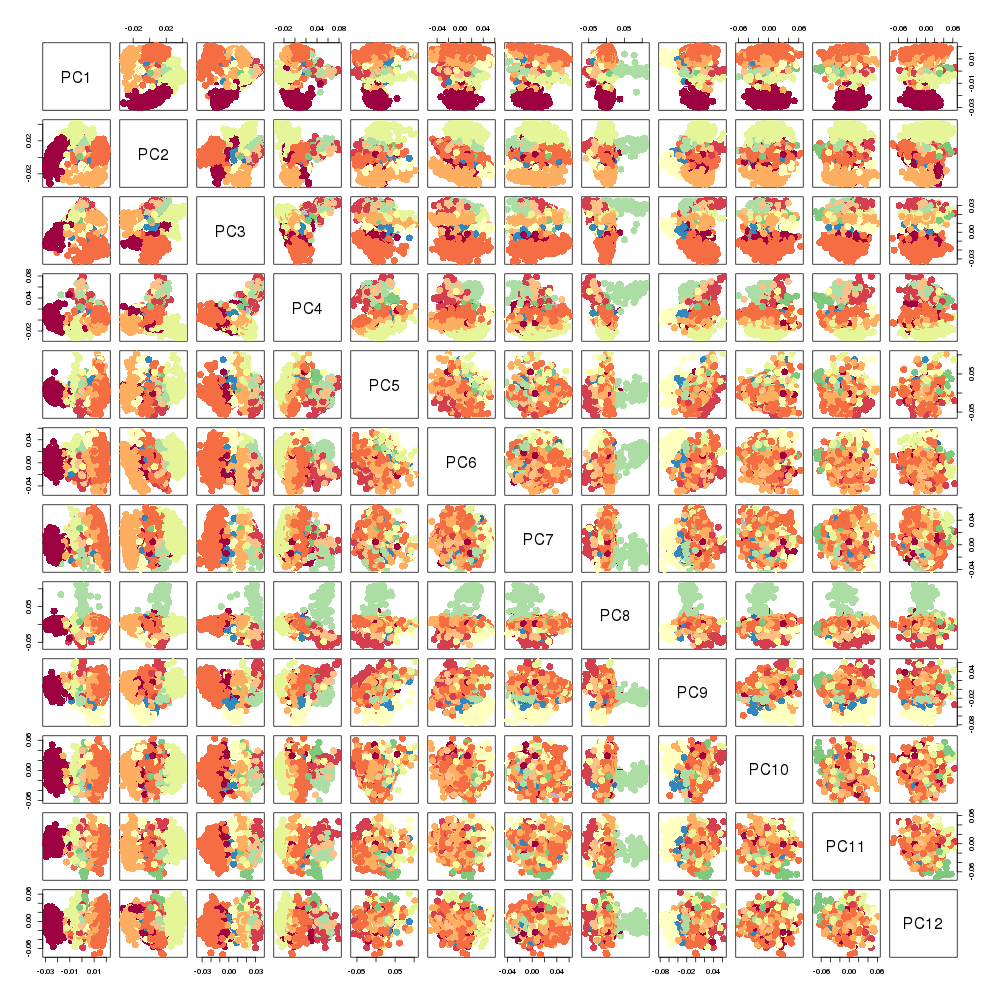

Supplement: Supplementary file 9 — Additional file 9 Pair plots of all the pCA (Pancreas) implementations. [file 13059_2019_1900_MOESM9_ESM.gz › AdditionalFile9/OrthIter.png]

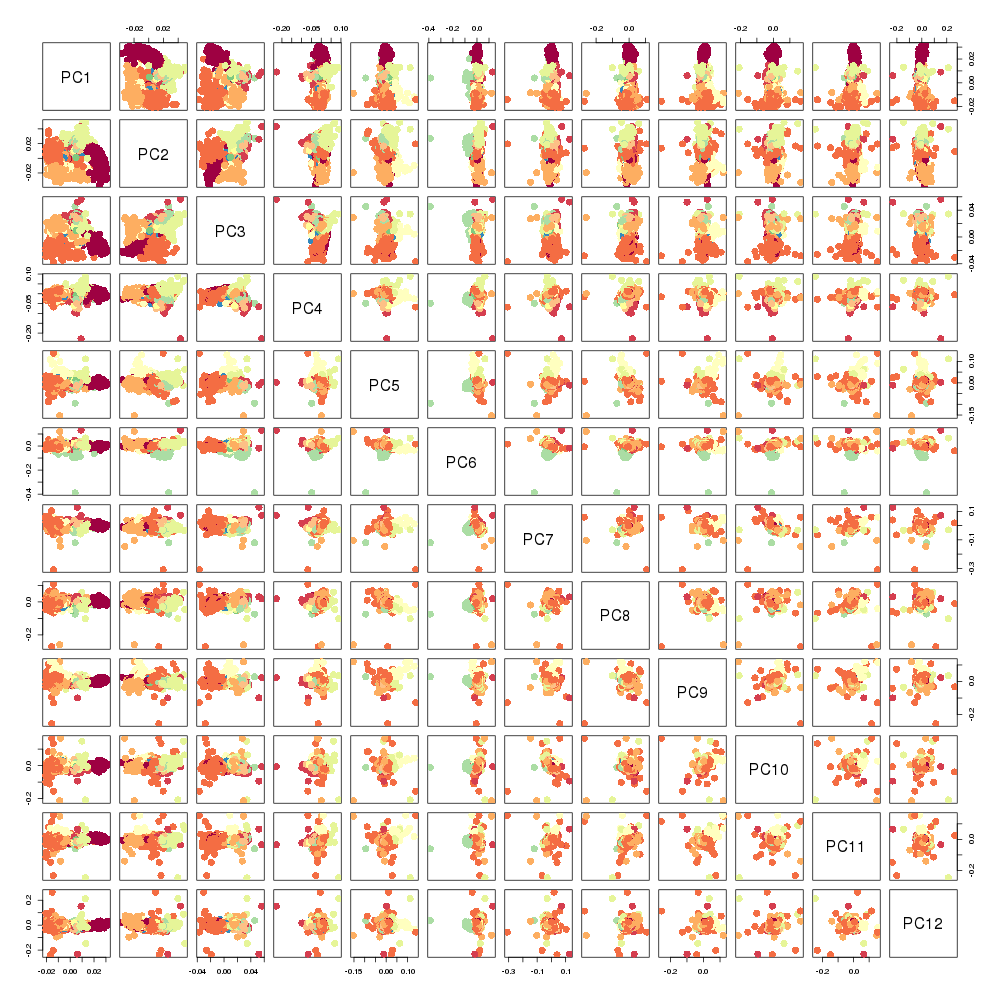

Supplement: Supplementary file 9 — Additional file 9 Pair plots of all the pCA (Pancreas) implementations. [file 13059_2019_1900_MOESM9_ESM.gz › AdditionalFile9/Downsampling.png]

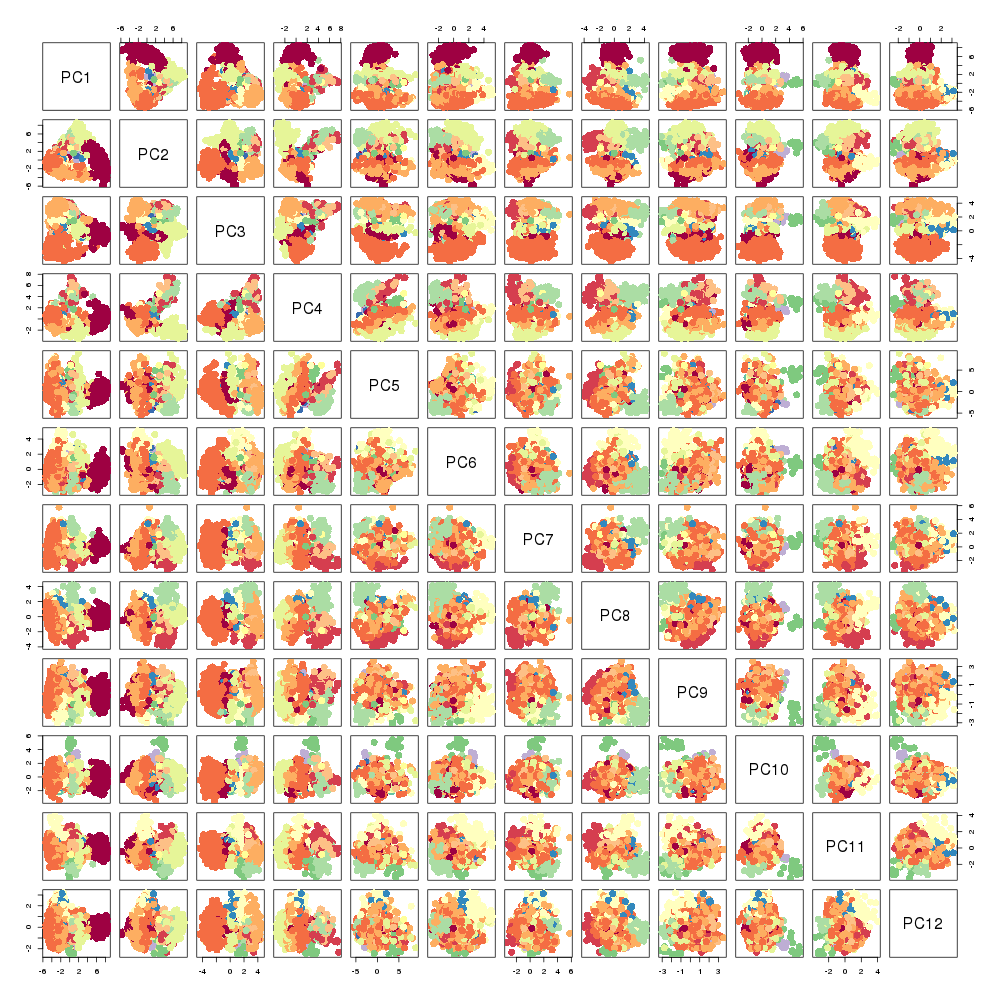

Supplement: Supplementary file 9 — Additional file 9 Pair plots of all the pCA (Pancreas) implementations. [file 13059_2019_1900_MOESM9_ESM.gz › AdditionalFile9/dask_ml.png]

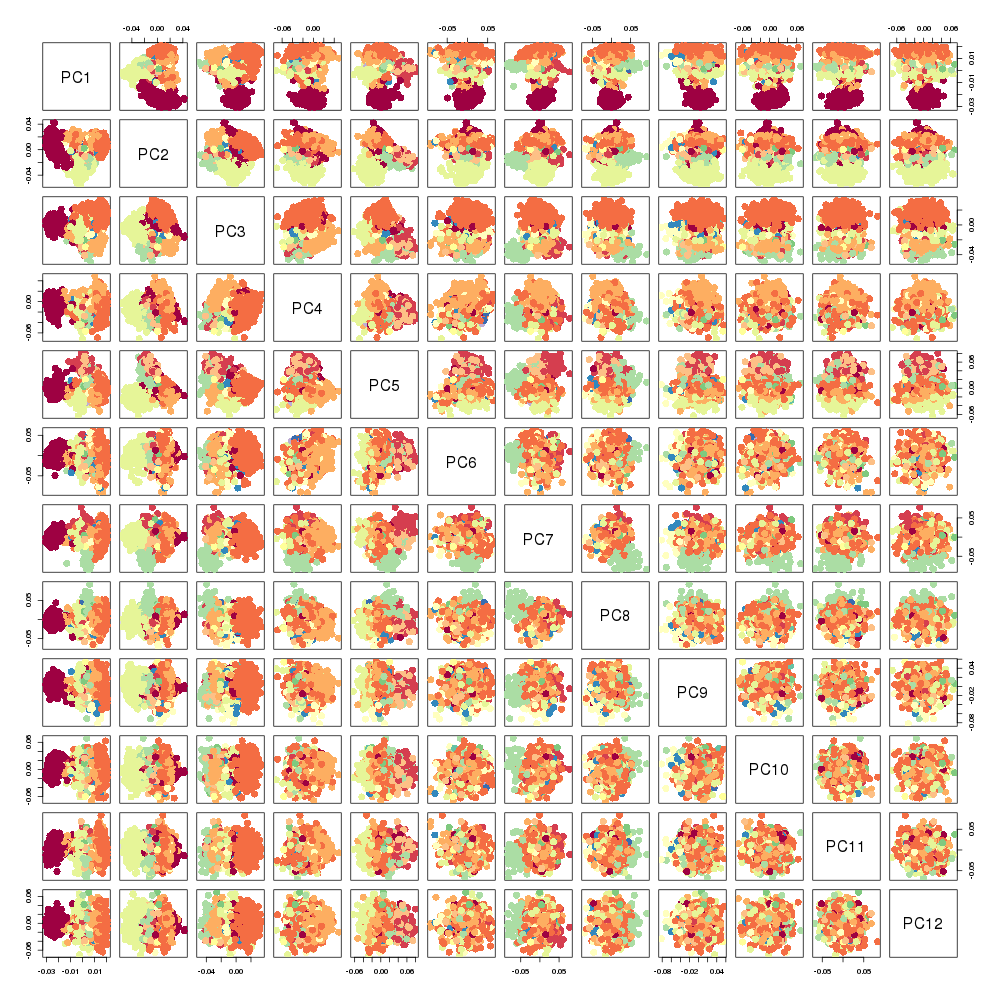

Supplement: Supplementary file 9 — Additional file 9 Pair plots of all the pCA (Pancreas) implementations. [file 13059_2019_1900_MOESM9_ESM.gz › AdditionalFile9/Halko_0iter.png]

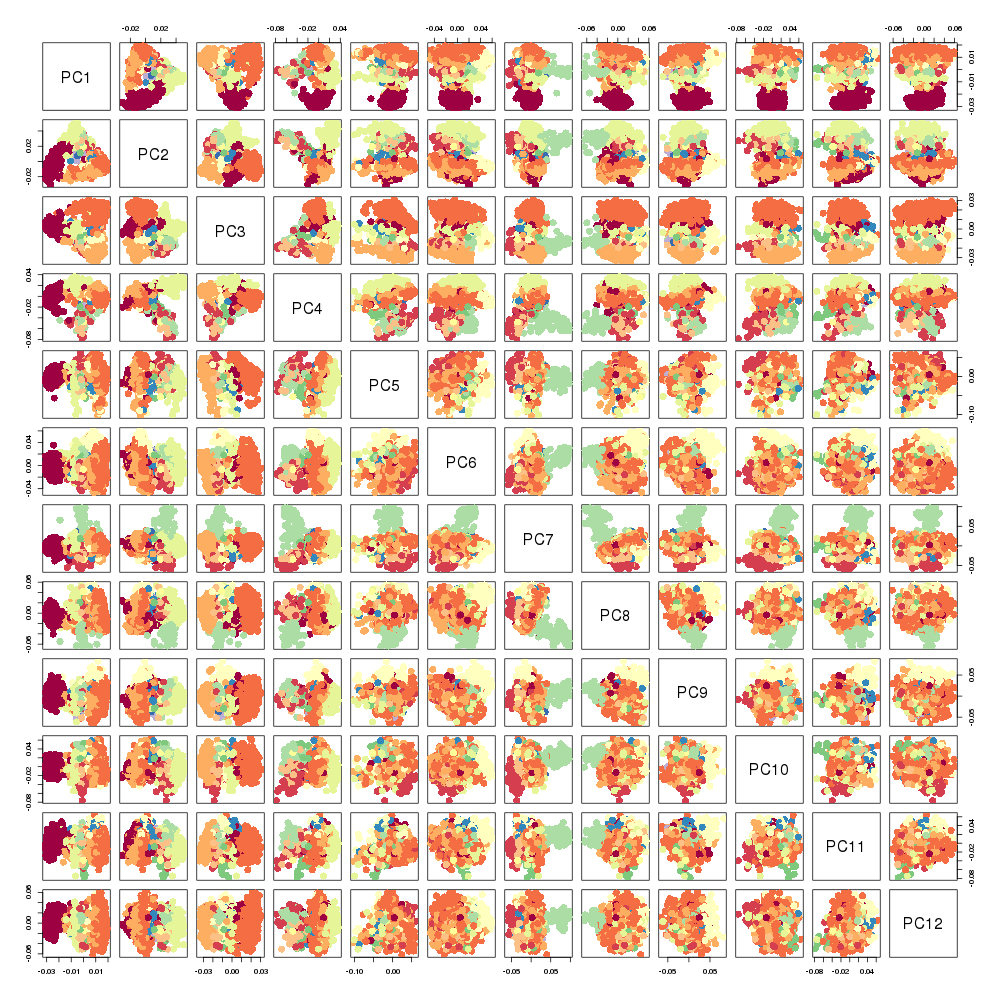

Supplement: Supplementary file 9 — Additional file 9 Pair plots of all the pCA (Pancreas) implementations. [file 13059_2019_1900_MOESM9_ESM.gz › AdditionalFile9/PCA.png]

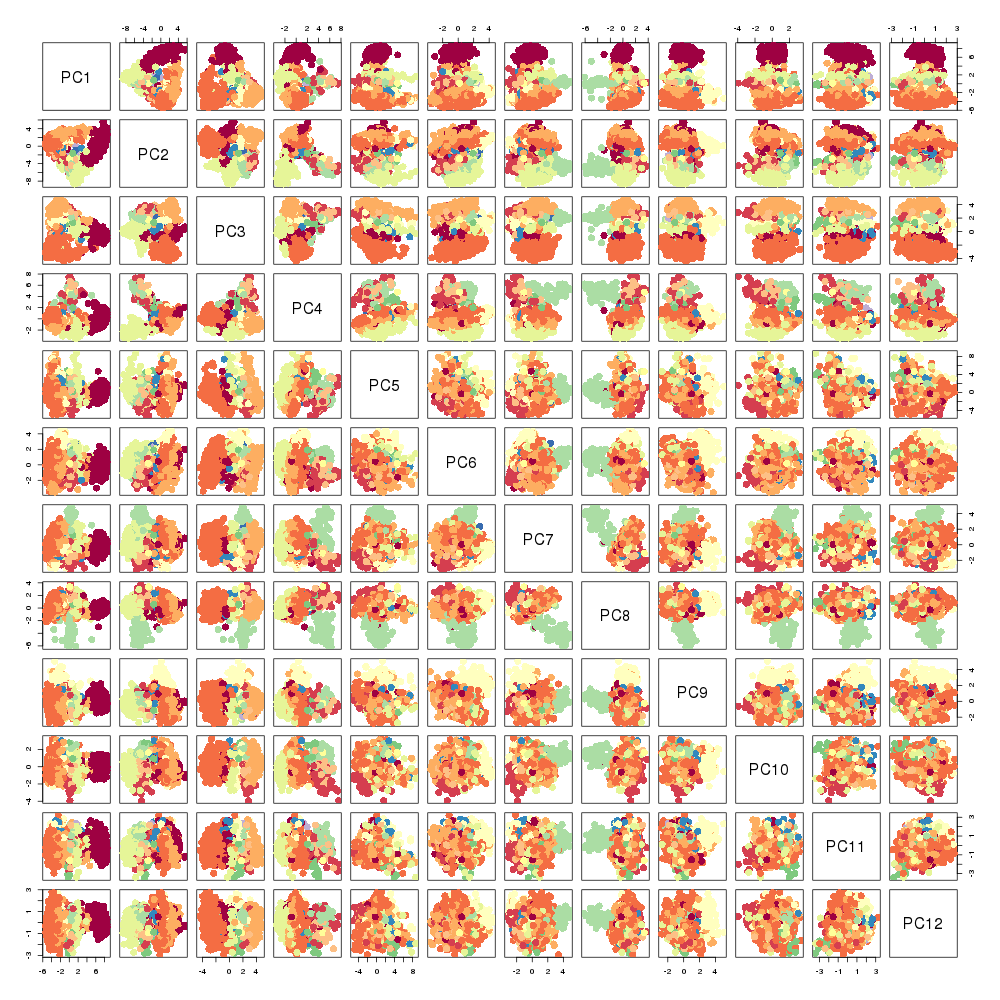

Supplement: Supplementary file 9 — Additional file 9 Pair plots of all the pCA (Pancreas) implementations. [file 13059_2019_1900_MOESM9_ESM.gz › AdditionalFile9/MultivariateStats.png]

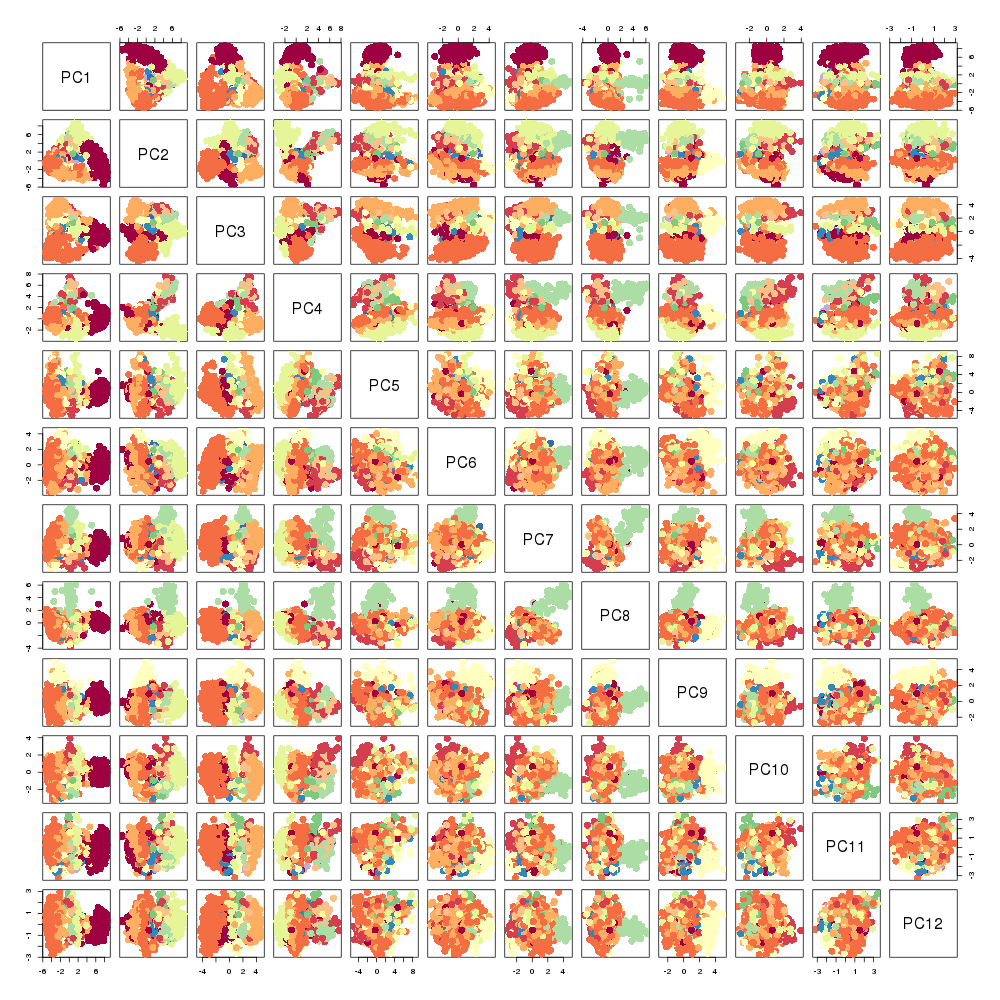

Supplement: Supplementary file 9 — Additional file 9 Pair plots of all the pCA (Pancreas) implementations. [file 13059_2019_1900_MOESM9_ESM.gz › AdditionalFile9/Sklearn_RandomizedPCA.png]

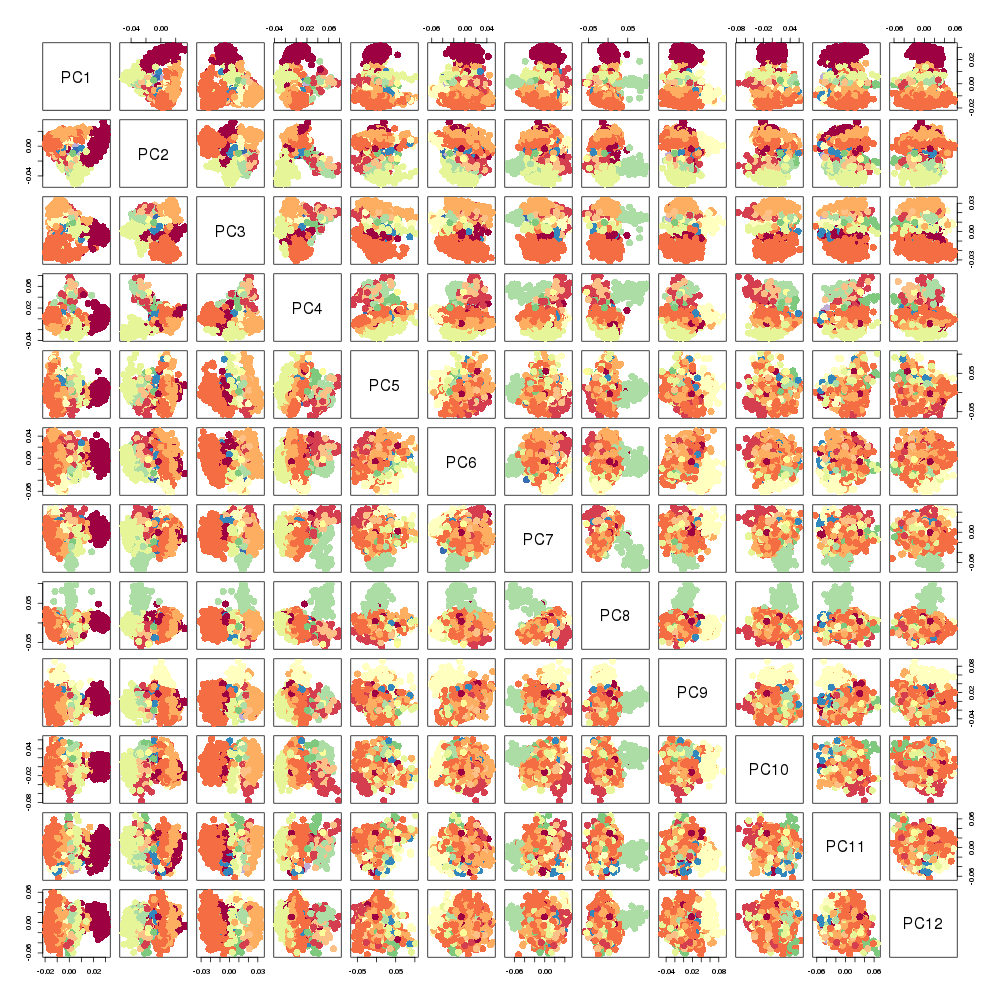

Supplement: Supplementary file 9 — Additional file 9 Pair plots of all the pCA (Pancreas) implementations. [file 13059_2019_1900_MOESM9_ESM.gz › AdditionalFile9/IRLB.png]

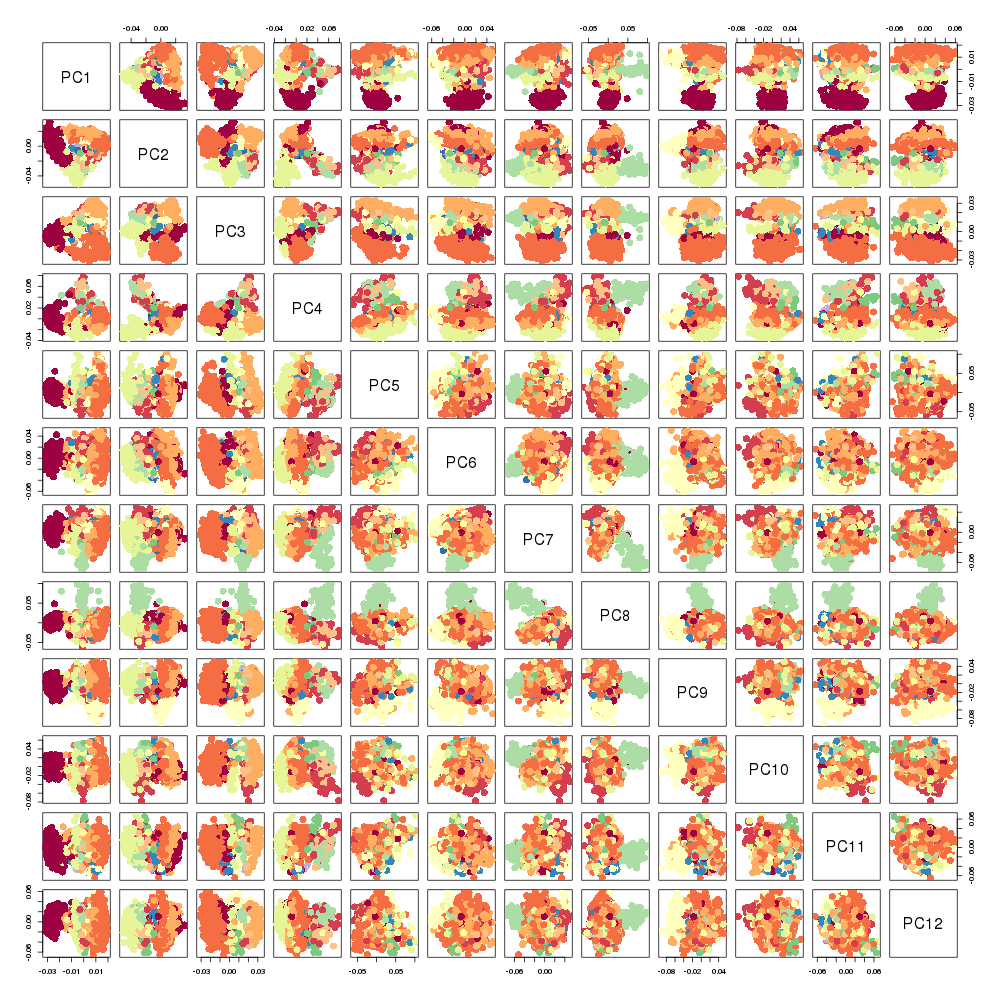

Supplement: Supplementary file 9 — Additional file 9 Pair plots of all the pCA (Pancreas) implementations. [file 13059_2019_1900_MOESM9_ESM.gz › AdditionalFile9/rsvd.png]

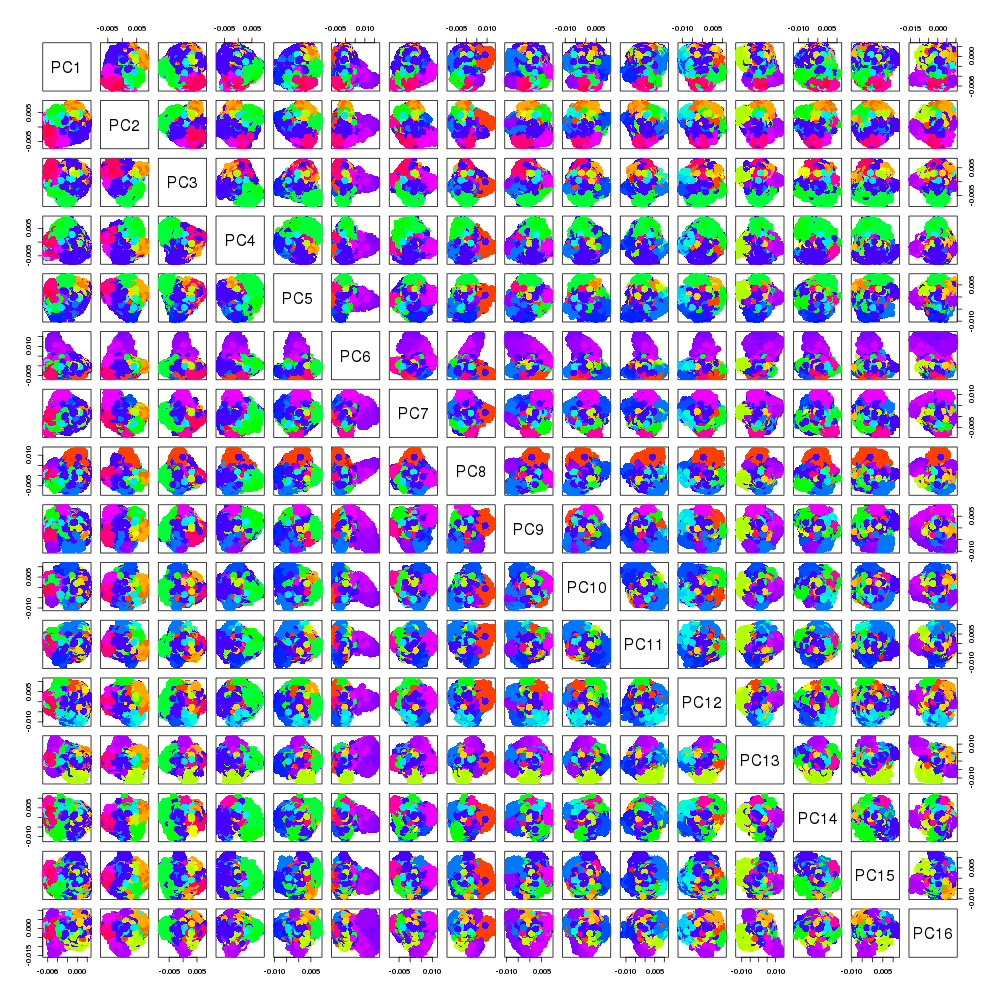

Supplement: Supplementary file 10 — Additional file 10 Pair plots of all the pCA (BrainSpinalCord) implementations. [file 13059_2019_1900_MOESM10_ESM.gz › AdditionalFile10/GD_step1000_epoch10.png]

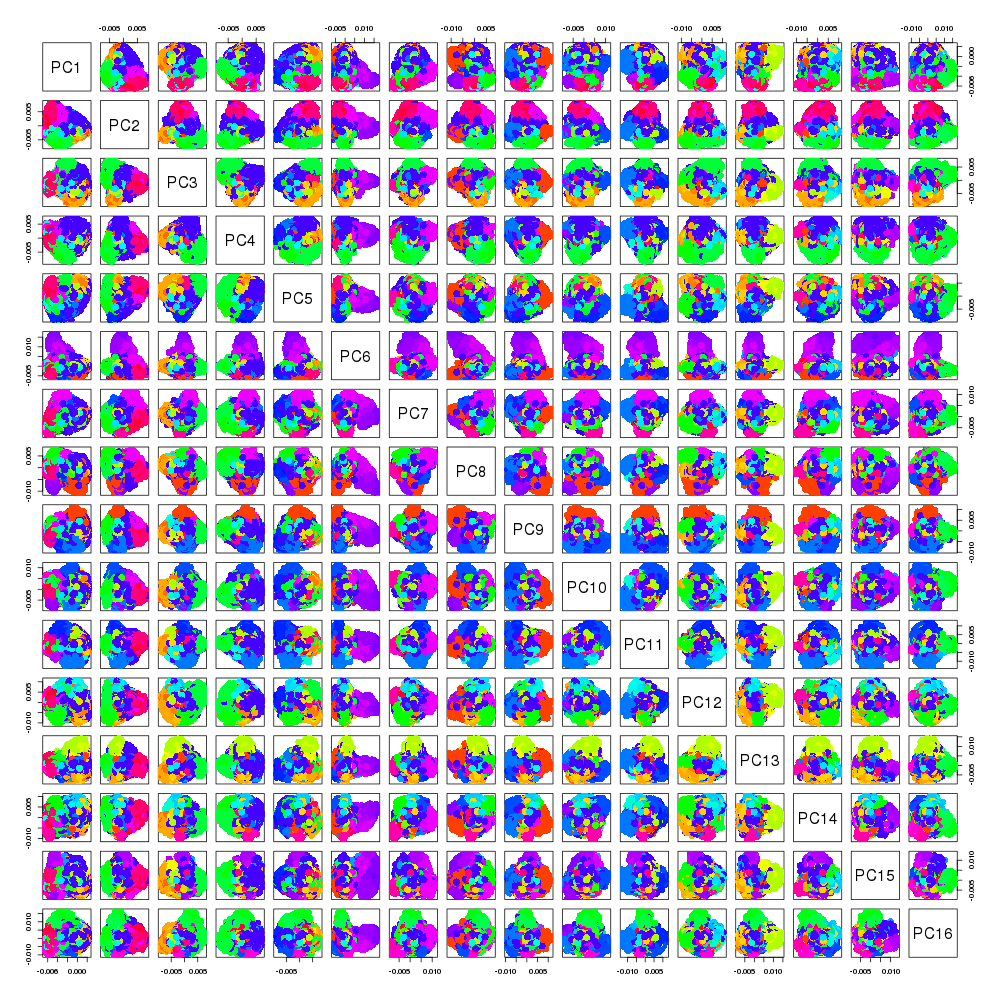

Supplement: Supplementary file 10 — Additional file 10 Pair plots of all the pCA (BrainSpinalCord) implementations. [file 13059_2019_1900_MOESM10_ESM.gz › AdditionalFile10/Algorithm971_3iter.png]

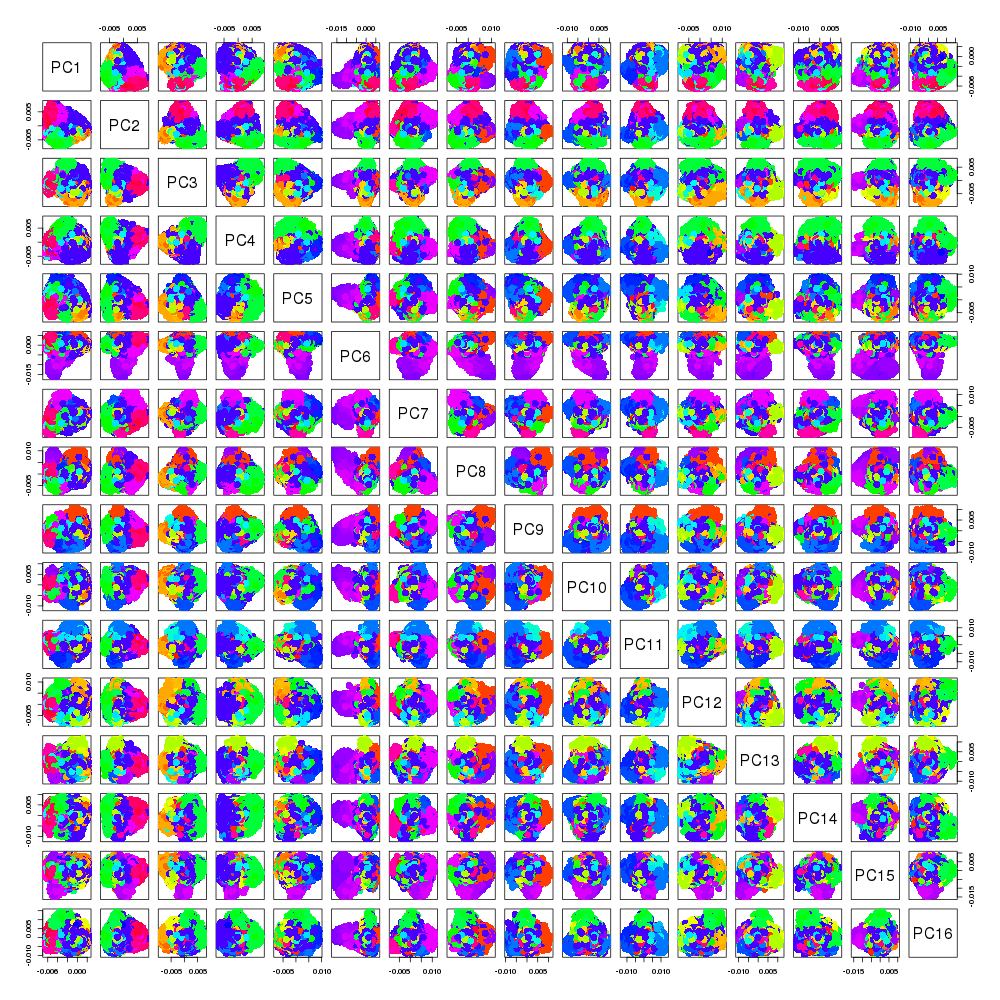

Supplement: Supplementary file 10 — Additional file 10 Pair plots of all the pCA (BrainSpinalCord) implementations. [file 13059_2019_1900_MOESM10_ESM.gz › AdditionalFile10/Halko_2iter.png]

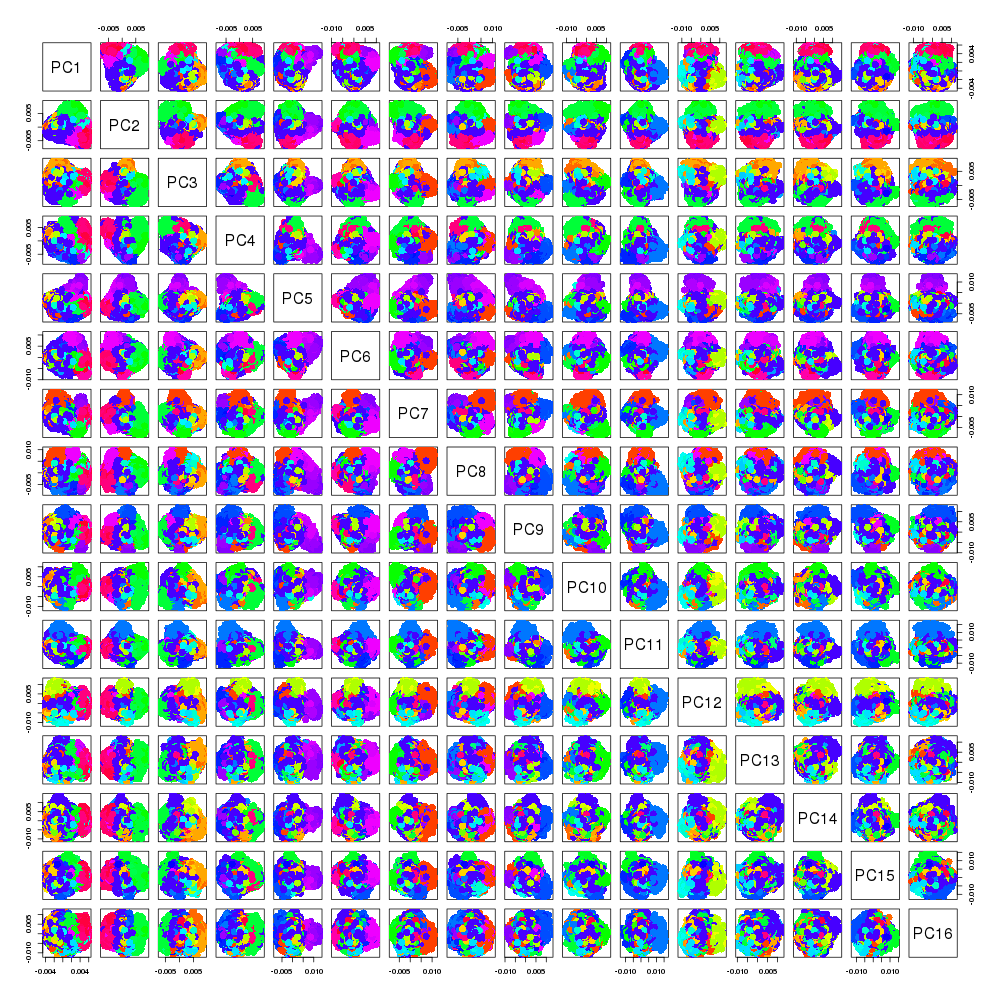

Supplement: Supplementary file 10 — Additional file 10 Pair plots of all the pCA (BrainSpinalCord) implementations. [file 13059_2019_1900_MOESM10_ESM.gz › AdditionalFile10/Sklearn_Incremental.png]

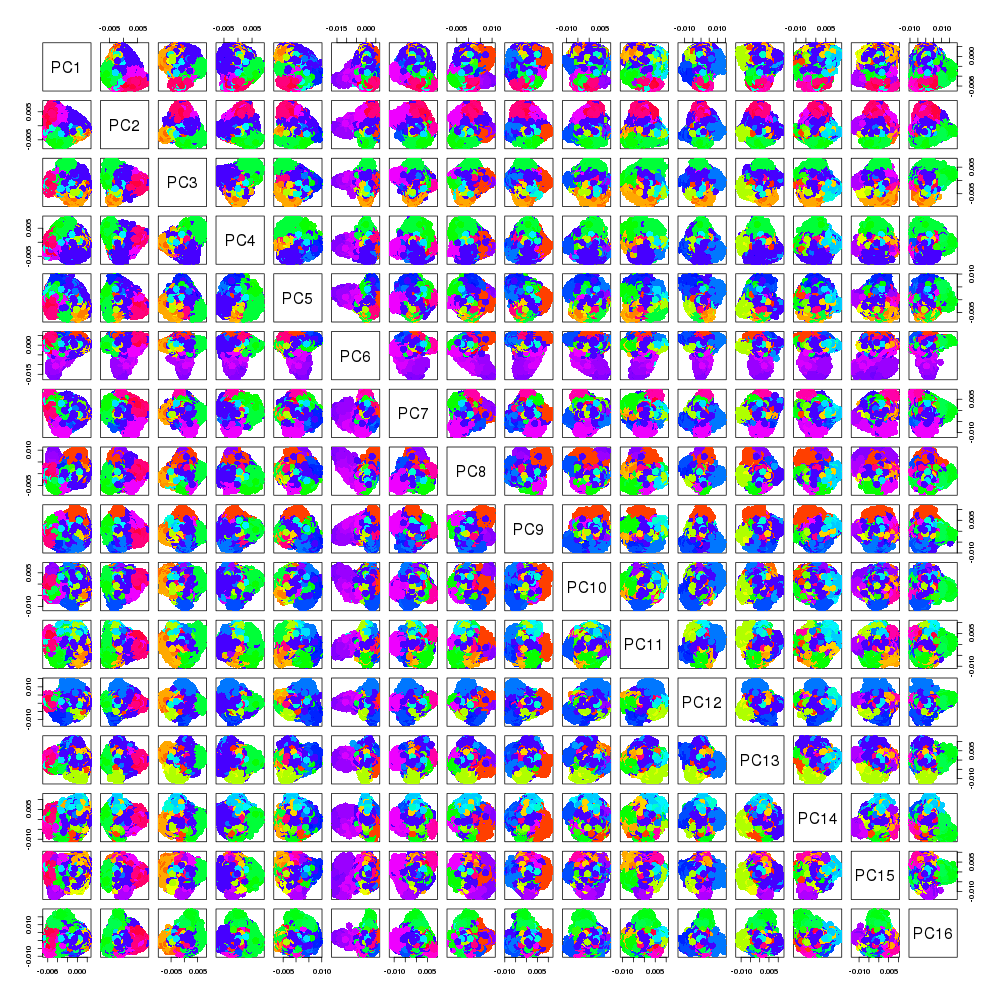

Supplement: Supplementary file 10 — Additional file 10 Pair plots of all the pCA (BrainSpinalCord) implementations. [file 13059_2019_1900_MOESM10_ESM.gz › AdditionalFile10/Halko_3iter.png]

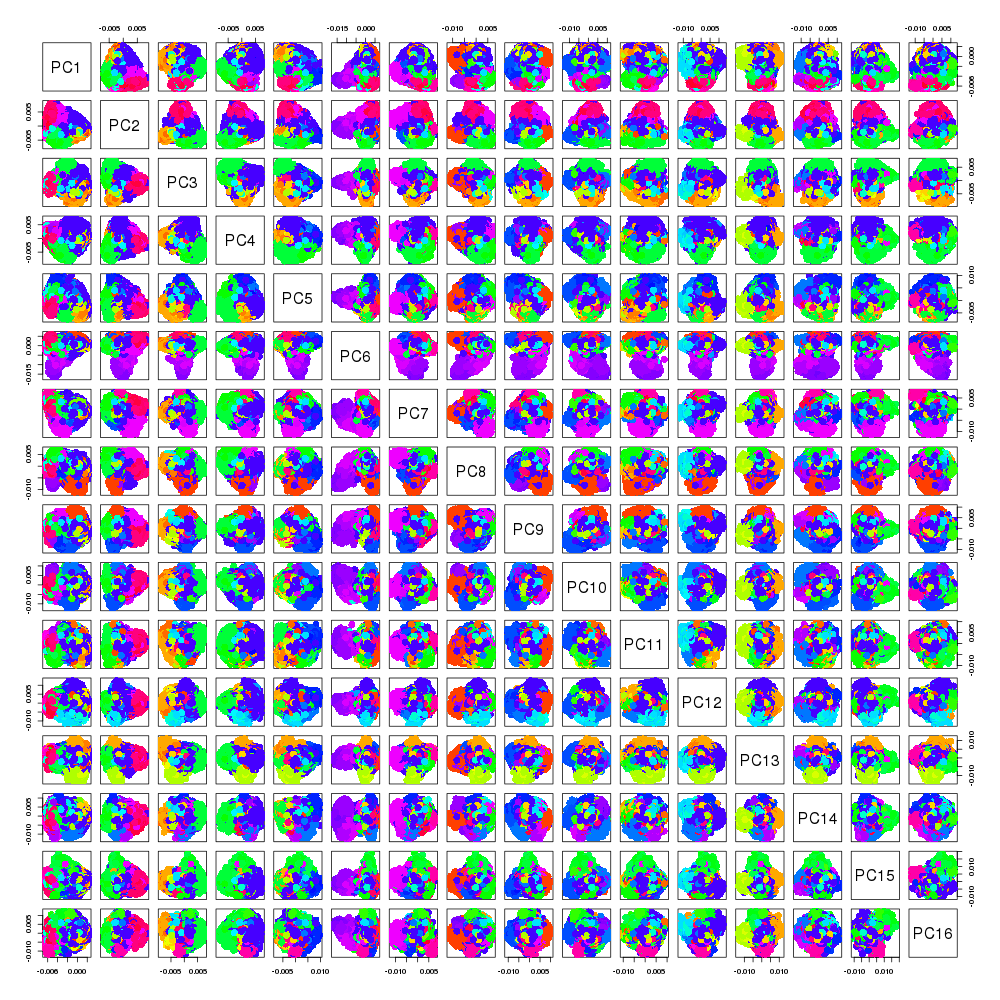

Supplement: Supplementary file 10 — Additional file 10 Pair plots of all the pCA (BrainSpinalCord) implementations. [file 13059_2019_1900_MOESM10_ESM.gz › AdditionalFile10/Halko_1iter.png]

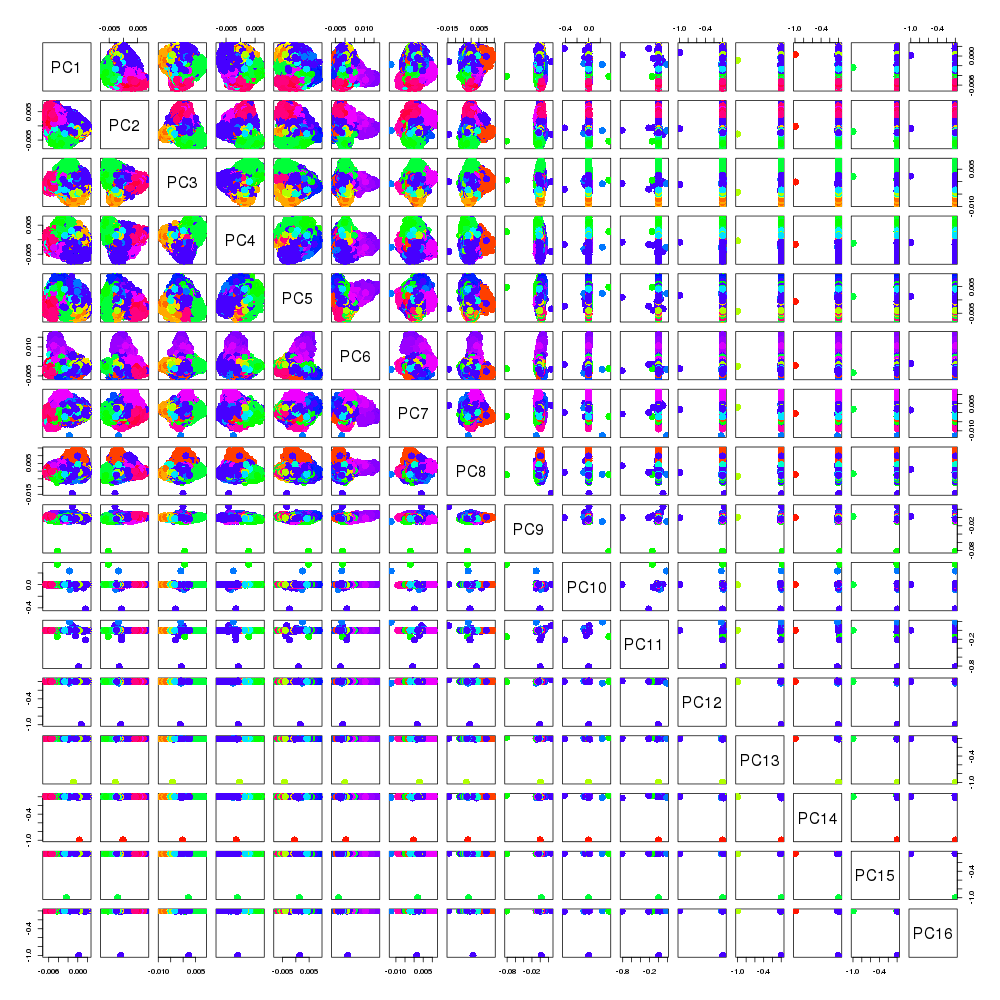

Supplement: Supplementary file 10 — Additional file 10 Pair plots of all the pCA (BrainSpinalCord) implementations. [file 13059_2019_1900_MOESM10_ESM.gz › AdditionalFile10/SGD_step100_epoch10.png]

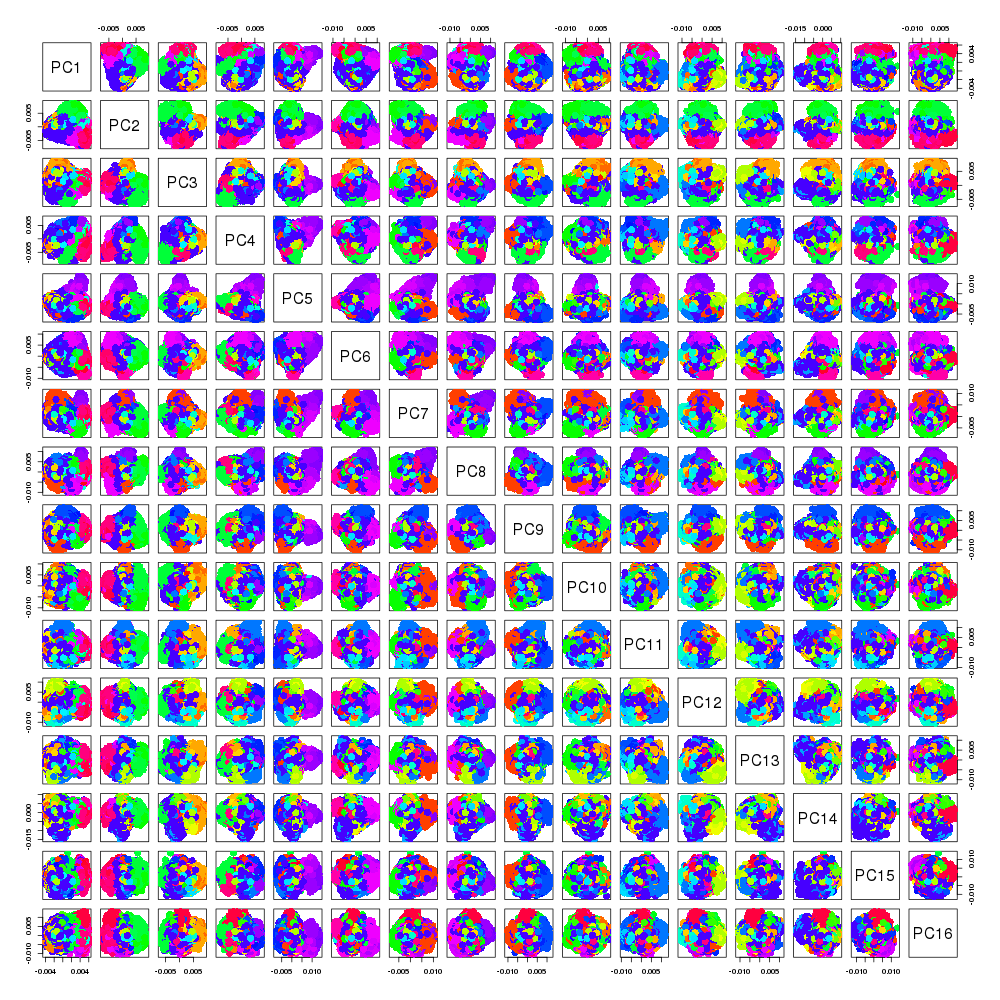

Supplement: Supplementary file 10 — Additional file 10 Pair plots of all the pCA (BrainSpinalCord) implementations. [file 13059_2019_1900_MOESM10_ESM.gz › AdditionalFile10/oocRPCA.png]

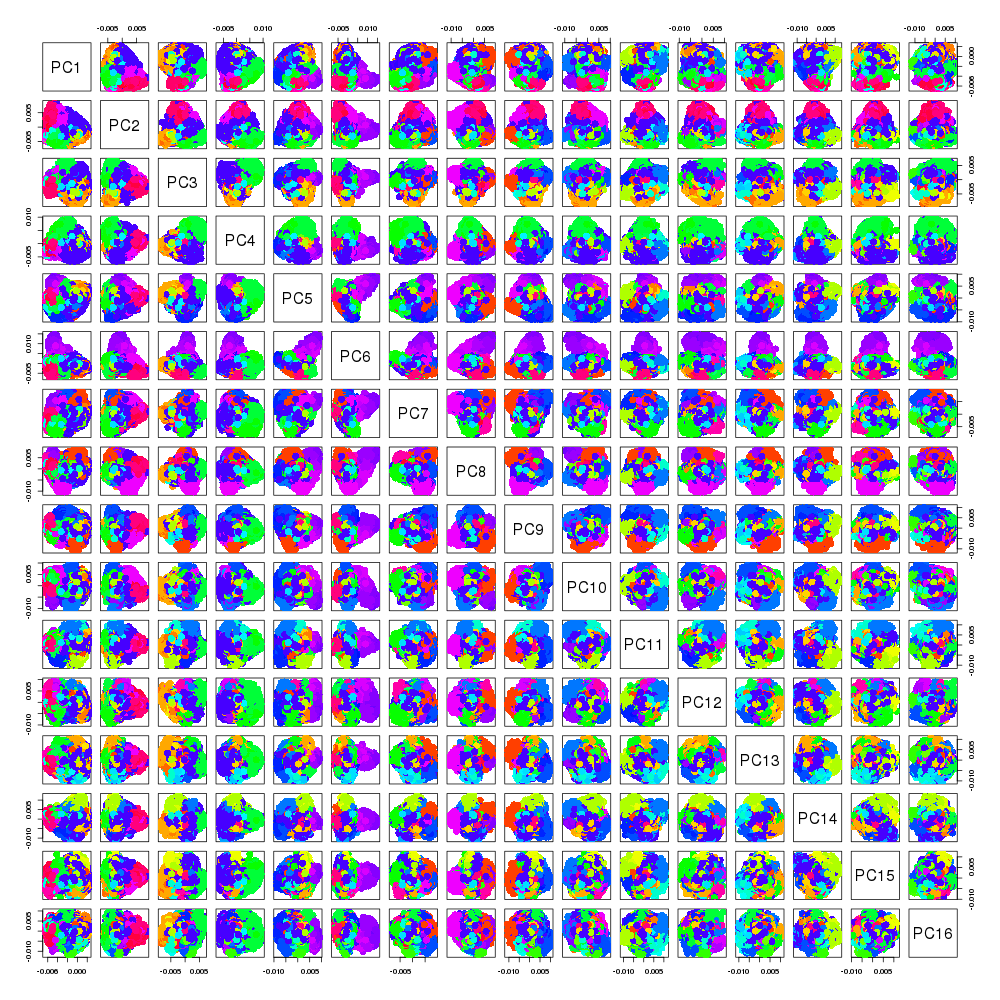

Supplement: Supplementary file 10 — Additional file 10 Pair plots of all the pCA (BrainSpinalCord) implementations. [file 13059_2019_1900_MOESM10_ESM.gz › AdditionalFile10/OrthIter.png]

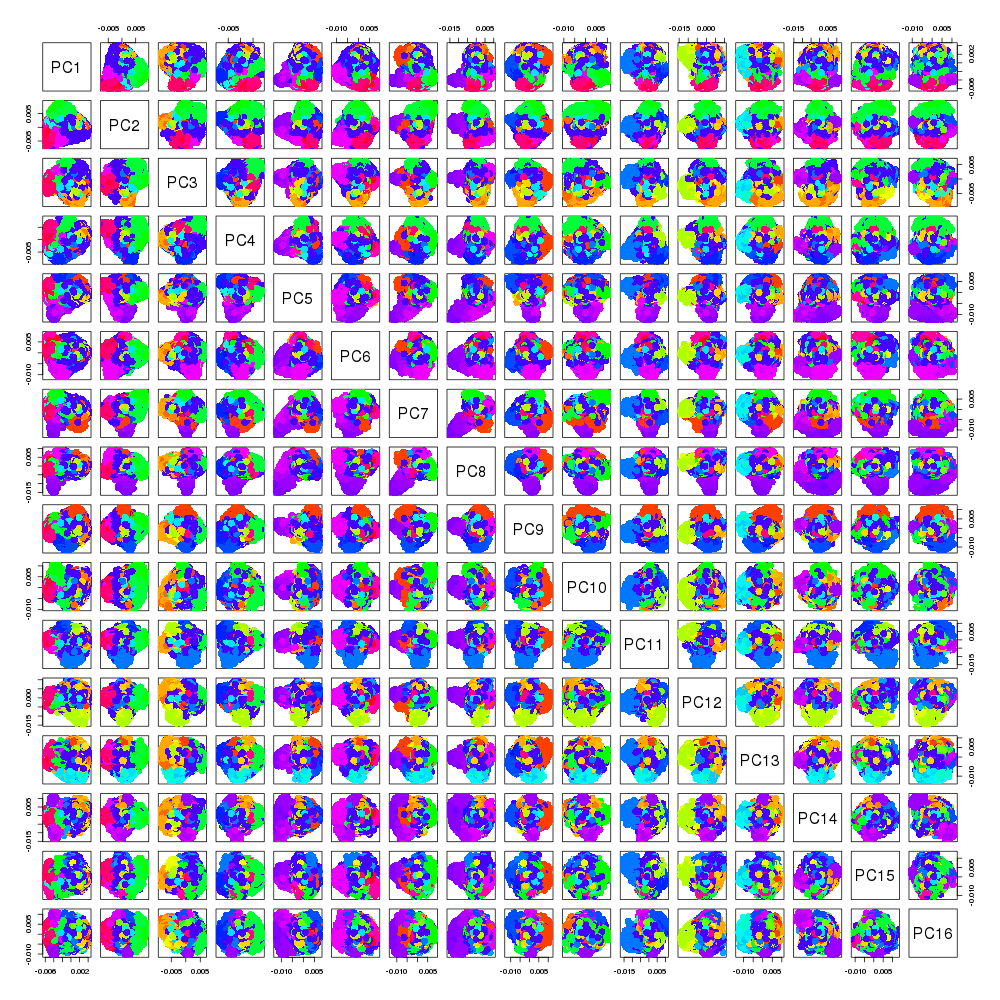

Supplement: Supplementary file 10 — Additional file 10 Pair plots of all the pCA (BrainSpinalCord) implementations. [file 13059_2019_1900_MOESM10_ESM.gz › AdditionalFile10/Downsampling.png]

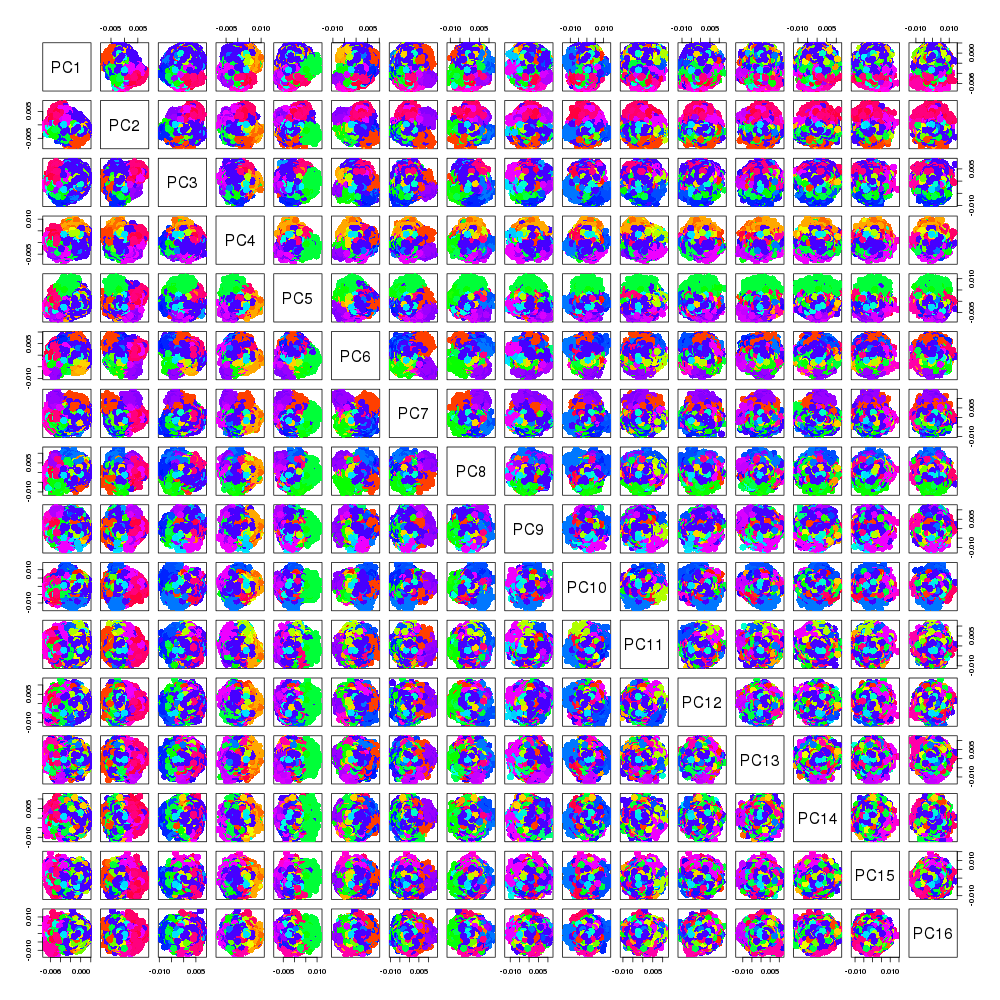

Supplement: Supplementary file 10 — Additional file 10 Pair plots of all the pCA (BrainSpinalCord) implementations. [file 13059_2019_1900_MOESM10_ESM.gz › AdditionalFile10/Halko_0iter.png]

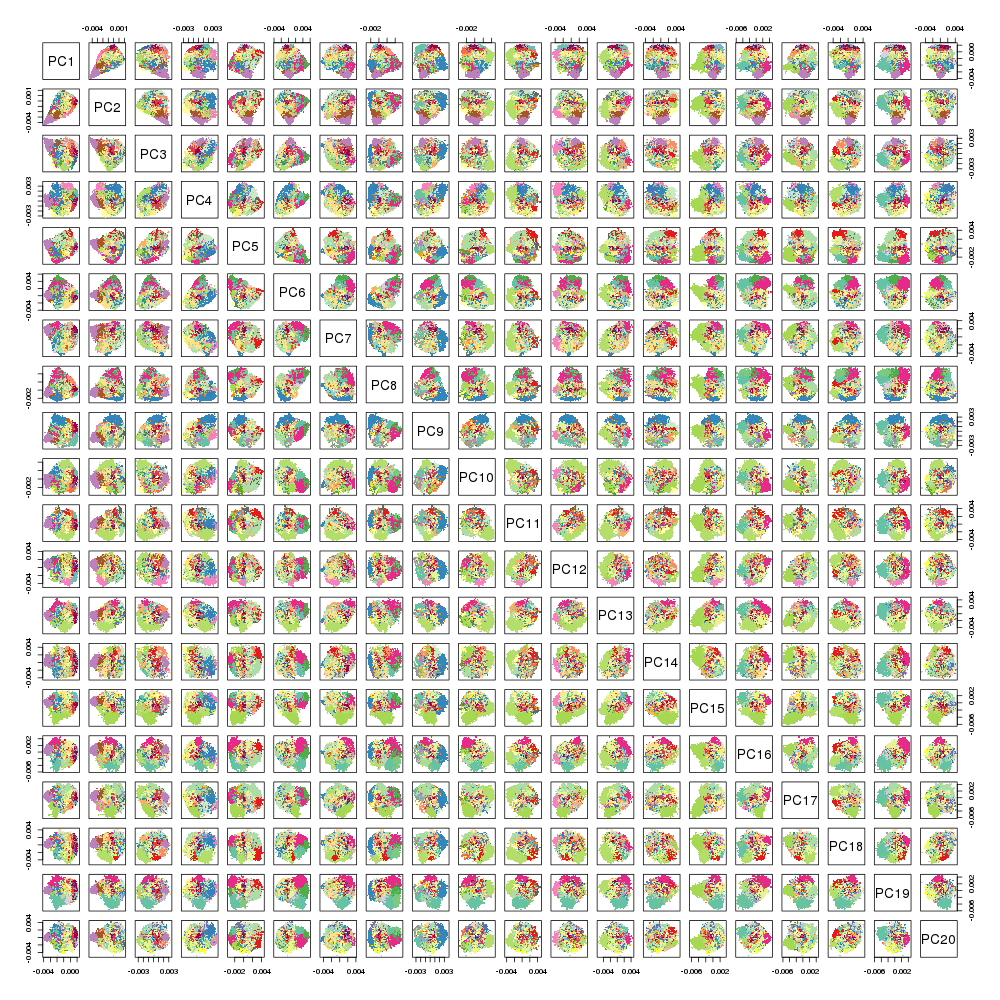

Supplement: Supplementary file 11 — Additional file 11 Pair plots of all the pCA (Brain) implementations. [file 13059_2019_1900_MOESM11_ESM.gz › AdditionalFile11/GD_step1000_epoch10.png]

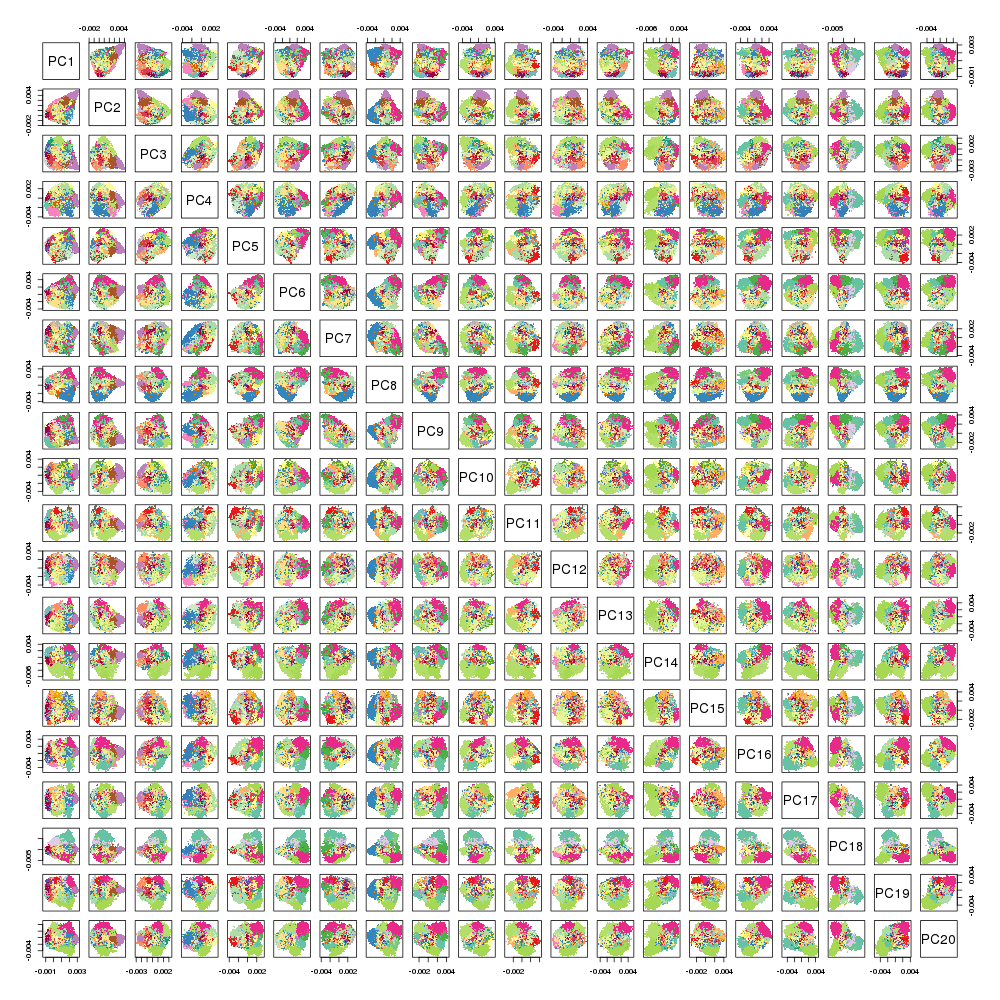

Supplement: Supplementary file 11 — Additional file 11 Pair plots of all the pCA (Brain) implementations. [file 13059_2019_1900_MOESM11_ESM.gz › AdditionalFile11/Algorithm971_3iter.png]

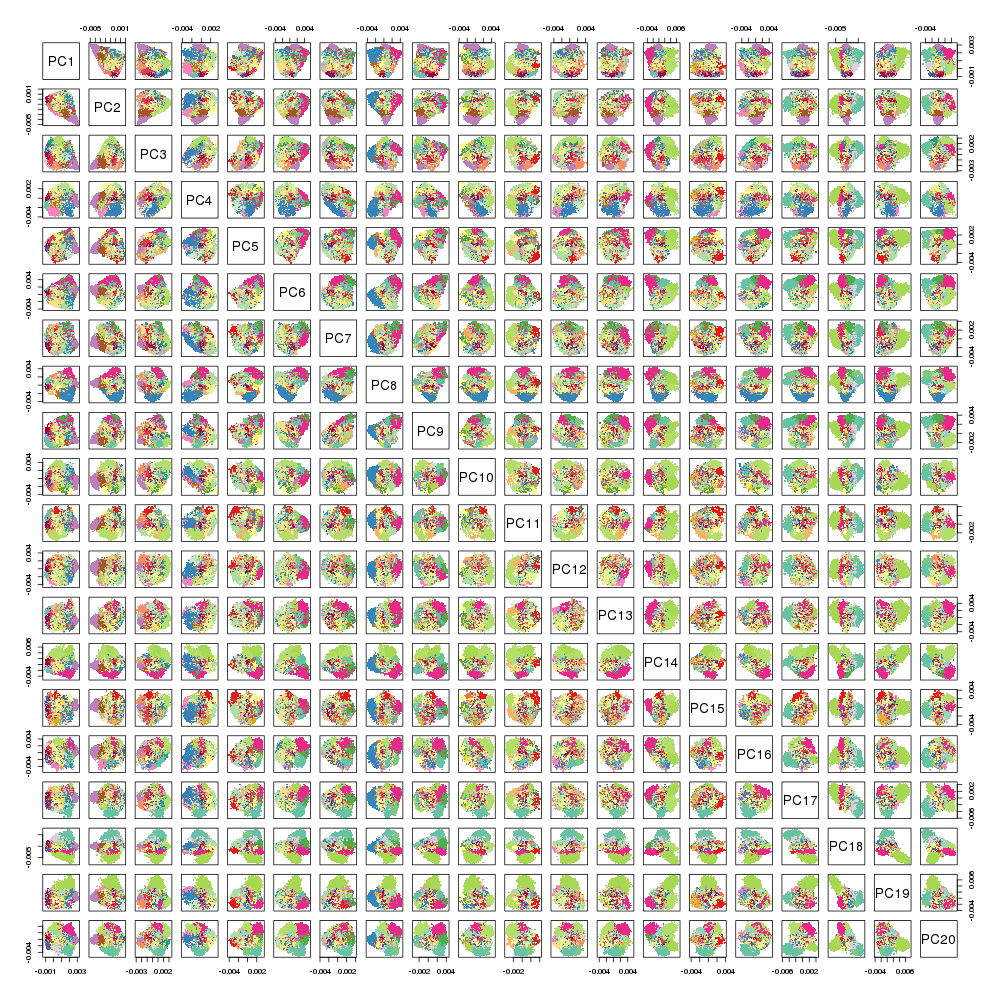

Supplement: Supplementary file 11 — Additional file 11 Pair plots of all the pCA (Brain) implementations. [file 13059_2019_1900_MOESM11_ESM.gz › AdditionalFile11/Halko_2iter.png]

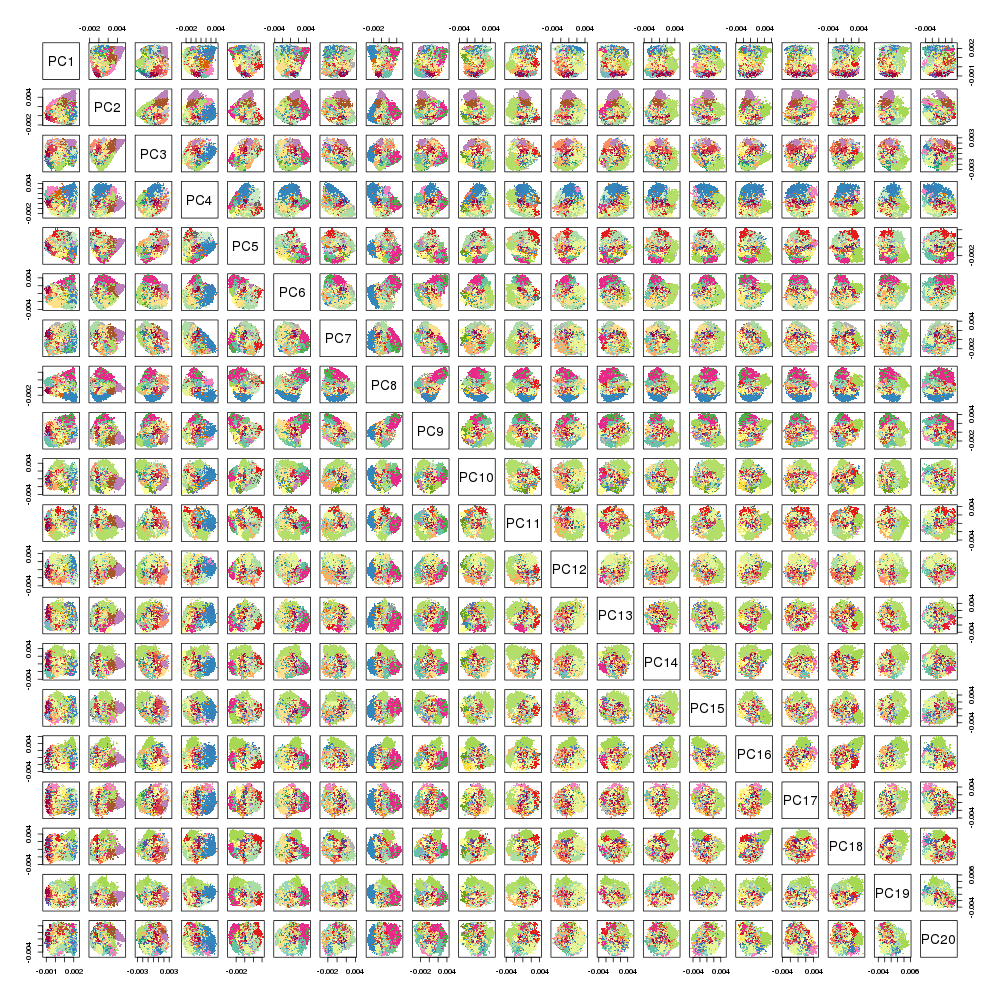

Supplement: Supplementary file 11 — Additional file 11 Pair plots of all the pCA (Brain) implementations. [file 13059_2019_1900_MOESM11_ESM.gz › AdditionalFile11/Sklearn_Incremental.png]

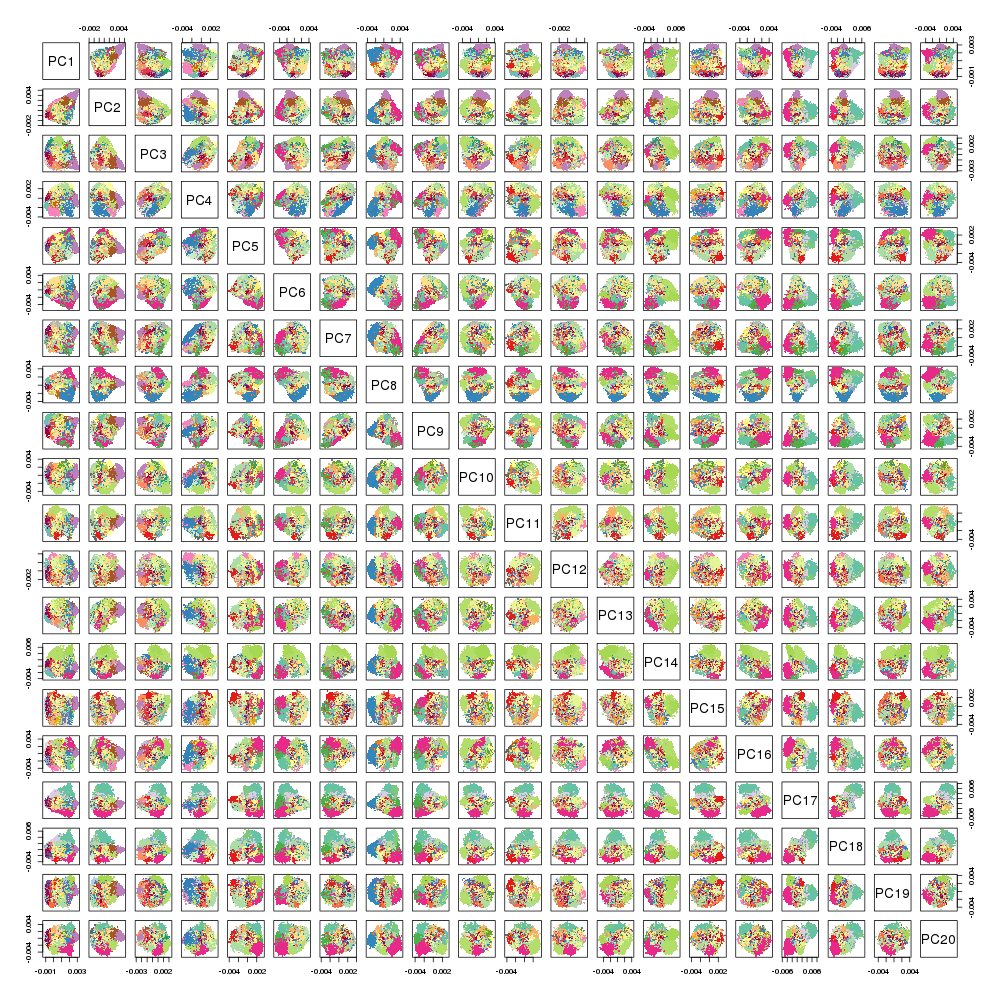

Supplement: Supplementary file 11 — Additional file 11 Pair plots of all the pCA (Brain) implementations. [file 13059_2019_1900_MOESM11_ESM.gz › AdditionalFile11/Halko_3iter.png]

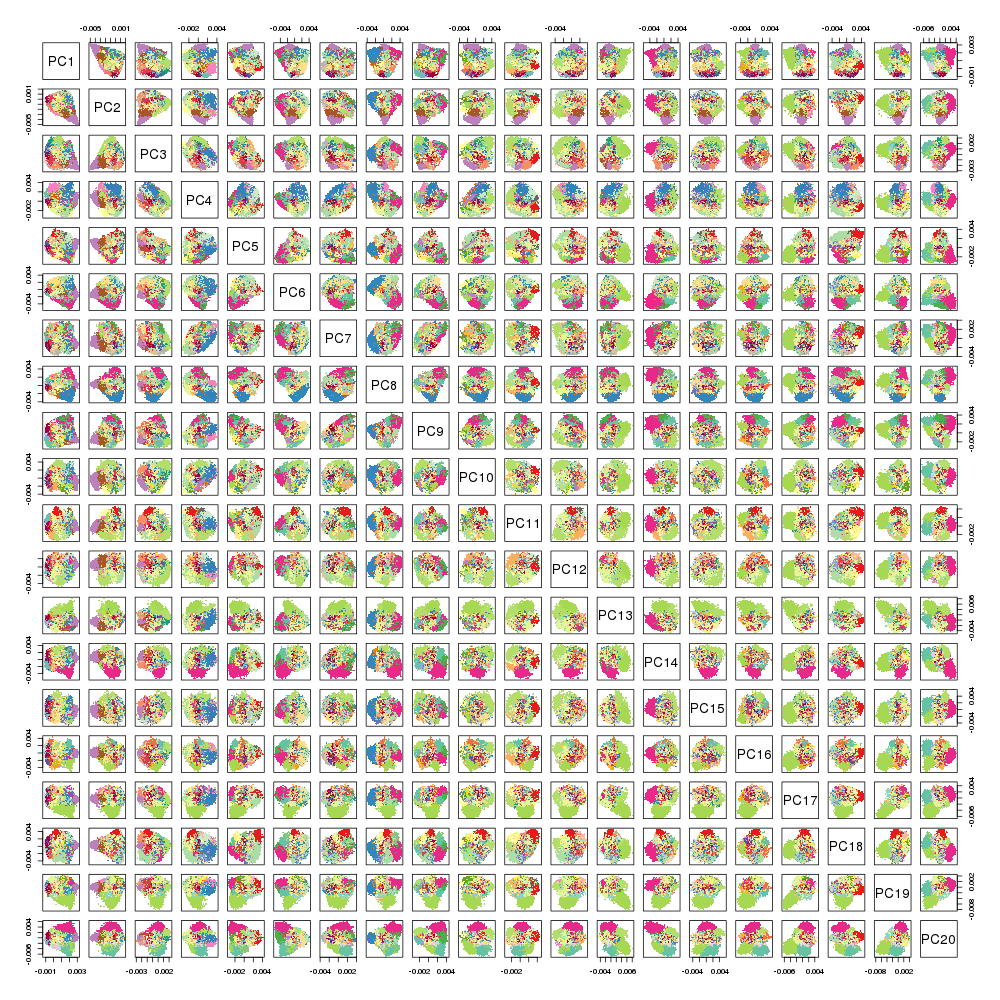

Supplement: Supplementary file 11 — Additional file 11 Pair plots of all the pCA (Brain) implementations. [file 13059_2019_1900_MOESM11_ESM.gz › AdditionalFile11/Halko_1iter.png]

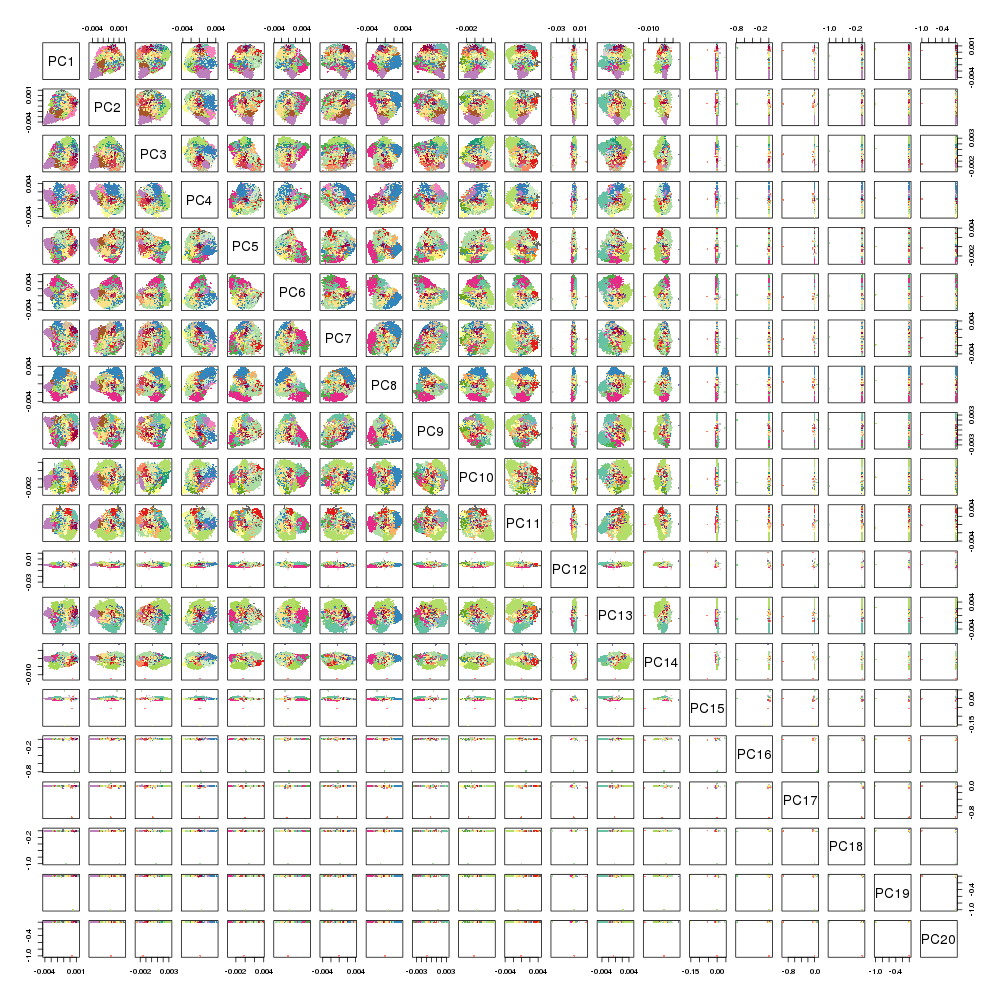

Supplement: Supplementary file 11 — Additional file 11 Pair plots of all the pCA (Brain) implementations. [file 13059_2019_1900_MOESM11_ESM.gz › AdditionalFile11/SGD_step100_epoch10.png]

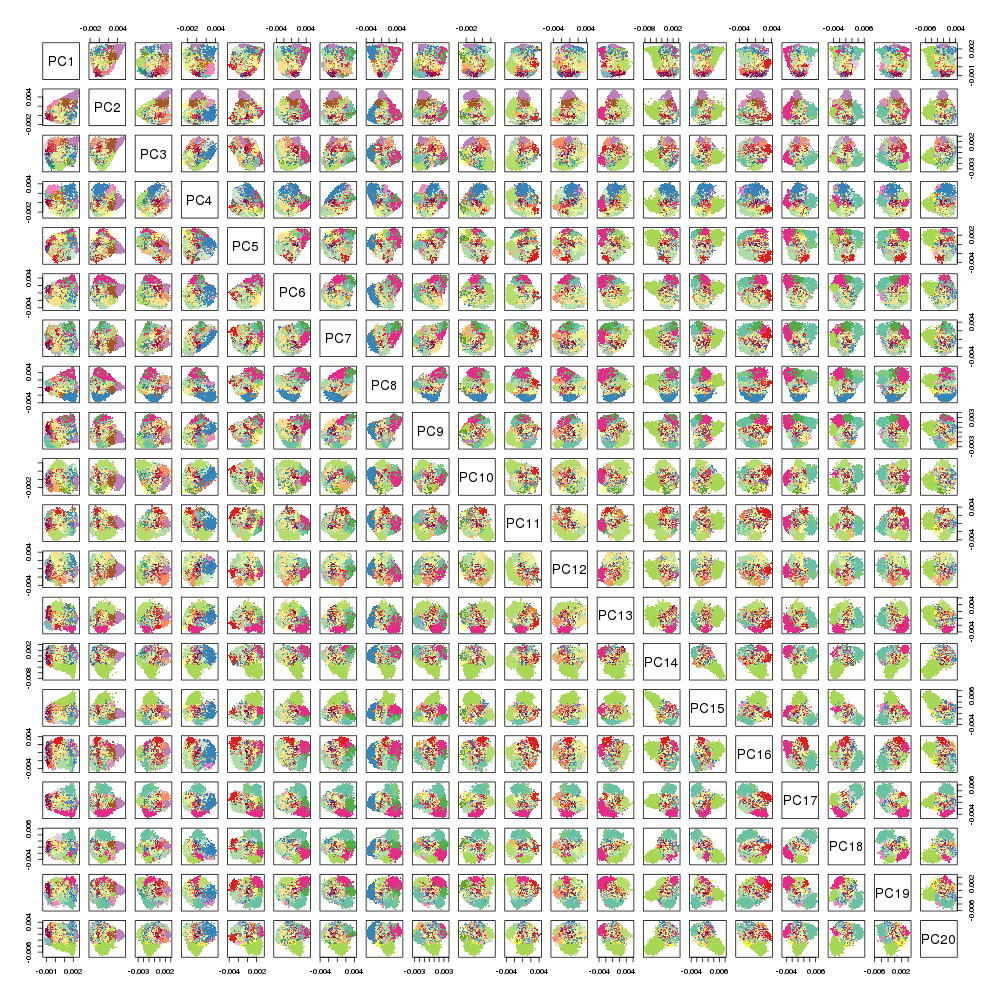

Supplement: Supplementary file 11 — Additional file 11 Pair plots of all the pCA (Brain) implementations. [file 13059_2019_1900_MOESM11_ESM.gz › AdditionalFile11/oocRPCA.png]

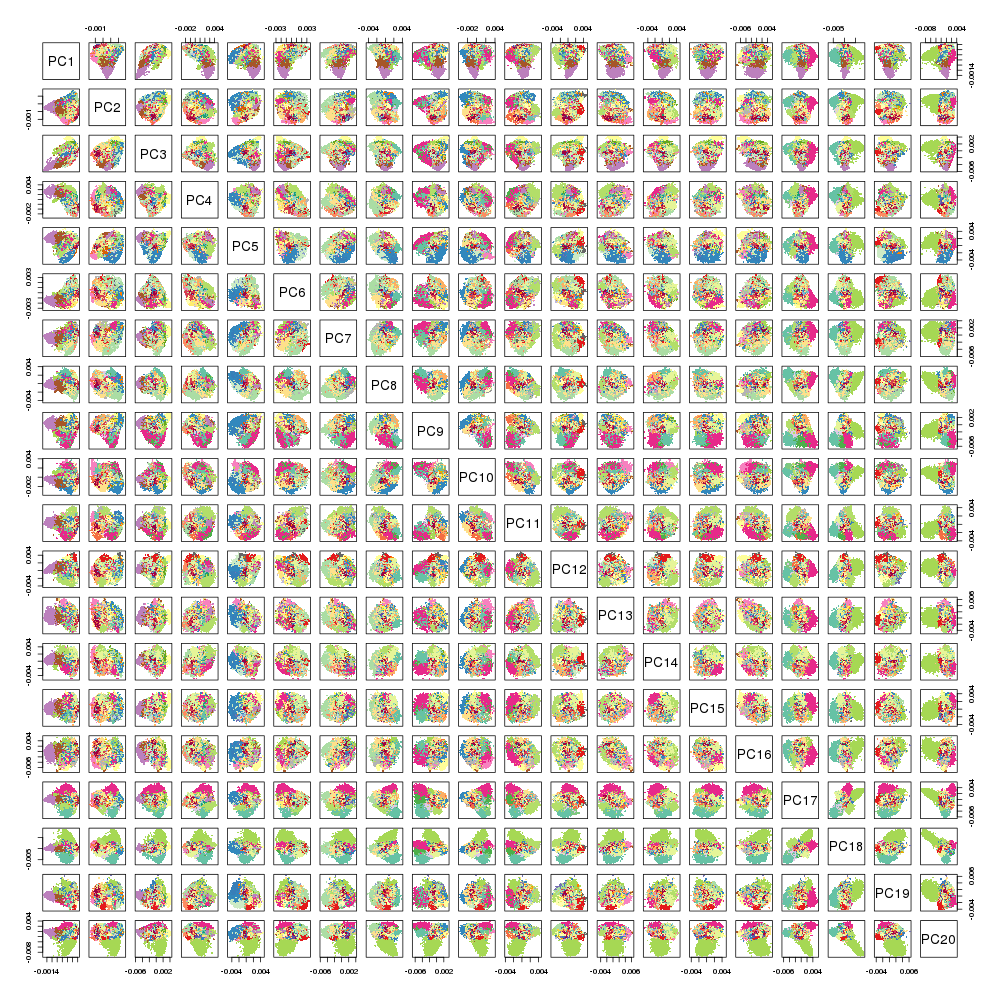

Supplement: Supplementary file 11 — Additional file 11 Pair plots of all the pCA (Brain) implementations. [file 13059_2019_1900_MOESM11_ESM.gz › AdditionalFile11/OrthIter.png]

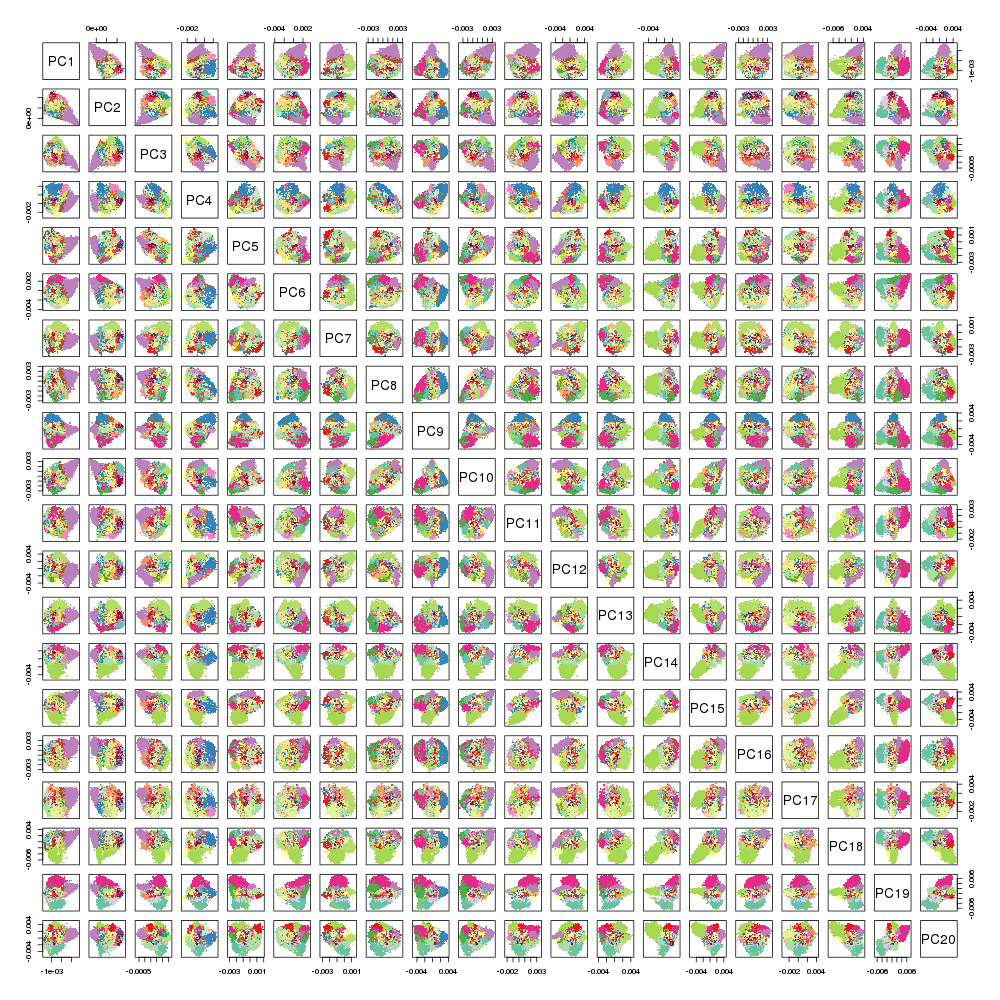

Supplement: Supplementary file 11 — Additional file 11 Pair plots of all the pCA (Brain) implementations. [file 13059_2019_1900_MOESM11_ESM.gz › AdditionalFile11/Downsampling.png]

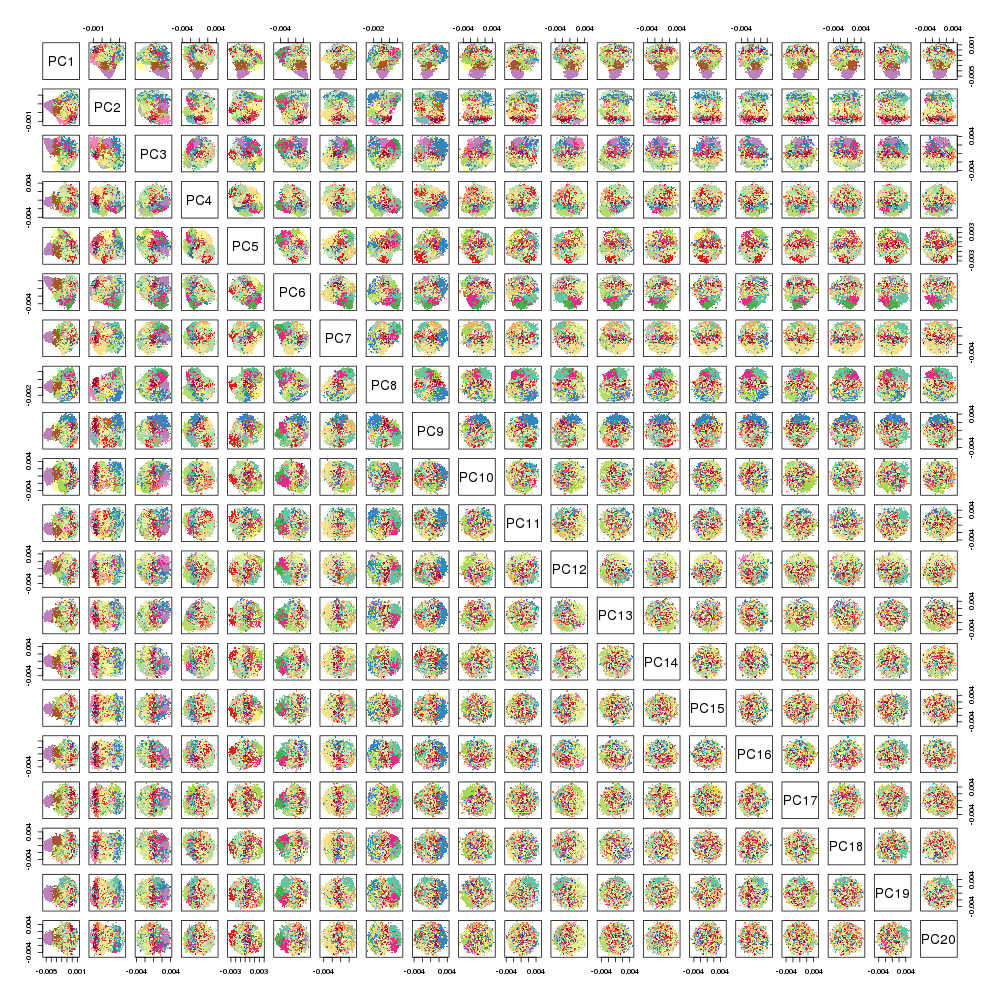

Supplement: Supplementary file 11 — Additional file 11 Pair plots of all the pCA (Brain) implementations. [file 13059_2019_1900_MOESM11_ESM.gz › AdditionalFile11/Halko_0iter.png]

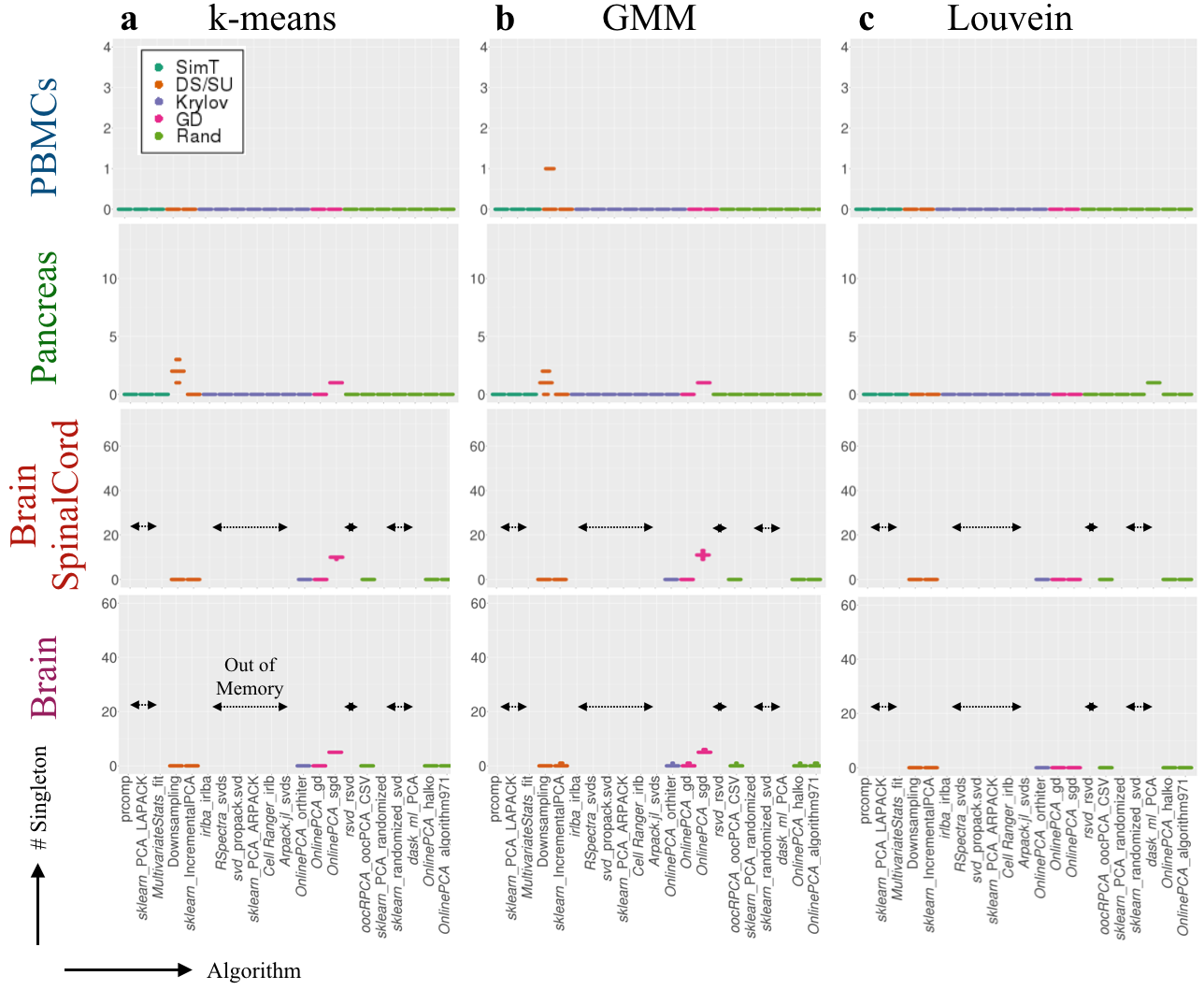

Supplement: Supplementary file 12 — Additional file 12 Number of singleton clusters. [file 13059_2019_1900_MOESM12_ESM.png]

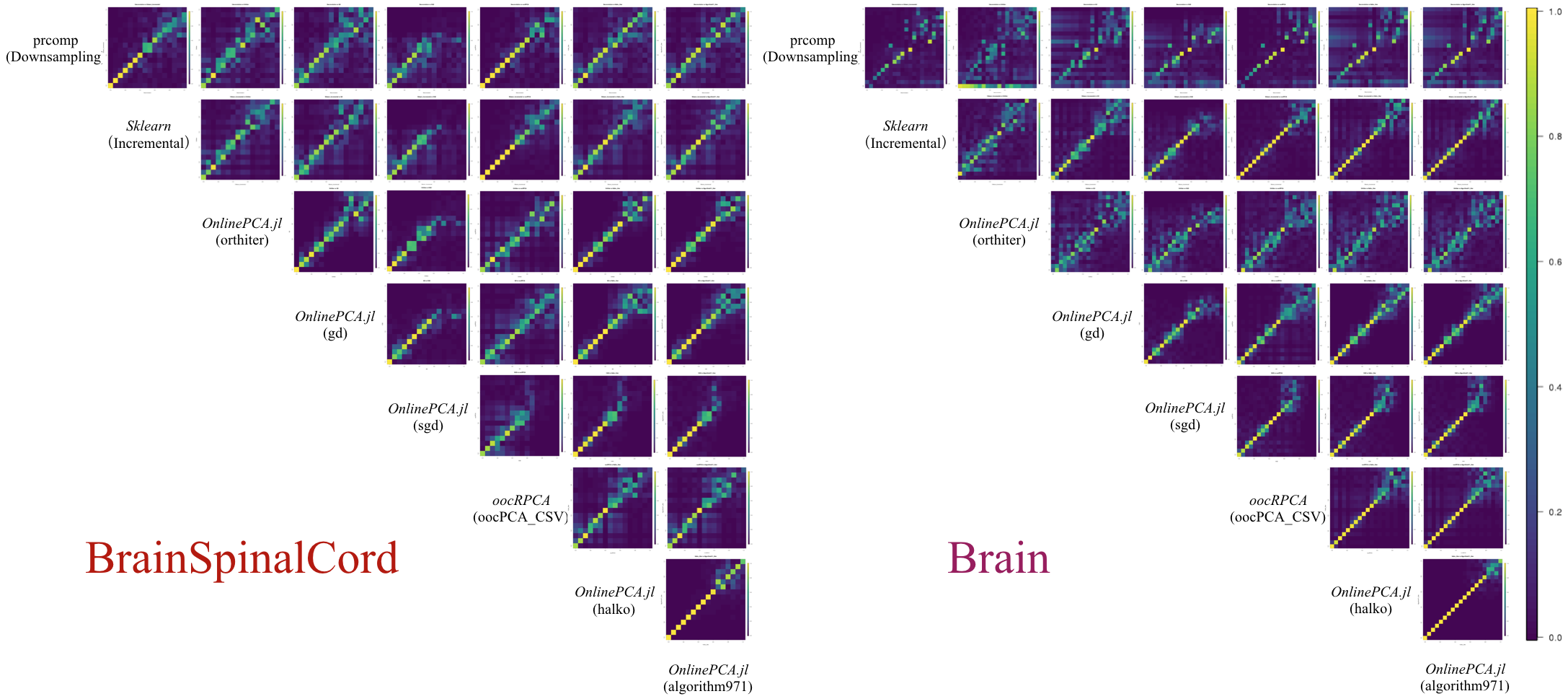

Supplement: Supplementary file 13 — Additional file 13 Eigenvectors of all the pCA implementations (BrainSpinalCord and brain). [file 13059_2019_1900_MOESM13_ESM.png]

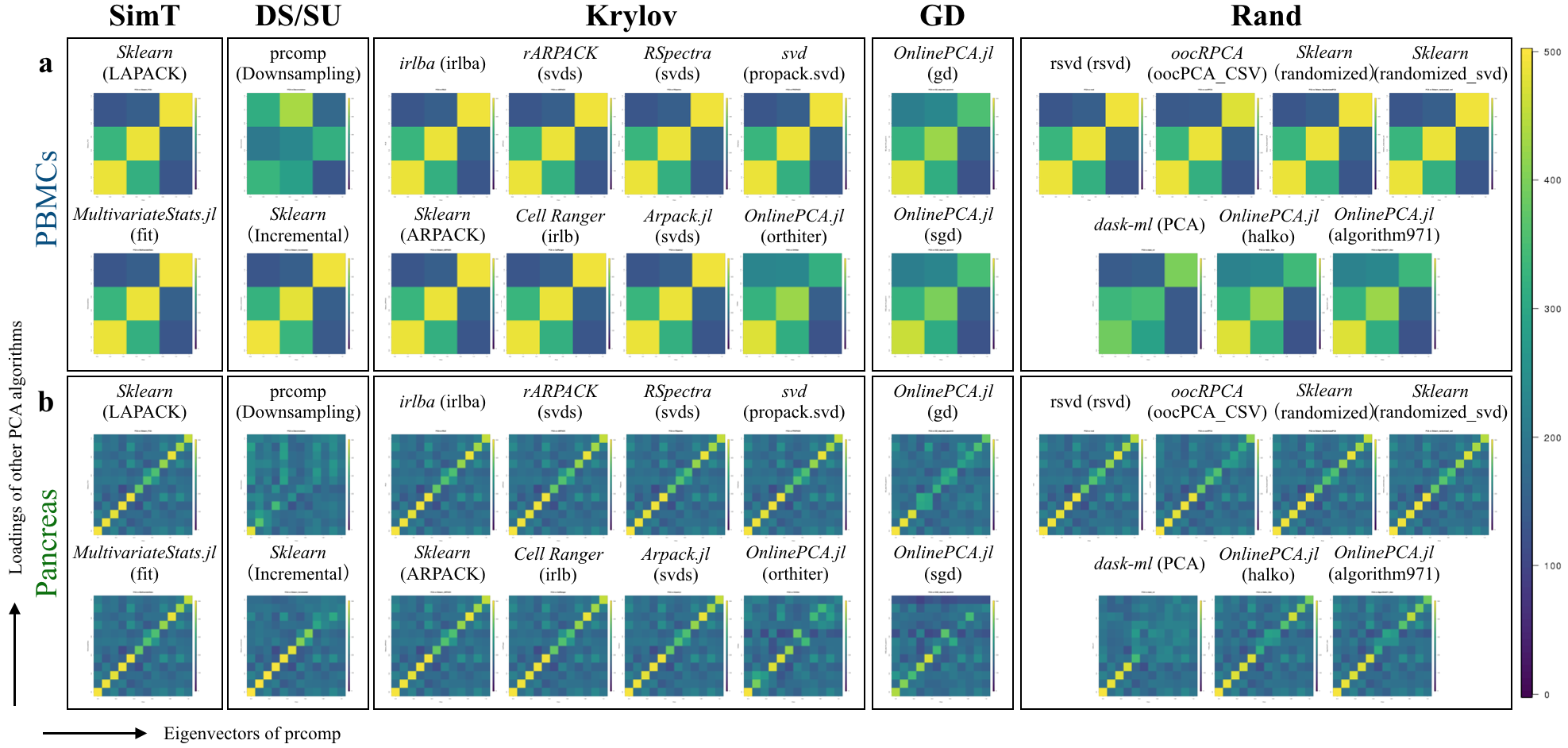

Supplement: Supplementary file 14 — Additional file 14 Loading vectors of all the pCA implementations (PBMCs and pancreas). [file 13059_2019_1900_MOESM14_ESM.png]

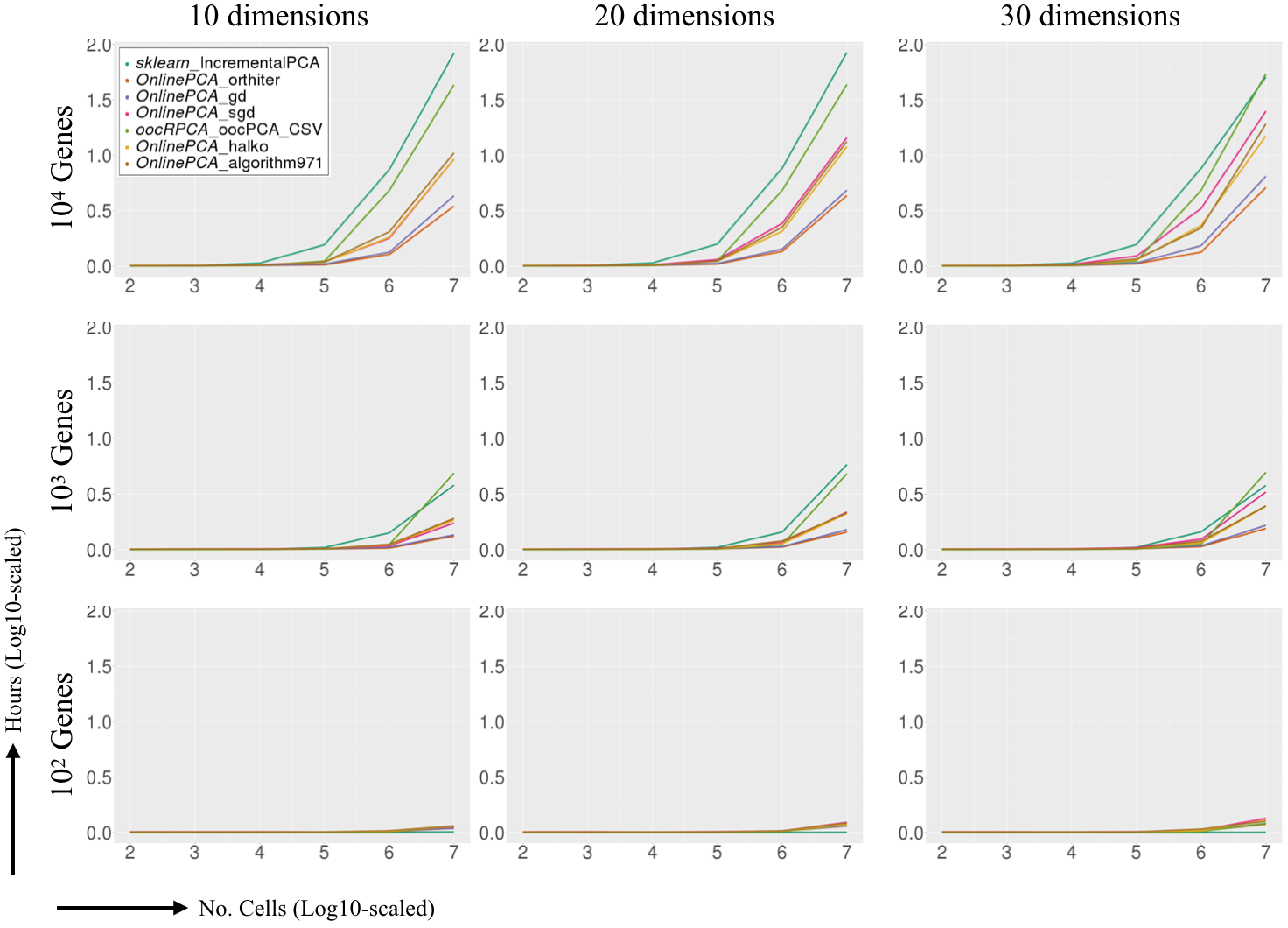

Supplement: Supplementary file 16 — Additional file 16 Comparison of the elapsed time for simulated datasets. [file 13059_2019_1900_MOESM16_ESM.png]

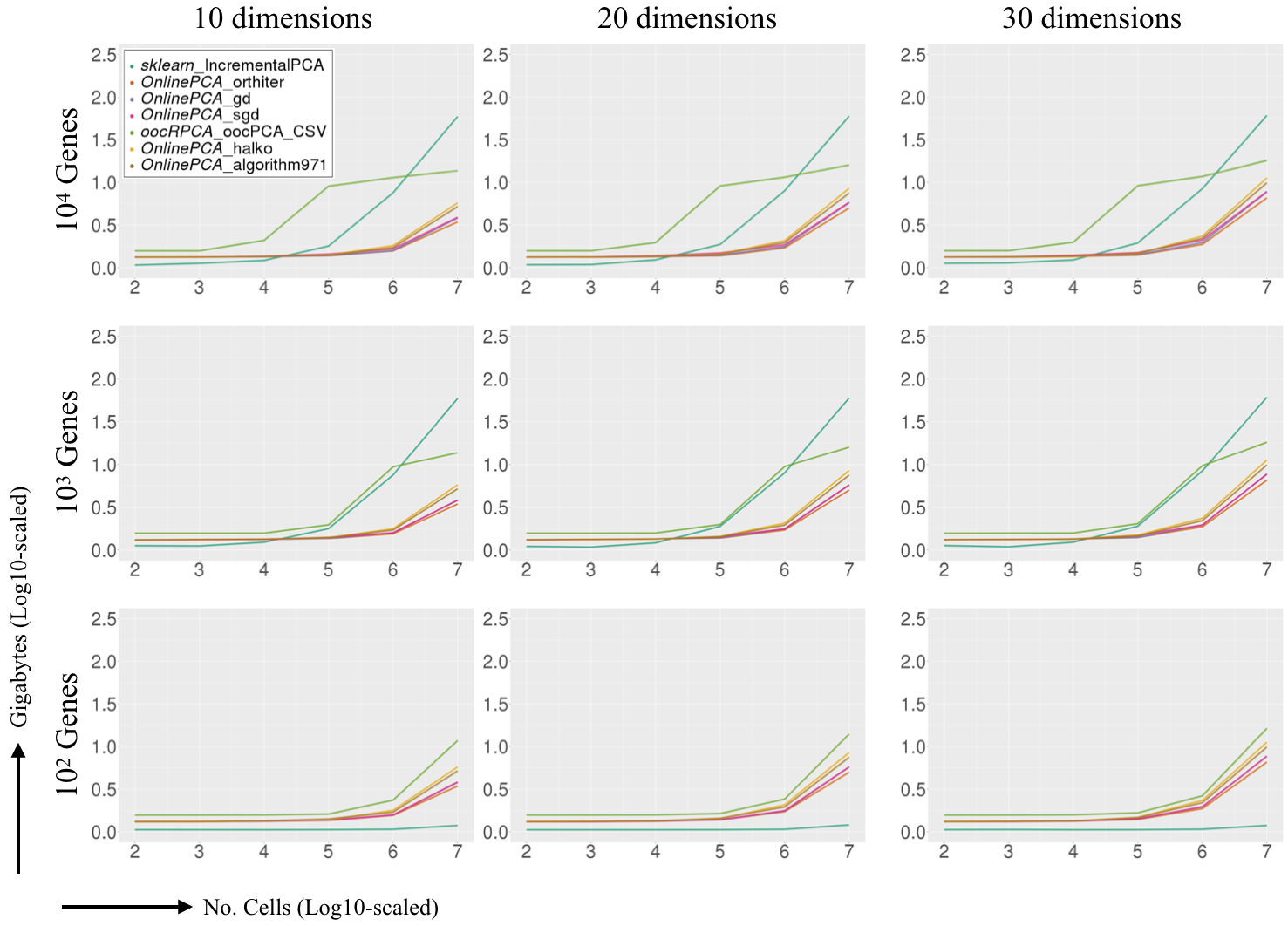

Supplement: Supplementary file 17 — Additional file 17 Comparison of the maximum memory usage for simulated datasets. [file 13059_2019_1900_MOESM17_ESM.png]

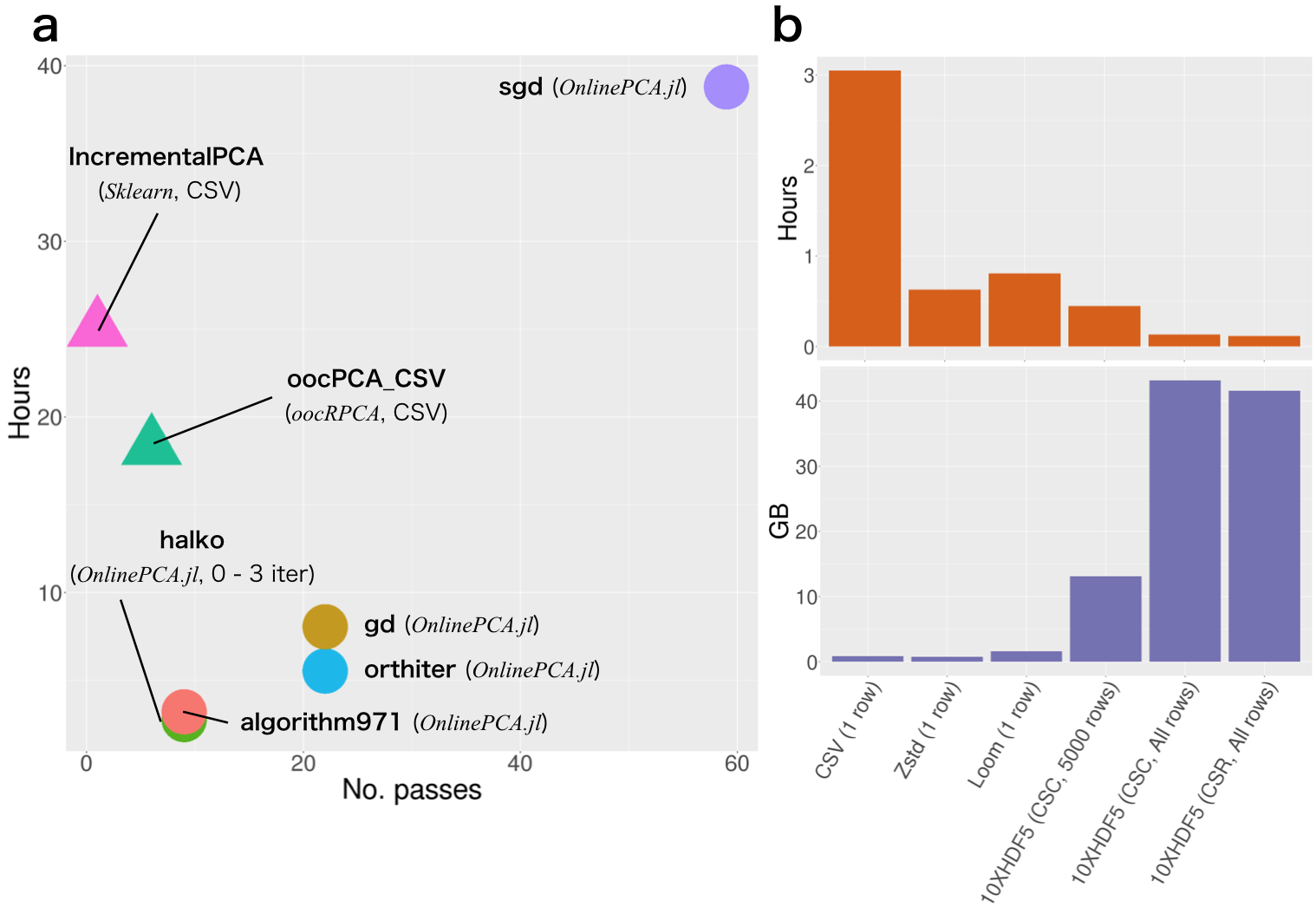

Supplement: Supplementary file 18 — Additional file 18 Relationships of the algorithms/implementations, the number of passes, and the file format with the elapsed time for performing principal component analysis (PCA) with the brain dataset. [file 13059_2019_1900_MOESM18_ESM.png]

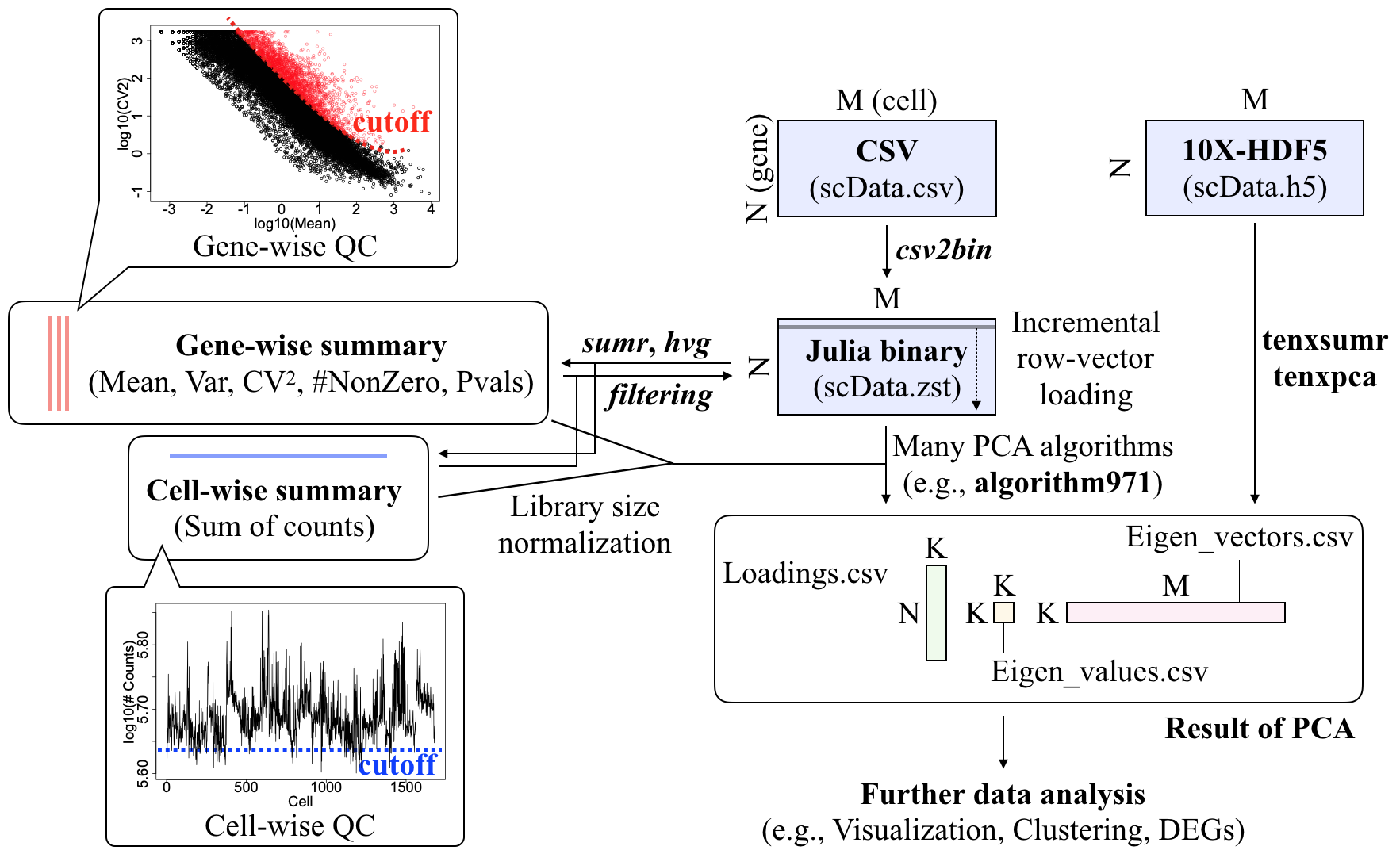

Supplement: Supplementary file 19 — Additional file 19 OnlinePCA.jl schematic. [file 13059_2019_1900_MOESM19_ESM.png]

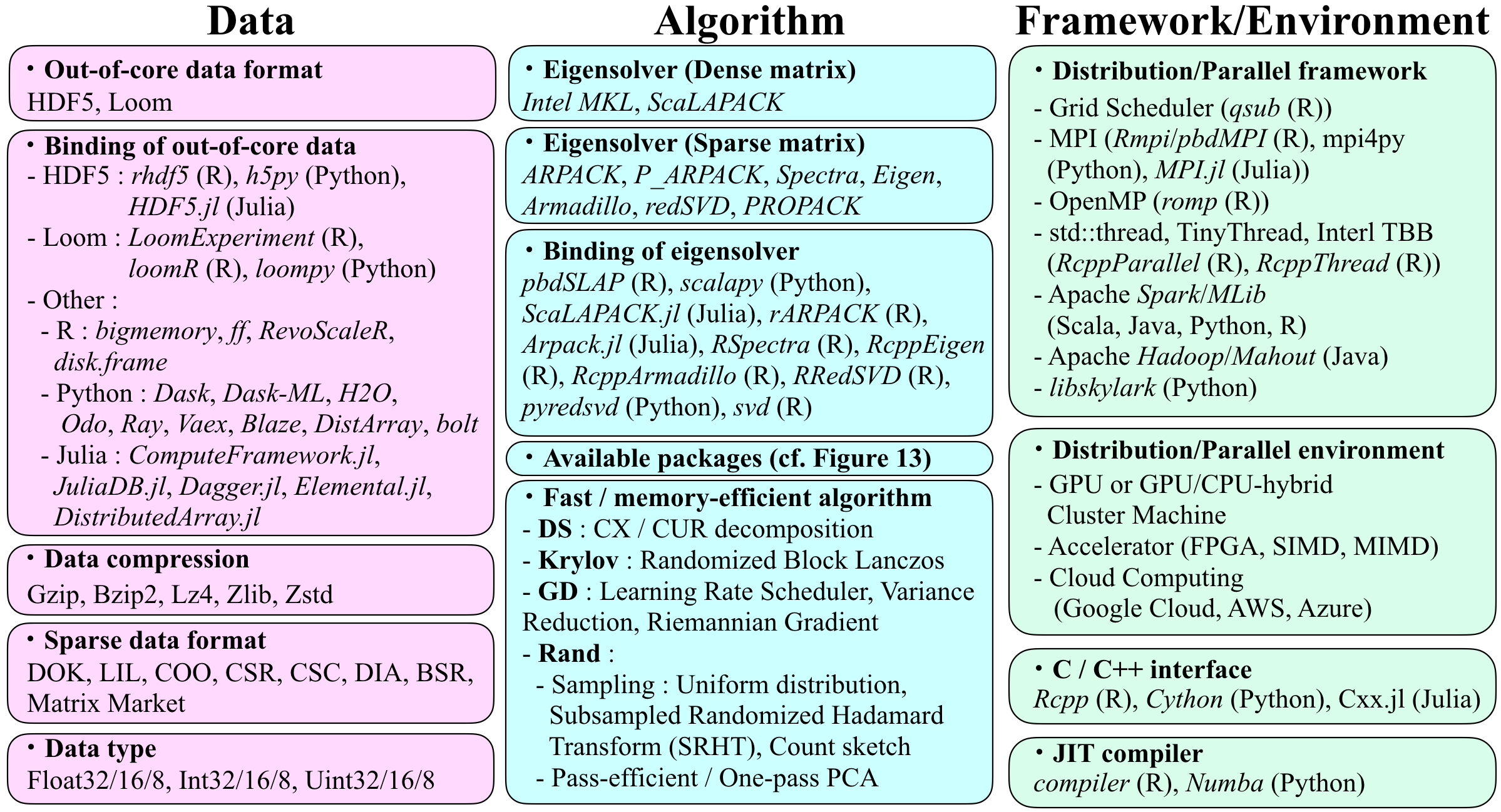

Supplement: Supplementary file 23 — Additional file 23 Developer guidelines. [file 13059_2019_1900_MOESM23_ESM.png]
