# Supplementary material for: Benchmarking principal component analysis for large-scale single-cell RNA-sequencing
Source: Genome Biol. 2020 Jan 20;21:9. doi: 10.1186/s13059-019-1900-3 (PMC6970290; doi:10.1186/s13059-019-1900-3)
Supplement: Supplementary file 6 — Additional file 6 Results of clustering methods of all the pCA implementations. [file 13059_2019_1900_MOESM6_ESM.pdf]

Results of clustering methods for all PCA implementations

Here, we present the results of clustering performed for all the PCA implementations mentioned in the manuscript: *k*-means, Gaussian mixture model (GMM), HDBScan, and Louvain clustering. When performing HDBScan, principal components (PCs) were first converted to five dimensional UMAP coordinates, and then HDBScan was performed (**Figure S6-1** to **7**). When performing Louvain clustering, the Jaccard index was used as the similarity measure with the *k* nearest neighborhood cells. For the PBMCs, Pancreas, BrainSpinalCord, and Brain datasets, *k* values of 100, 75, 10, and 5 were used, respectively.

The cluster assignment shown in **Figure S6-1** to **7** and the corresponding adjusted Rand index (ARI) values (**Figure S6-8**) show that the Louvain clustering results were stable across all datasets. Therefore, we finally selected Louvain clustering as the measure for benchmarking.

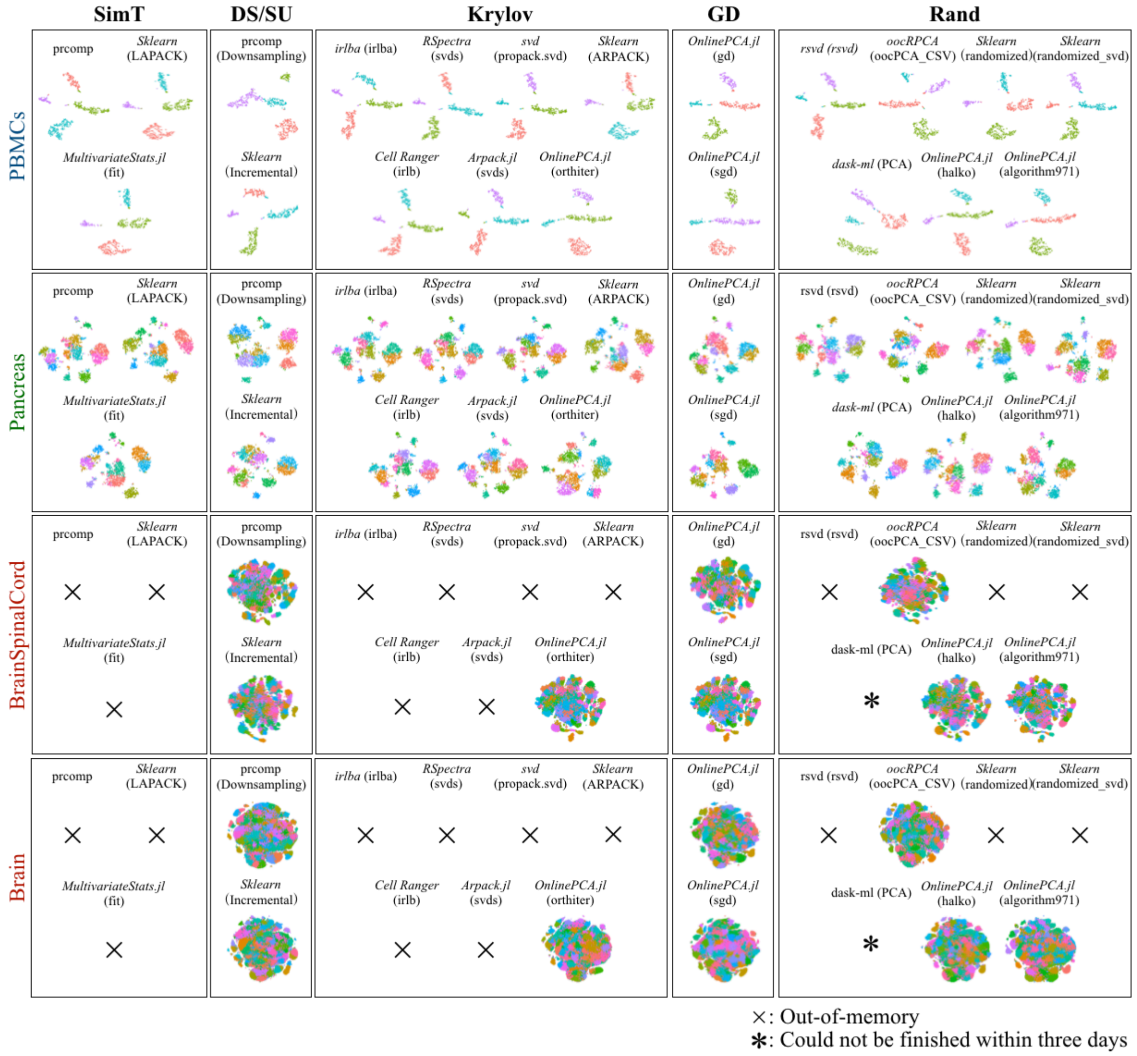

Figure S6-1 | Results of *k*-means clustering The cluster label is visualized with two-dimensional t-SNE coordinates.

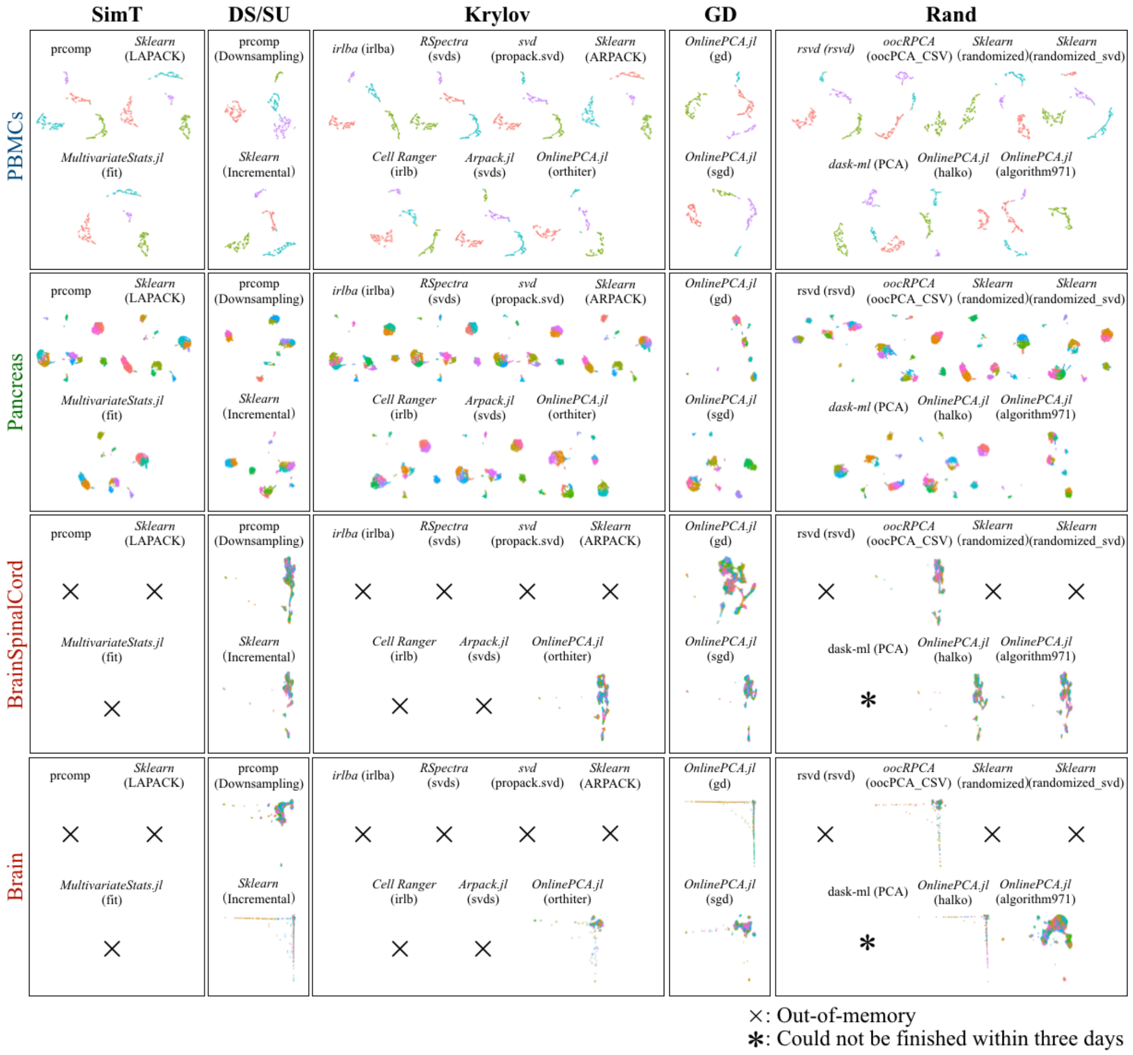

Figure S6-2 | Results of *k*-means clustering The cluster label is visualized with two-dimensional UMAP coordinates.

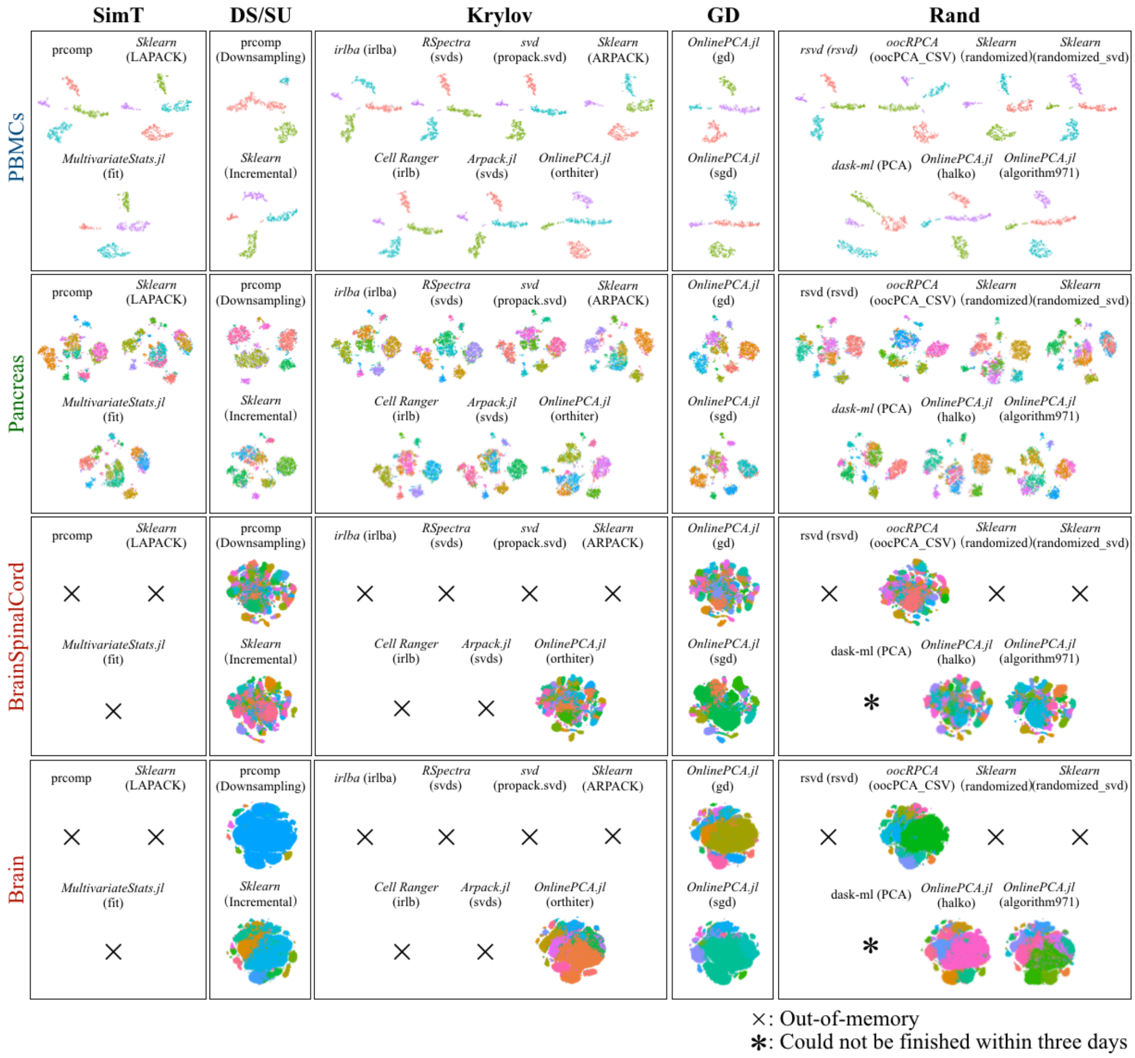

Figure S6-3 | Results of GMM clustering The cluster label is visualized with two-dimensional t-SNE coordinates.

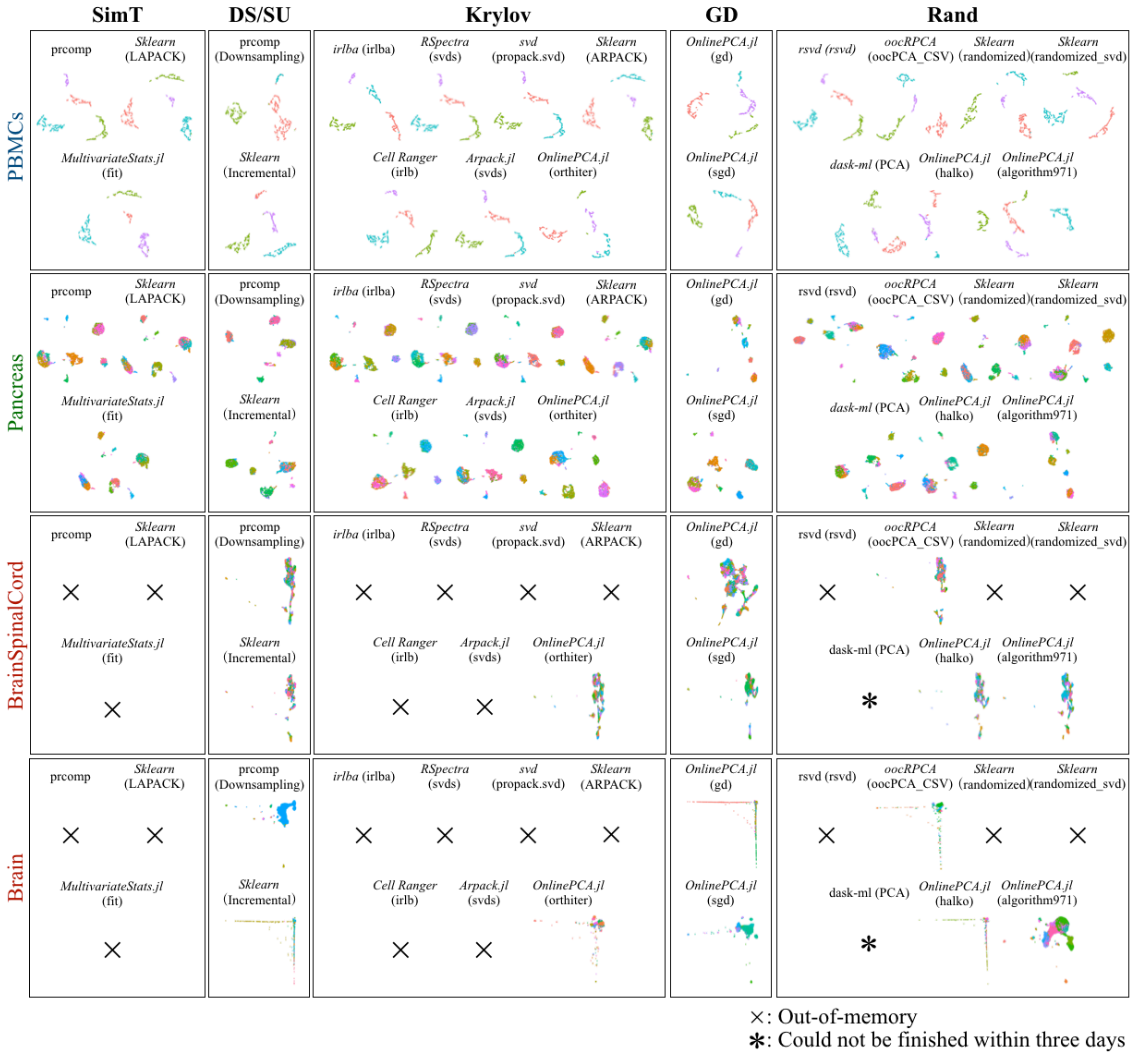

Figure S6-4 | Results of GMM clustering The cluster label is visualized with two-dimensional UMAP coordinates.

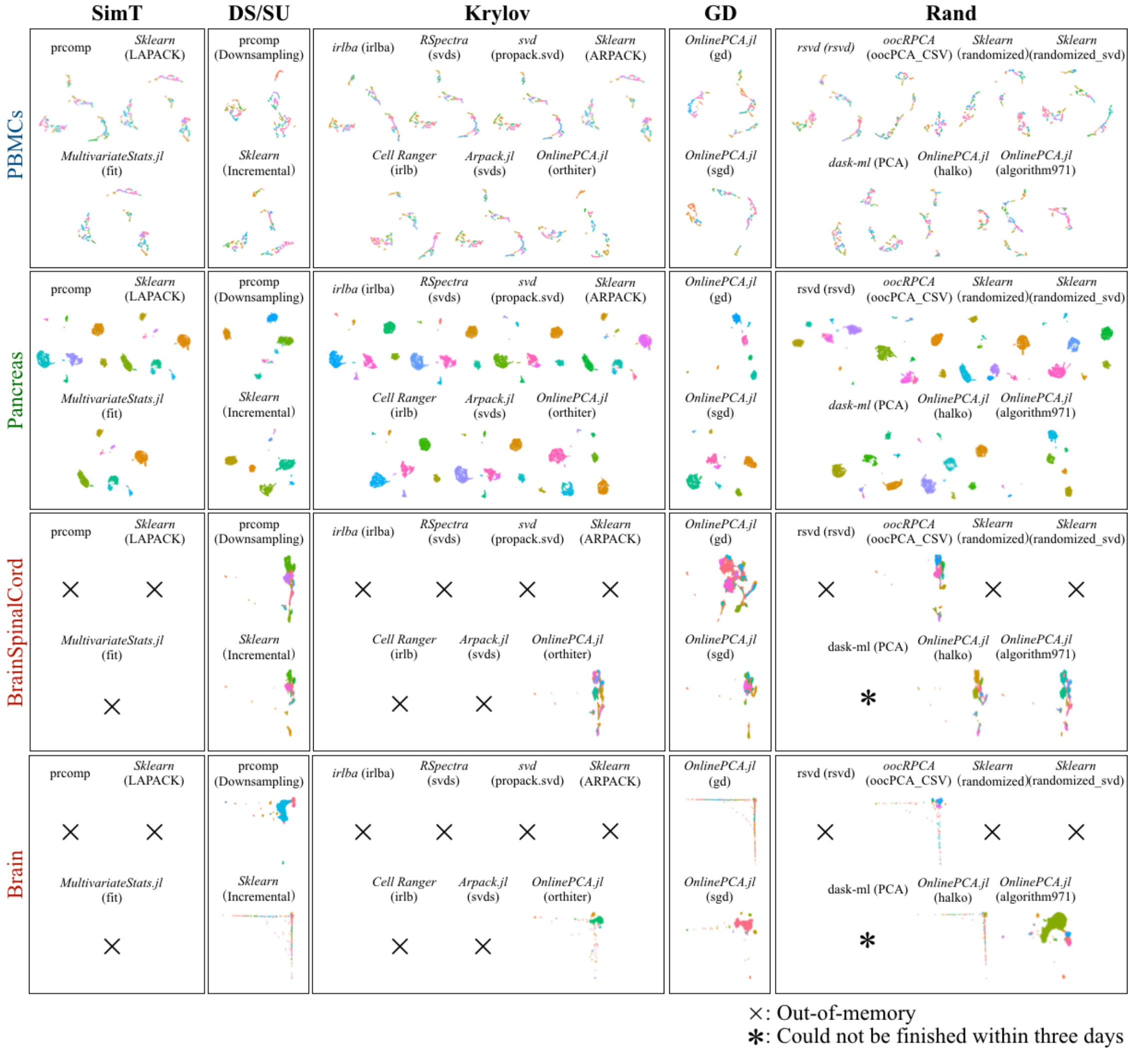

Figure S6-5 | Results of HDBScan clustering The cluster label is visualized with two-dimensional UMAP coordinates.

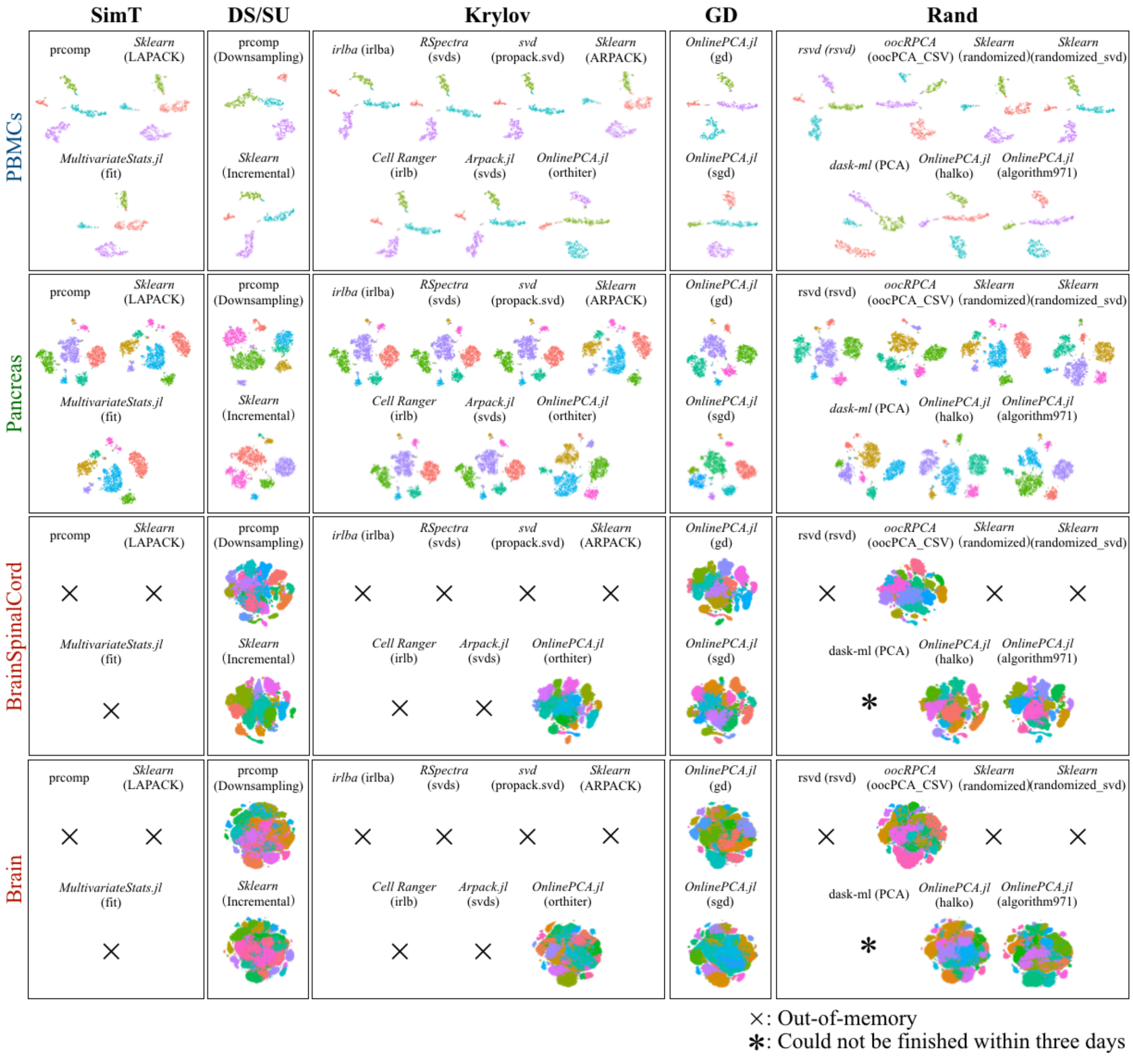

Figure S6-6 | Results of Louvain clustering The cluster label is visualized with two-dimensional t-SNE coordinates.

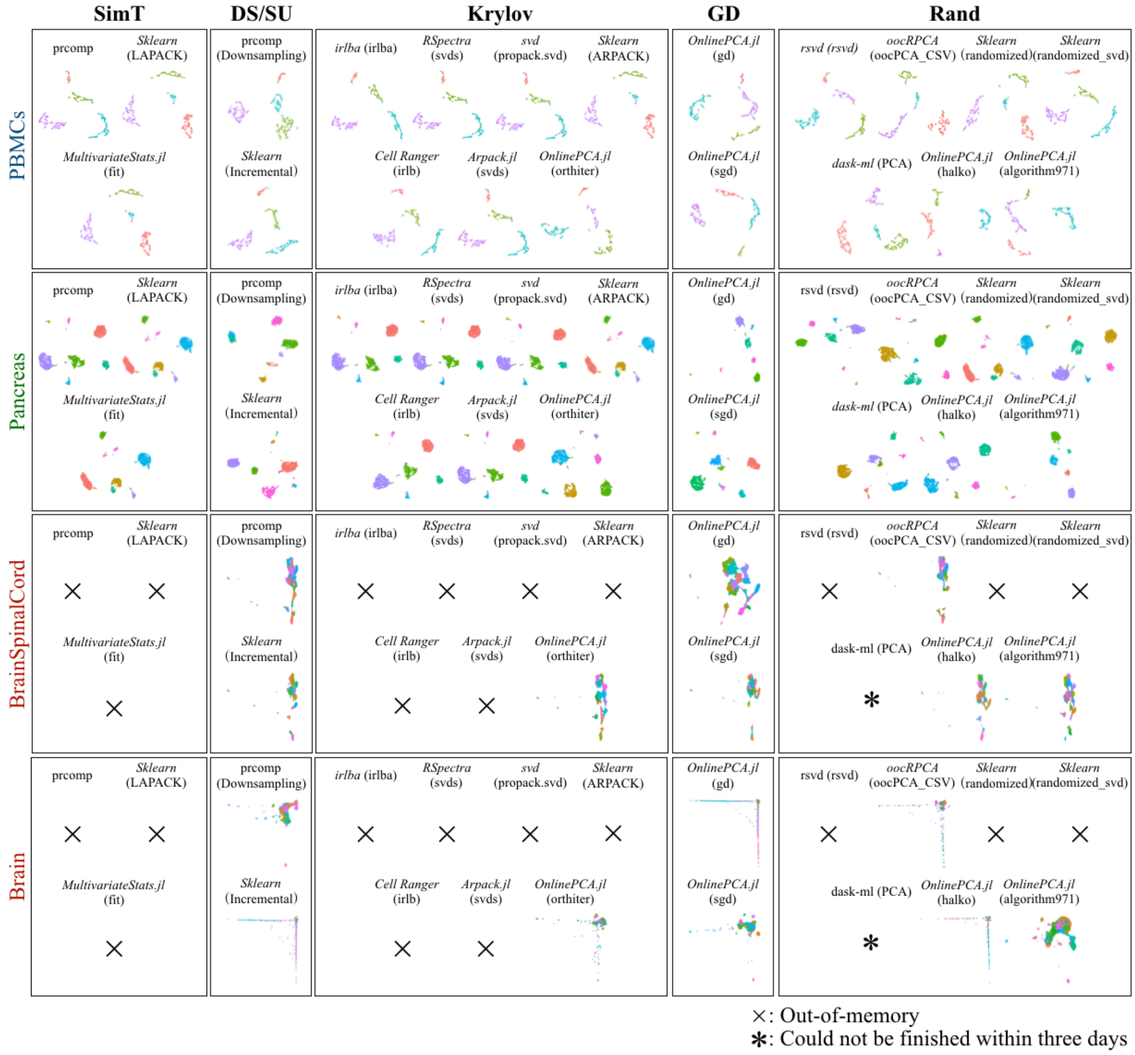

Figure S6-7 | Results of Louvain clustering The cluster label is visualized with two-dimensional UMAP coordinates.

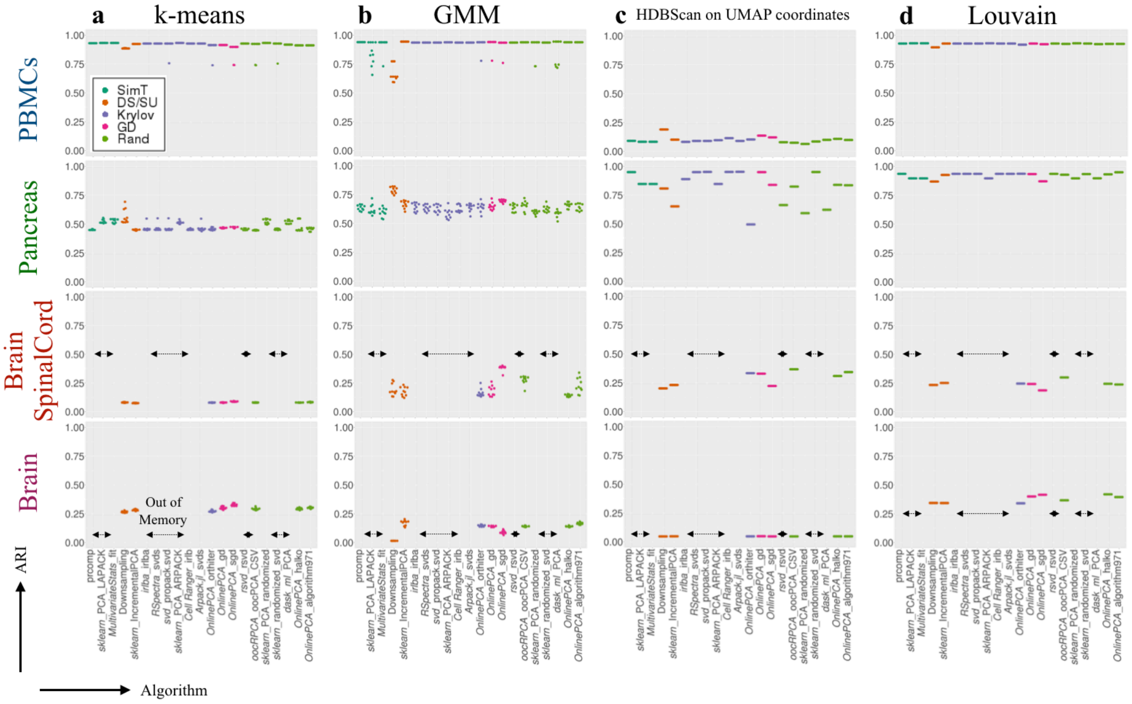

Figure S6-8 | ARI of all clustering results The algorithms used for benchmarking are listed along the *x*-axes; *y*-axes show associated ARI values.
