# Supplementary material for: Benchmarking principal component analysis for large-scale single-cell RNA-sequencing
Source: Genome Biol. 2020 Jan 20;21:9. doi: 10.1186/s13059-019-1900-3 (PMC6970290; doi:10.1186/s13059-019-1900-3)
Supplement: Supplementary file 22 — Additional file 22 Parameter tuning of the randomized sVD implementations. [file 13059_2019_1900_MOESM22_ESM.pdf]

Parameter tuning of the randomized SVD algorithms

Here, we perform parameter tuning of the randomized SVD algorithms examined, including Halko's method and algorithm971 [107-109]. These algorithms commonly use the preconditioning process called *power iteration*. This step sharpens the distribution of eigenvalues and enforces a more rapid decay of the singular values ([111] and **Additional file 2**).

We found that the number of iterations (***niter***) is critical to accuracy and at least three or more values are needed (**Figure S18-1** and **2**). Accordingly, for all randomized SVD implementations mentioned in the main manuscript, a value of 3 was used for the *niter* parameter.

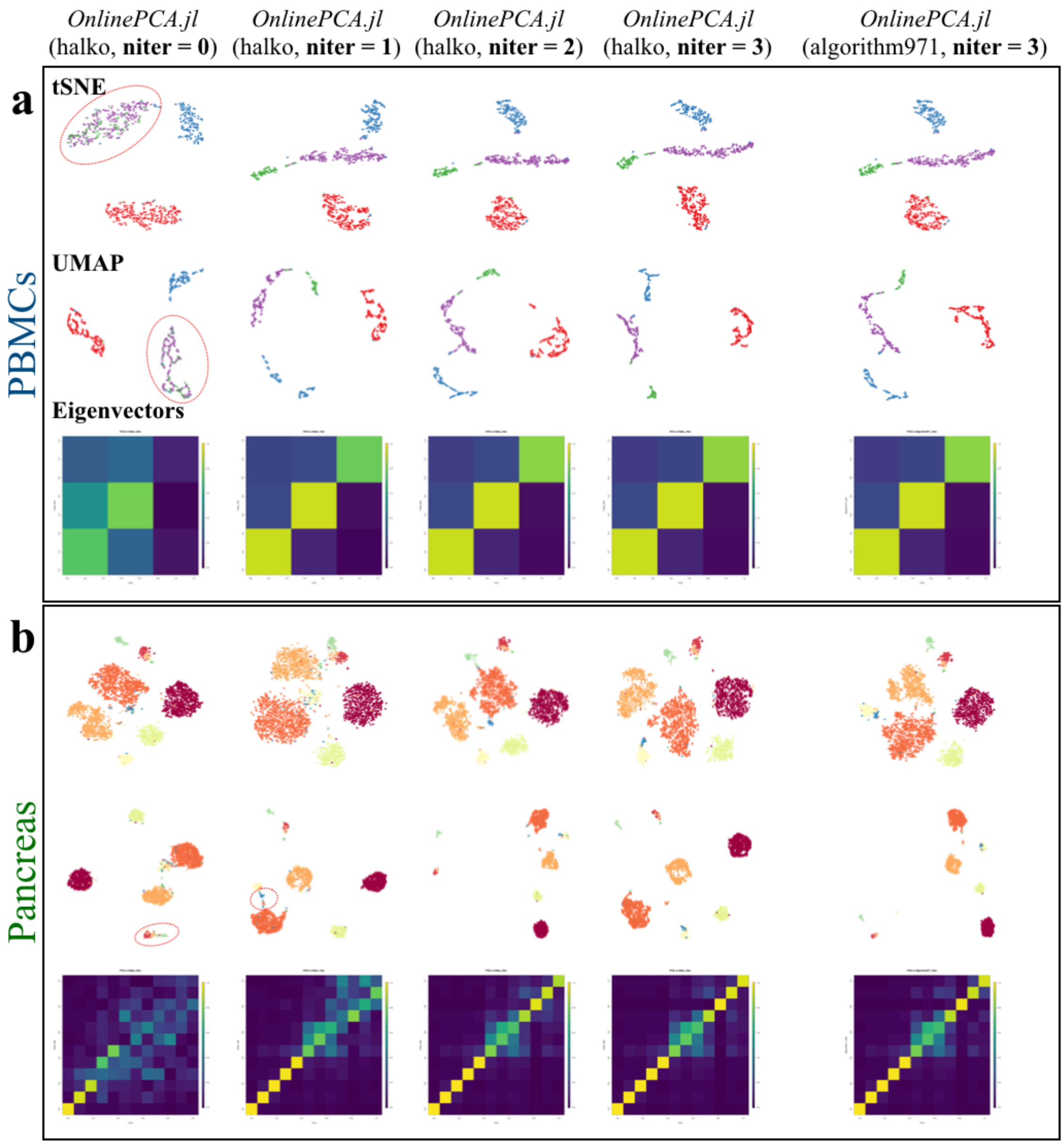

Figure S18-1 | Parameter tuning of Halko's method and algorithm971 (OnlinePCA.jl) (PBMCs and Pancreas datasets) The *niter* parameter was set at integer values from 0 to 3; the t-SNE appearance and cross-product plot used in the main manuscript were confirmed.

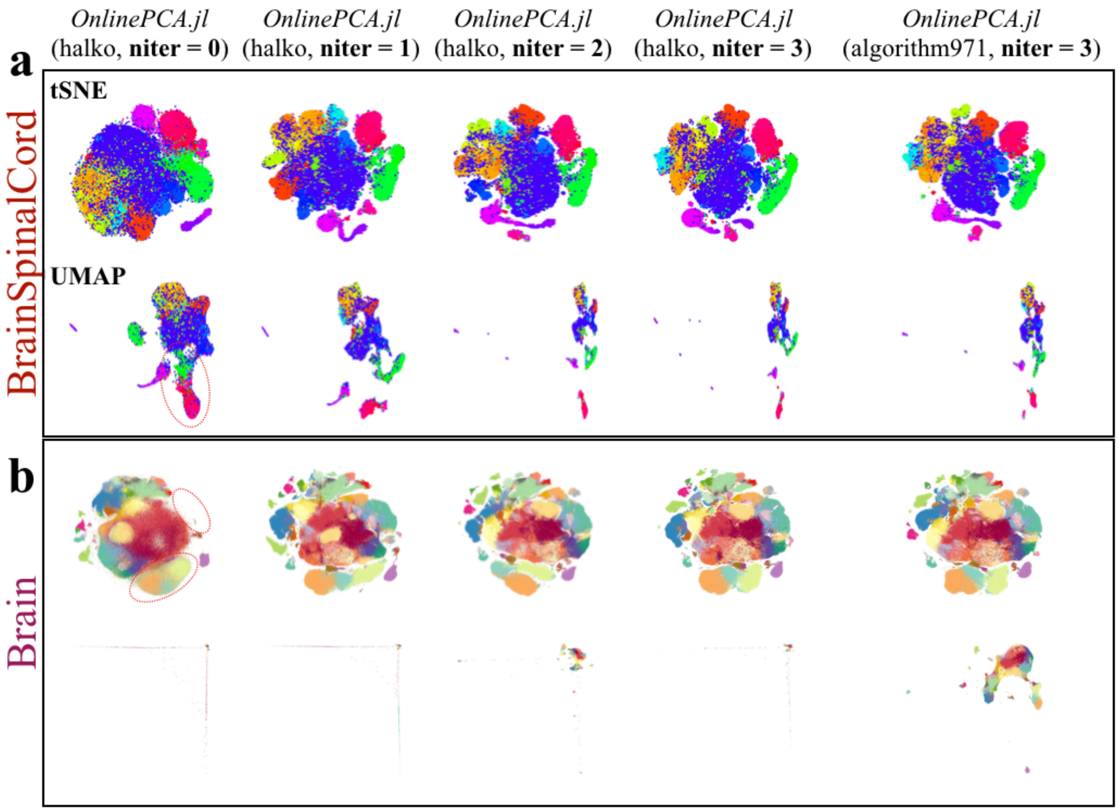

Figure S18-2 | Parameter tuning of Halko's method and algorithm971 (OnlinePCA.jl) (BrainSpinalCord and Brain datasets) The *niter* parameter was set at integer values from 0 to 3; the t-SNE appearance and cross-product plot used in the main manuscript were confirmed.
