# Supplementary material for: Benchmarking principal component analysis for large-scale single-cell RNA-sequencing
Source: Genome Biol. 2020 Jan 20;21:9. doi: 10.1186/s13059-019-1900-3 (PMC6970290; doi:10.1186/s13059-019-1900-3)
Supplement: Supplementary file 26 — Additional file 26 Review history. [file 13059_2019_1900_MOESM26_ESM.docx]

Review History

**First round of review**

**Reviewer 1**

**Are you able to assess all statistics in the manuscript, including the appropriateness of statistical tests used?**

Yes: The choice of pre-processing the scRNA-seq are not sound

**Comments to author:**

General comments:
The manuscript titled 'Benchmarking principal component analysis for large-scale single-cell RNA-sequencing' by the authors Tsuyuzaki et al. performed a benchmark comparison of principal component analysis (PCA) algorithms, including algorithms that work with data either in memory or on-disk data representations (e.g. HDF5 files). In addition, the authors extended some of the PCA methods previously proposed to work with data on-disk for four algorithms that previously did not work with. The topic of principal components analysis in application to scRNA-seq data is a timely and important topic and I am extremely eager to see thoughtful and thorough benchmarking comparisons for methods used in computational biology. However, I have several concerns that need to be addressed, which significantly lessens my enthusiasm about this particular manuscript. I've detailed my concerns below.

Specific comments:
Major:
- A major problem with this manuscript is the highly technical level of detail. The majority of this manuscript would not have outstanding interest to broad audience of biologists, or analysts of scRNA-seq data. For example, the first set of results titled "Review of PCA algorithms and implementations" (pgs 3-4) are two pages full of only technical details about the algorithms. However, I do like how there is some mention of which scRNA-seq methods/tools use various PCA approaches, but this could all be summarized in a table. This high level of technical detail continues throughout the manuscript and distracts from the fundamental results, summaries and take-aways that readers are looking for in benchmarking papers. This paper needs to be completely restructured to solve this problem.
- My second major concern are the steps used to pre-process the scRNA-seq data.
o     PCA is based on the assumption of normality. Most methods that use PCA with scRNA-seq first scale by sample- or cell-specific size factors, followed by log-transformation. This is supposed to transform the data such that the data look more normally distributed and to reduce the skewness of the distribution. However, it seems the authors are not using size factors (pg 11, line 57). Could the authors explain?
o     Second, recent work has shown that by using current normalization procedures and taking the log-transformation of scRNA-seq prior to PCA leads to false variability in dimension reduction (https://www.biorxiv.org/content/10.1101/574574v1). As the authors state this transformation is the basis of their entire benchmark comparison, I am severely concerned with this choice in transformation and have trouble trusting these results presented here. In fact, I would like to see the authors compare PCA algorithms that are based on raw counts (avoiding the problems with normalization log transformation e.g. GLM-PCA).
o     Another common step is to perform feature selection. Based on my understanding, the only feature selection being performed is to remove genes with only 0s across all cells? That is not really feature selection. Usually this is done by selecting the top 1000-2000 highly variable or highly expressed genes. Then PCA can reduce the e.g. 1000-dimensional expression profile to e.g. 15-dimensional vector of principal components. In addition, were ERCC spike-ins removed prior to PCA e.g. in the Cortex data? Could the authors explain why they are not applying feature selection as this is an unrealistic asessement they are performing?
o     Another common step is to center and scale the data prior to PCA so that each gene has mean zero and standard deviation 1. I also do not understand if this a transformation the authors are using in their benchmark comparison?
-     The three datasets used by the authors were processed on three different platforms: Cortex (96 well-plates using the full-length SMARTer protocol), Pancreas (inDrop) and Brain (10X Genomics). Recent work has shown that droplet-based protocols are not zero-inflated, but plate-based protocols do seem to be zero-inflated (https://www.biorxiv.org/content/10.1101/582064v1). In this manuscript, the authors treat these datasets the same and apply PCA algorithms that are not aware of zero-inflated data. I would like to see a comparison across more datasets that take into account the different protocols because this could be highly influential on the various performance metrics that the authors used e.g. in clustering.
-     For simulated data, the authors used the negative binomial model. Given the comment above, this would be relevant to UMI-based protocols, but not full-length protocols. Could the authors also include simulation studies for zero-inflated negative binomial models?
-     There are way too many figures in this manuscript. Some need to be removed entirely or moved to the supplement. Figure 2 is very detailed and I would suggest removing it. Figures 4, 6, 8 need to be replaced, transformed and summarized. It is impossible to get much from these figures. Figures 7, 10, 11 were also not very meaningful because I couldn't tell which color corresponded to which algorithm as there were so many algorithms being compared that the color gradient was too similar.  

Minor:
- There are citations missing, e.g. eigenvalue decomposition, singular value decomposition, R language, DGESVD, DGESDD, QR method, Householder transformation, to name a few.
- Why are the authors using t-SNE over umap/uwot? The later has been shown to be much faster and enables comparable embeddings vs t-SNE does not. In addition, what's the difference between performing PCA and then applying t-SNE or umap/uwot vs just applying umap/uwot to the data directly?
- Pg 8, line 28-29: "tenxpca can finish the calculation in 1.18 hours with 82.96 GB memory usage". Did the authors try this on different systems? Is this max memory or total memory? How are data stored in hdf5 file (chunk wise, column wise, row wise)? Does the data storage and access have an impact on the speed and memory performance reported by the PCA algorithms that use on-disk data representation?
- I am unclear on how the accuracy was evaluated for the Brain data. The author state in page 5, lines 37-48 that prcomp was not computationally feasible, so the result using Cell Ranger was used. But then the authors go on to state that Cell Ranger was not used and the output from 10X genomics was used. What algorithm is used by 10X genomics and why is it considered the gold standard?
- Pg 2, line 22. "2.0 million" --> "2 million"
- Pg 2, line 31. "not acceptable" --> "not be acceptable".
- Pg 3, line 33. "to be loaded" --> "to load"

**Reviewer 2**

**Are you able to assess all statistics in the manuscript, including the appropriateness of statistical tests used?**

Yes: All statistical analyses are appropriate.

**Comments to author:**

Nikaido and colleagues presented a comprehensive benchmarking analysis of principal component analysis (PCA) methods for large-scale scRNA-Seq data. The study is timely and carefully executed. The literature review on existing PCA methods is comprehensive. Ten algorithms with about 20 implementations were evaluated in terms of their accuracy, memory and time requirement using synthetic and three real scRNA-Seq data sets with varying sizes. The authors also provided their own implementation of online PCA in the Julia programming language. The authors provided user guidelines depending on the size of the data and preferred programming languages.  The manuscript was well written and organized, and the benchmarking was conducted appropriately. The following issues need to be addressed before it can be accepted for publication.
Major comments:
1.     The CellRanger implementation of PCA, the IRLBA algorithm, was chosen as the gold standard to measure accuracy for the Brain dataset. Please provide the justification for this choice of gold standard. Additionally, the cell clustering labels of original studies were used as true cell identities, which may lead to bias towards the IRLBA algorithm. because the original study this algorithm.
2.     Two standard clustering methods, K-means and Gaussian Mixture Model (GMM) clustering, were used in the analysis. As evaluation of the PCA performance relies on the clustering result, additional clustering methods such as graph-based clustering methods, need to be evaluated in combination with the different PCA methods.
3.     Out-of-core algorithms are promising solutions for very large-scale datasets. The authors implemented several algorithms in this category using Julia programming language, which is very helpful. It would be more helpful for the community if R/Python implementation in R/python can also be provided since R/Python are more widely used in the field.
4.     In Figure 4, t-SNE is performed using PCA results. Since the two methods are both dimension reduction techniques, by doing so, the benchmarking procedure is confounded by using t-SNE.
5.     It is mentioned that marker genes are used to evaluate the clustering quality of real data. No details are given regarding how marker genes are defined. Please add this information.

**Authors Response**

**Point-by-point responses to the reviewers’ comments:**

Response to Reviewer #1

Specific Comments:
Major:

> A major problem with this manuscript is the highly technical level of detail. The majority of this manuscript would not have outstanding interest to broad audience of biologists, or analysts of scRNA-seq data. For example, the first set of results titled "Review of PCA
algorithms and implementations" (pgs 3-4) are two pages full of only technical details about the algorithms. However, I do like how there is some mention of which scRNA-seq methods/tools use various PCA approaches, but this could all be summarized in a table. This high level of technical detail continues throughout the manuscript and distracts from the fundamental results, summaries and take-aways that readers are looking for in benchmarking papers. This paper needs to be completely restructured to solve this problem.

*We appreciate this helpful comment. Based on this comment, we have moved some of the particularly technical parts of the manuscript to Additional File 1. The case study of PCA in single-cell RNA-seq (scRNA-seq) data analysis that was explained in the Background section has been moved to the Results section.*

> PCA is based on the assumption of normality. Most methods that use PCA with scRNA-seq first scale by sample- or cell-specific size factors, followed by log-transformation. This is supposed to transform the data such that the data look more normally distributed and to reduce the skewness of the distribution. However, it seems the authors are not using size factors (pg 11, line 57). Could the authors explain?

*In the previous version of this manuscript, we prepared three real datasets including full-length scRNA-Seq (Smart-Seq for Cortex) and high-throughput scRNA-Seq (Drop-Seq for Pancreas and 10X Chromium for Brain) with cell barcodes and unique molecular identifiers (UMIs). Because the de facto standard normalization methods of both technologies differ (i.e., FPKM/TPM vs CPM), we unified the unit of gene expression as count values for both technologies. This choice was also based on our empirical knowledge that normalization is not critical for cell-level analyses of single-cell RNA-Seq. However, we have changed the real datasets to focus on only high-throughput scRNA-Seq data with cell barcodes and UMIs, and therefore, we introduced a cell-specific size factor normalization in the revised manuscript. The details are explained later.*

> Second, recent work has shown that by using current normalization procedures and taking the log-transformation of scRNA-seq prior to PCA leads to false variability in dimension reduction (https://www.biorxiv.org/content/10.1101/574574v1). As the authors state this transformation is the basis of their entire benchmark comparison, I am severely concerned with this choice in transformation and have trouble trusting these results presented here. In fact, I would like to see the authors compare PCA algorithms that are based on raw counts (avoiding the problems with normalization log transformation e.g. GLM-PCA).

*This bioRxiv manuscript claims that the use of CPM and subsequent log-transformation will separate the zero and non-zero data and that this gap leads to false variability in dimensional reduction. However, we confirmed such an effect is not as substantial as claimed when the normalization coefficient of size factor normalization is not large. We demonstrate this in Additional File 21. In addition, the R source code for GLM-PCA (https://github.com/willtownes/glmpca/blob/master/R/glmpca.R) cannot be performed for high-throughput scRNA-Seq such as the Brain dataset, and therefore, adding this method to our benchmark test is beyond the scope of this work. Although there is no available implementation to perform GLM-PCA for high-throughput scRNA-Seq, we have introduced this method as one of the future extensions of large-scale PCA in the Discussion section. We believe that our benchmarking is also useful for the implementation of such a special type of PCA algorithm, because the objective function of GLM-PCA is based on the maximum likelihood method and this function can be decomposed as the sum of the log-likelihood calculated from individual data points. Furthermore, the calculation might be performed in an out-of-core manner, as well as using GD-type PCA as described in the manuscript.*

> Another common step is to perform feature selection. Based on my understanding, the only feature selection being performed is to remove genes with only 0s across all cells? That is not really feature selection. Usually this is done by selecting the top 1000-2000 highly variable or highly expressed genes. Then PCA can reduce the e.g. 1000-dimensional expression profile to e.g. 15-dimensional vector of principal components. In addition, were ERCC spike-ins removed prior to PCA e.g. in the Cortex data? Could the authors explain why they are not applying feature selection as this is an unrealistic asessement they are performing?

*Using the Brain dataset, we investigated the effect on clustering accuracy of the number of genes extracted according to the p-values of highly variable genes and found that the clustering accuracy showed a saturation curve (Additional File 20). Although it is still difficult to decide how many genes should be kept in feature selection, this result suggests that selecting too few genes might decrease clustering performance. Considering the results of the experiment, we used all genes that have non-zero variance and are not ERCC spike-ins to simulate the worst case scenario for computational time and memory usage.

Please note that feature selection of extremely high-throughput scRNA-Seq is also not trivial. In our experiment, we used our original Julia source code to calculate highly variable genes in an out-of-core manner and to avoid out-of-memory errors. In actual data analysis, users might search for the appropriate number of genes by generating datasets of different sizes, but such trial-and-error steps will require multiple executions of PCA. Therefore, the acceleration and memory efficiency of PCA discussed in our manuscript are important, even if the dataset ultimately becomes small.*

> Another common step is to center and scale the data prior to PCA so that each gene has mean zero and standard deviation 1. I also do not understand if this a transformation the authors are using in their benchmark comparison?

*This is related to the eigenvalue decomposition (EVD) of a correlation matrix, not a covariance matrix. In many PCA implementations, however, the default PCA is based on EVD of the covariance matrix (e.g., prcomp, https://www.rdocumentation.org/packages/stats/versions/3.6.1/topics/prcomp; scikit-learn, https://scikit-learn.org/stable/modules/generated/sklearn.decomposition.PCA.html), and EVD of a correlation matrix seems to be a minor approach in scRNA-seq data analysis. Perhaps such an approach has merit in its avoidance of too much attention paid to genes with high average expression. However, we have already performed a variance-stabilization method (i.e., log-transformation), and we think such tuning does not critically impact the conclusions based on the results. In consideration of the above reasons and the trade-offs among the large number of methods to be evaluated and our finite resources, we focused only on the EVD of the covariance matrix. Even if each matrix is changed to a correlation matrix, our benchmark will be useful for achieving acceleration and memory efficiency.*

> The three datasets used by the authors were processed on three different platforms: Cortex (96 well-plates using the full-length SMARTer protocol), Pancreas (inDrop) and Brain (10X Genomics). Recent work has shown that droplet-based protocols are not zero-inflated, but plate-based protocols do seem to be zero-inflated (https://www.biorxiv.org/content/10.1101/582064v1). In this manuscript, the authors treat these datasets the same and apply PCA algorithms that are not aware of zero-inflated data. I would like to see a comparison across more datasets that take into account the different protocols because this could be highly influential on the various performance metrics that the authors used e.g. in clustering.

*The main purpose of our manuscript is the benchmarking of PCA implementations and is not investigating differences among experimental methods. Additionally, the manuscript focuses in particular on the scalability of PCA with high-throughput scRNA-Seq. High-throughput scRNA-Seq is mainly used with cell barcoding and UMI methods, which are important technologies for generating large-scale single-cell transcriptome datasets. As the reviewer have noted, UMI counts are not zero-inflated. For the above reasons, we focused only on the high-throughput scRNA-Seq datasets in the revised manuscript. We have newly prepared four empirical datasets: PBMCs, Pancreas, BrainSpinalCord, and Brain. We investigated the scalability of PCA implementations using these datasets.*

> For simulated data, the authors used the negative binomial model. Given the comment above, this would be relevant to UMI-based protocols, but not full-length protocols. Could the authors also include simulation studies for zero-inflated negative binomial models?

*We focused only on the datasets of high-throughput scRNA-Seq with UMI in the revised manuscript. As the reviewer have noted, UMI counts are not zero-inflated. We also consider the reviewer's comments to be an important point, but they are unfortunately beyond the scope of the present manuscript. We expect the reviewer to understand our focus on the scalability of PCA with high-throughput scRNA-Seq with UMI technology.*

> There are way too many figures in this manuscript. Some need to be removed entirely or moved to the supplement. Figure 2 is very detailed and I would suggest removing it. Figures 4, 6, 8 need to be replaced, transformed and summarized. It is impossible to get much from these figures. Figures 7, 10, 11 were also not very meaningful because I couldn't tell which color corresponded to which algorithm as there were so many algorithms being compared that the color gradient was too similar.

*We appreciate this comment. As suggested, we have reorganized the figures as follows (previous manuscript → revised manuscript).

- Figure 2 → Moved to Additional File 1.
- Figure 4 → The full version of the t-SNE plots is moved to Additional File 4, and the only representative implementations are kept in Figure 3.
- Figure 6 → The full version of the eigenvector plots is moved to Additional File 7, and only representative implementations are kept in Figure 5.
- Figure 7 → The eigenvalue plots are stratified based on the categories we defined in the manuscript.
- Figure 8 → The full version of the loading vector plots is moved to Additional File 14, and only representative implementations are shown in Figure 5.
- Figure 10 and 11 → We changed the color map to a darker one to make the figure easier to interpret.*


Minor:

> There are citations missing, e.g. eigenvalue decomposition, singular value decomposition, R language, DGESVD, DGESDD, QR method, Householder transformation, to name a few.

*We appreciate this comment. We have added citation [90] for EVD, SVD, DGESVD, DGESDD, QR, and Householder transformation. We have also added the citations [111], [112], and [113] for R, Python, and Julia languages, respectively. Please check Additional File 1.*

> Why are the authors using t-SNE over umap/uwot? The later has been shown to be much faster and enables comparable embeddings vs t-SNE does not. In addition, what's the difference between performing PCA and then applying t-SNE or umap/uwot vs just applying umap/uwot to the data directly?

*We appreciate the helpful advice. We have added the results of UMAP to the benchmark. We could not precisely understand the intention of the comment on "just applying umap/uwot to the data directly," but we assumed that addressing this entails the direct construction of a cell-cell distance matrix without performing PCA. For high-throughput scRNA-Seq, however, all the elements of the matrix cannot be loaded into memory. Therefore, the calculation of a cell-cell distance matrix itself is difficult without compressing the data dimensionality.

Even if the data matrix can be loaded into the memory, in many implementations of t-SNE and UMAP, PCA is first performed (e.g., Rtsne, https://github.com/jkrijthe/Rtsne/blob/c81503a72f39e32350153554f0c68945bc4fea3f/R/Rtsne.R#L121; uwot, https://github.com/jlmelville/uwot/blob/741814157b748e56ae8ca38c8788f0da76f6eaf5/R/init.R#L237). This is probably because when the data dimensionality is extremely high, the performance of most of the data analysis methods becomes worse, which is a well-known problem called the "curse of dimensionality." Therefore, in this work, we show only the results of t-SNE/UMAP after PCA. We have added citations [78-81] for the curse of dimensionality in single-cell data analysis.*

> Pg 8, line 28-29: "tenxpca can finish the calculation in 1.18 hours with 82.96 GB memory usage". Did the authors try this on different systems? Is this max memory or total memory?

*We used the same machines described in the Computational environment section. We modified "82.96 GB memory usage" to "83.0 GB maximum memory usage."*

> How are data stored in hdf5 file (chunk wise, column wise, row wise)? Does the data storage and access have an impact on the speed and memory performance reported by the PCA algorithms that use on-disk data representation?

*In CellRanger, the expression matrix data is formatted as a Compressed Sparse Column (CSC) sparse matrix (cf. https://support.10xgenomics.com/single-cell-gene-expression/software/pipelines/latest/advanced/h5_matrices#header). A CSC sparse matrix is expressed as three objects: data, indices, and indptr. Using these objects, not all the elements of a matrix are explicitly stored, which means only non-zero elements are stored. Therefore, in such a memory-efficient matrix structure, the chunk size is not defined in an HDF5 file (contiguous chunk, https://www.star.nesdis.noaa.gov/jpss/documents/HDF5_Tutorial_201509/2-2-Mastering%20Powerful%20Features.pptx.pdf). The tenxpca command incrementally converts a small block of a sparse matrix into a dense matrix, and we have already investigated the relationship between block size and calculation time/memory usage (Figure 10).*

> I am unclear on how the accuracy was evaluated for the Brain data. The author state in page 5, lines 37-48 that prcomp was not computationally feasible, so the result using Cell Ranger was used. But then the authors go on to state that Cell Ranger was not used and the output from 10X genomics was used. What algorithm is used by 10X genomics and why is it considered the gold standard?

*We apologize for this lack of clarity in our manuscript. We meant that the result of CellRanger (IRLBA) provided by 10X Genomics was used, and we did not use CellRanger ourselves. This is because the CellRanger computation for the Brain dataset is very memory intensive, and even with our largest memory machine (512 GB), we could not perform it.

In the previous version of this manuscript, we set the CellRanger result as the gold standard, because there are no other available accurate PCA implementations. However, we found that the results of CellRanger seem to differ from those of the other PCA implementations, and as we mentioned in the manuscript, such differences might be caused by differences in preprocessing steps, such as feature selection, scaling, or normalization of the data matrix, which are not well explained on the 10X Genomics website. Owing to such uncertainties, we now think we should not regard CellRanger as the gold standard for the Brain dataset.

In the revised manuscript, we have evaluated some scalable PCA methods for the Brain dataset (and also the BrainSpinalCord dataset) without setting a gold standard. According to the benchmarking guidelines developed by Mark D. Robinson's group (Weber et al., 2019), comparing methods against each other is recommended when the ground truth result cannot be defined. Therefore, we just compared the eigenvalues (Figure 6) and cluster accuracy of those methods (Figure 4). We also performed a comparison of eigenvectors in all combinations of the scalable PCA methods (Additional File 13).*

> Pg 2, line 22. "2.0 million" --> "2 million"
> Pg 2, line 31. "not acceptable" --> "not be acceptable".
> Pg 3, line 33. "to be loaded" --> "to load"

*We have modified each part accordingly. We appreciate Reviewer #1 pointing out these issues.*


Response to Reviewer #2
The original comments from Reviewer #2 are written in orange italics. Our responses to the comments are in black. Please check the attached file R2R_OnlinePCA.jl.pdf.

Major comments:

> 1. The CellRanger implementation of PCA, the IRLBA algorithm, was chosen as the gold standard to measure accuracy for the Brain dataset. Please provide the justification for this choice of gold standard. Additionally, the cell clustering labels of original studies were used as true cell identities, which may lead to bias towards the IRLBA algorithm. because the original study this algorithm.

*For the Brain dataset, prcomp could not be performed owing to an out-of-memory error. Therefore, we selected the result of CellRanger (IRLBA) provided by 10X Genomics as the gold standard instead. However, as Reviewer #2 points out, the result might be biased towards IRLBA, which is an approximate PCA algorithm. Additionally, we found that the results of CellRanger seem different from those of the other PCA implementations, and as we mentioned in the manuscript, the difference might be caused by differences in preprocessing steps, including feature selection, scaling, or normalization of the data matrix, which are not well explained on the 10X Genomics website. Owing to such uncertainties, we now think we should not regard CellRanger as the gold standard for the Brain dataset. In the revised manuscript, we have evaluated some scalable PCA methods for the Brain dataset (and also the BrainSpinalCord dataset) without setting a gold standard. According to the benchmarking guidelines developed by Mark D. Robinson's group (Weber et al., 2019), comparing methods against each other is recommended when the ground truth result cannot be defined. Therefore, we compare only the eigenvalues (Figure 6) and clustering accuracy of those methods (Figure 4). We also performed a comparison of eigenvectors in all combinations of the scalable PCA methods (Additional File 13).*

> 2. Two standard clustering methods, K-means and Gaussian Mixture Model (GMM) clustering, were used in the analysis. As evaluation of the PCA performance relies on the clustering result, additional clustering methods such as graph-based clustering methods, need to be evaluated in combination with the different PCA methods.

*In addition to k-means and GMM, we have added Louvain clustering, which is a graph-based clustering method. We have also added HDBScan, which is a density-based clustering method. These results are summarized in Additional File 6.*

> 3. Out-of-core algorithms are promising solutions for very large-scale datasets. The authors implemented several algorithms in this category using Julia programming language, which is very helpful. It would be more helpful for the community if R/Python implementation in R/python can also be provided since R/Python are more widely used in the field.

*Some of the out-of-core algorithms are already available for R/Python, such as oocRPCA (R) and IncrementalPCA (Python), and these implementations showed good performance in our benchmarking. Please check our guidelines for users (Figure 12).*

> 4. In Figure 4, t-SNE is performed using PCA results. Since the two methods are both dimension reduction techniques, by doing so, the benchmarking procedure is confounded by using t-SNE.

*We could not precisely understand the intention of this comment, but we have intuited that Reviewer #2 might have misunderstood that the clustering methods were performed on the two-dimensional t-SNE coordinates. In this work, t-SNE was used to visualize the clustering results, which were performed on the coordinates of each PCA algorithm, not on the t-SNE coordinates.*

> 5. It is mentioned that marker genes are used to evaluate the clustering quality of real data. No details are given regarding how marker genes are defined. Please add this information.

*We used the cluster labels, which were defined in the original papers of the datasets, to evaluate clustering accuracy. The procedures used to construct the labels are explained in their papers. Please refer to the references cited in the Materials and methods section.*

**Second round of review**

**Reviewer 2**

The authors have addressed my main comments satisfactorily.
Additional minor issues below need to be addressed before the manuscript can be accepted for publication.

1. There are too many figures (12). Figures 7-11 are not critical for reading the manuscript and can be moved to the supplementary materials. Since figure 2 is a summary of the entire work, it should be placed at the end of the figures, e.g. figure 6.

2. Many of the figure legends are too brief without enough details to help understand them. For instance, in figure 4, are the values shown the average of ten Louvain clustering runs; Similarly, no legend for the heatmap for figure 5.

3. What are circled clusters in figure 3?

**Authors Response**

**Point-by-point responses to the reviewers’ comments:**

**Reviewer 2**

> 1. There are too many figures (12). Figures 7-11 are not critical for reading the manuscript and can be moved to the supplementary materials. Since figure 2 is a summary of the entire work, it should be placed at the end of the figures, e.g. figure 6.

*We moved Figures 8 – 11 to Additional Files. Since the elapsed time and memory usage are also important topics in this work, we kept Figure 7 in the main manuscript. Although Figure 2 is the summary of the benchmarking test, this figure is also the list of all the PCA algorithms and implementation investigated in the first subsection of Result. For that reason, we kept the position of Figure 2 the same as before.*

> 2. Many of the figure legends are too brief without enough details to help understand them. For instance, in figure 4, are the values shown the average of ten Louvain clustering runs; Similarly, no legend for the heatmap for figure 5.

*[Figure 4]*

*We added the additional explanation as below;*

*“For each PCA result, Louvain clustering calculations were performed ten times and the average values were calculated.”*

*[Figure 5]*

*We added the color scale to Additional File 13.*

*We also added the additional explanation to the legend of Figures 6, 7, and 9.*

*> 3. What are circled clusters in figure 3?*

*We added the additional explanation as below to the main manuscript and the legend of Figure 3;*

*“Some distinct clusters determined by the original studies were incorrectly combined into single clusters (red circled cluster).”*
